# Inertia of Technology Stocks: A Technology-Explicit Model for the Transition toward a Low-Carbon Global Aluminum Cycle

**DOI:** 10.1021/acs.est.4c00976

**Published:** 2024-05-21

**Authors:** Moritz Langhorst, Romain Guillaume Billy, Christian Schwotzer, Felix Kaiser, Daniel Beat Müller

**Affiliations:** † Industrial Ecology Programme, Department of Energy and Process Engineering, Norwegian University of Science and Technology, Trondheim 7034, Norway; ‡ Department for Industrial Furnaces and Heat Engineering, RWTH Aachen University, Aachen 52064, Germany

**Keywords:** Material flow analysis, aluminum, smelter, recycling, stock dynamics, greenhouse gases, climate change mitigation, industrial assets, lifetime

## Abstract

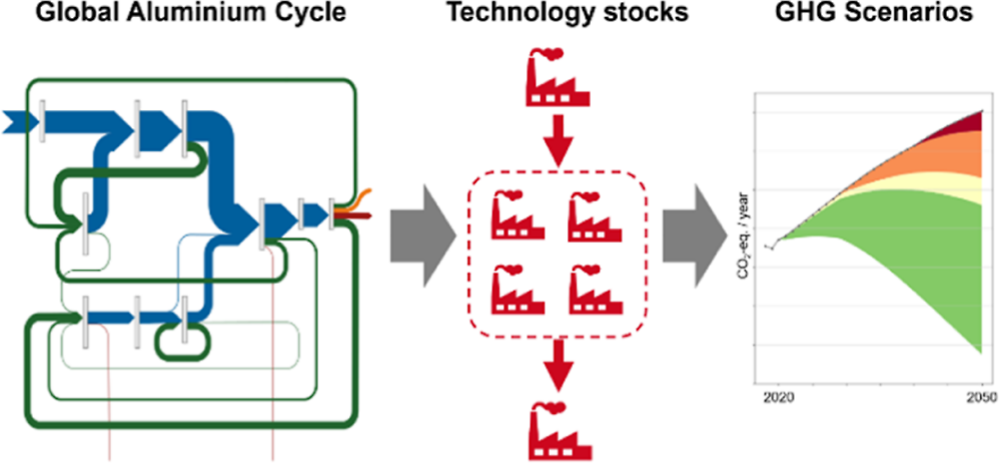

Low-carbon technologies are essential for the aluminum industry to meet its climate targets despite increasing demand. However, the penetration of these technologies is often delayed due to the long lifetimes of the industrial assets currently in use. Existing models and scenarios for the aluminum sector omit this inertia and therefore potentially overestimate the realistic mitigation potential. Here, we introduce a technology-explicit dynamic material flow model for the global primary (smelters) and secondary (melting furnaces) aluminum production capacities. In business-as-usual scenarios, we project emissions from smelters and melting furnaces to rise from 710 Mt CO_2_-eq./a in 2020 to 920–1400 Mt CO_2_-eq./a in 2050. Rapid implementation of inert anodes in smelters can reduce emissions by 14% by 2050. However, a limitation of emissions compatible with a 2 °C scenario requires combined action: (1) an improvement of collection and recycling systems to absorb all the available postconsumer scrap, (2) a fast and wide deployment of low-carbon technologies, and (3) a rapid transition to low-carbon electricity sources. These measures need to be implemented even faster in scenarios with a stronger increase in aluminum demand. Lock-in effects are likely: building new capacity using conventional technologies will compromise climate mitigation efforts and would require premature retirement of industrial assets.

## Abstract 2

This study proposes updated emissions scenarios for the aluminum sector by considering scrap recycling and delays of technology penetration due to the long lifetimes of current industrial assets.

## Introduction

To limit global warming to well below 2 °C according to the Paris Agreement, greenhouse gas (GHG) emissions must be reduced close to zero by 2050.^1^ To achieve this, clean energy technologies need to be deployed rapidly, especially in the industry sector, which was with 38% the largest contributor to global fuel combustion emissions in 2021.^2^ This transformation can be delayed due to the typically long lifetimes and high investment costs of industrial assets.^1^ The inertia of existing global infrastructure and its implications for future GHG emissions have been assessed in several publications.^3−7^ However, emission scenarios published by the Intergovernmental Panel on Climate Change (IPCC)^8^ and the International Energy Agency^1^ do not explicitly quantify this inertia and instead rely on projected changes in energy demand and carbon intensity over time.^3^

One important material for the transition to a low-carbon economy is aluminum, but its production is energy-intensive and generates GHG emissions, both directly (e.g., process and fossil fuel-related emissions) and indirectly (electricity).^9,10^ In 2018, the global aluminum industry accounted for about 1.1 Gt CO_2_-eq, including up- and downstream processes from mining to semiproduction,^11^ corresponding to ∼2.2% of the total GHG emissions. Electrolysis and recycling alone account for 1.5% of global GHG emissions.^12^ Reducing this share is difficult: the aluminum sector is considered a “hard to abate” industry^13^ and is currently not on track with the needed trajectories to reach Net Zero Emissions by 2050.^10^ Additionally, the global demand for aluminum products has been increasing by ∼42% from 2010 to 2020 and is projected to increase further due to global population growth and the role of aluminum in the clean energy transition.^14^ Forecasts predict that the in-use stock per capita of aluminum products will increase from 147 kg/capita in 2020 to 285–380 kg/capita in 2050.^14^ These figures underline the need for a rapid transition toward low-carbon aluminum production to reach climate targets and avoid further lock-ins.

To systematically assess the historical and future flows of materials, energy, and emissions associated with material cycles, material flow analysis (MFA) is widely used.^15^ In particular, the anthropogenic aluminum cycle and its interactions with the environment have been quantified in depth with MFA models.^16,17^ Dynamic MFA has also been used to design scenarios for the evolution of this cycle or specific products, based on stock-lifetime-driven models on global^18−26^ and regional scales.^19,27−32^ The usefulness of this approach is also recognized by the aluminum industry: in the last ten years, the International Aluminum Institute has been using dynamic stock-lifetime-driven MFA for its historical and forecasting model.^14^ Some studies have analyzed mitigation scenarios for the aluminum sector.^18,22,23,33^ However, they do not explicitly consider the evolution of stocks of production capacity and the potential delays and lock-ins due to the long lifetimes and high investment costs of the existing fossil infrastructure. Instead, they assume that emission intensities change over time, which does not hold for emissions that depend on the age of the technology.

Technology stocks have been modeled explicitly for other sectors. Pauliuk et al.^34^ looked at primary steel production facilities as stocks with a lifetime to explore how their development was affected by changes in demand. However, they did not consider the influence of the penetration of new technologies on GHG emissions reduction. The Mission Possible Partnership^35^ used an economic optimization model to model the penetration of new technologies in the aluminum industry. Although they considered the inertia of industrial assets by including asset lifetimes, the impact of different lifetimes, and penetration rates of technologies on emissions were not further investigated.

Here, we develop a framework for technology-explicit dynamic MFA models. Production capacities are considered stocks composed of different technologies that evolve over time and are linked to the dynamics of the in-use stocks. The stock dynamics of industrial assets are then modeled using a stock-lifetime-driven approach. This allows us to explore the inertia of aluminum production capacities and model the penetration of emerging low-carbon technologies.

## Methods

### System Definition

#### Global Aluminum Cycle

**Figure 1:**
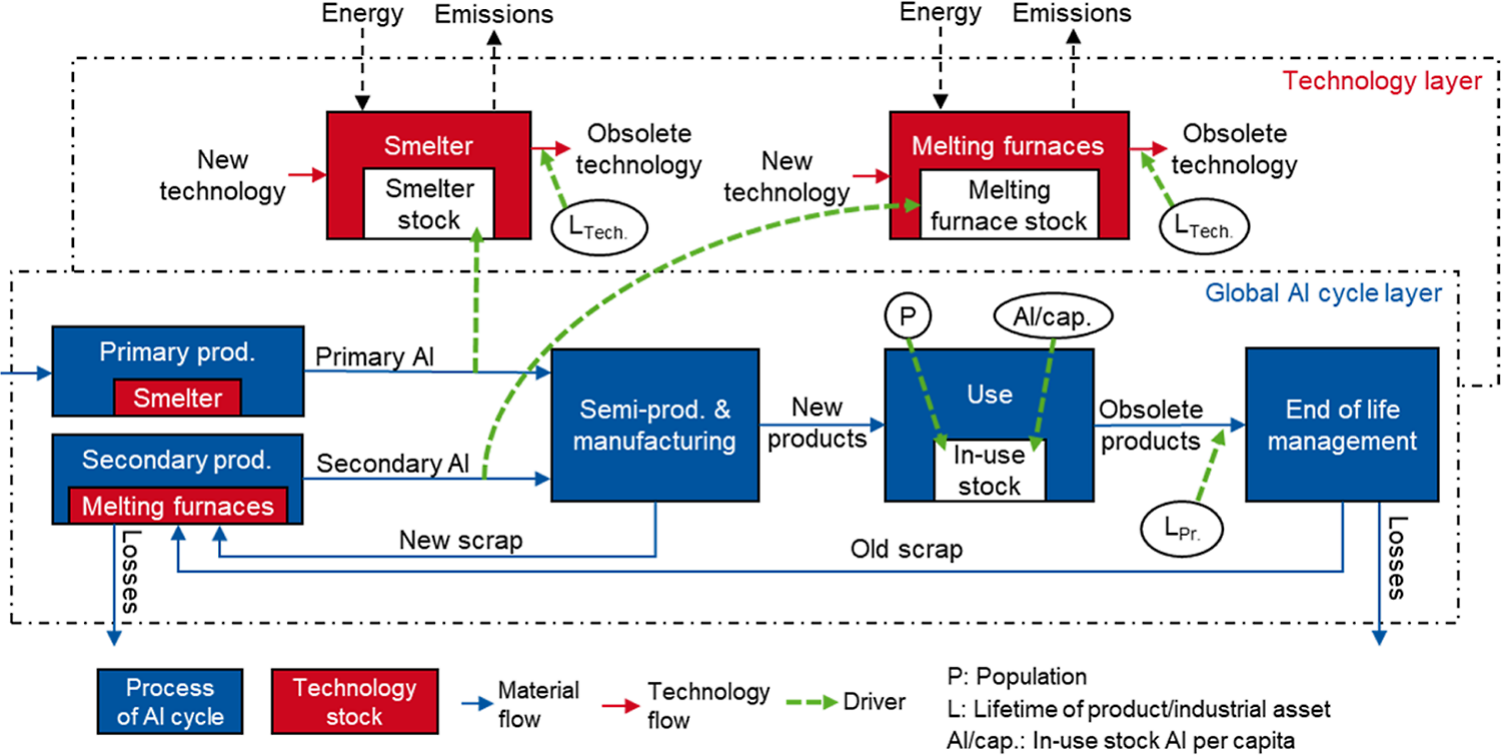
Simplified system definition and drivers for the technology-explicit aluminum cycle. Global aluminum cycle is driven by population, per capita in-use stocks of aluminum, and product lifetimes. Technology stocks (production capacities of different technologies) are driven by the demand for primary and secondary aluminum from the aluminum cycle layer. Lifetimes of industrial assets describe when technology stocks are becoming obsolete. Types of production technologies and their ages influence the energy demand and the GHG emissions. More detailed system definition is shown in Supporting Information, A.1–3.

**Figure 2:**
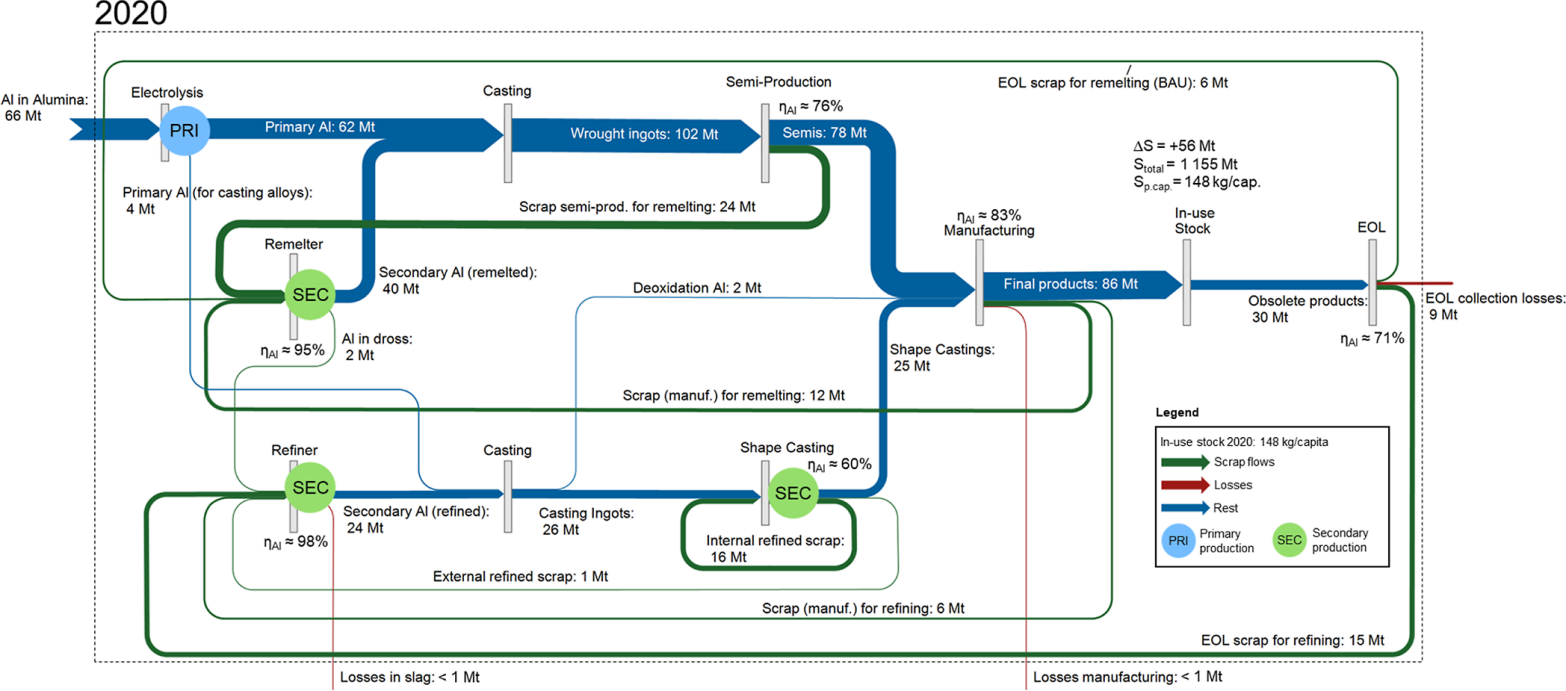
Global aluminum cycle in 2020. Flows are shown for aluminum content only. Scrap flows are shown in green, losses in red and all other flows, such as liquid metal and (semi-) products, in blue. Semi-product and final product flows are further differentiated into 9 semi-product categories and 12 final product categories.

**Figure 3:**
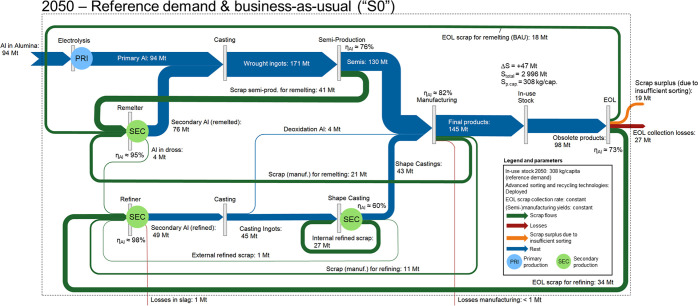
Global aluminum cycle in 2050 for baseline scenario. This assumes reference demand and constant future EOL scrap collection rates and processing yields. Orange flow marks the scrap surplus due to insufficient sorting of the different alloys. When advanced sorting and recycling technologies are deployed, it could be recycled to wrought alloys. Other scrap flows are shown in green, losses in red, and all other flows, such as liquid metal and (semi-) products, in blue.

We modeled the aluminum cycle from primary production to use and recycling and linked the production capacities to the associated technology stocks (Figure 1). Production and manufacturing processes and flows are shown in more detail in Figures 2 and 3; this includes the processes of electrolysis, remelter, refiner, casting, semi-production, shape casting, and manufacturing, as well as in-use stock and end-of-life (EOL) management. Upstream primary production processes, such as bauxite mining, alumina refining, and anode production, were not included within the system boundaries. The time horizon of the model ranges from 1900 to 2050. Aluminum products were further differentiated into 9 semiproducts and 12 final product categories (see Supporting Information, A.1) based on Liu et al.^18^ Recycling includes new scrap from semiproduction and manufacturing and old scrap from obsolete products.

The system differentiates production routes for wrought (processes remelter, casting, and semi-production) and casting alloys (processes refiner, casting, and shape casting). Wrought alloys are work-hardened in rolling or extrusion processes and are either produced from primary aluminum and/or by remelting new scrap or clean and separated postconsumer scrap. Contaminated and mixed postconsumer scrap is recycled in refiners to produce casting alloys. Scrap produced during shape casting is mainly recycled internally in the foundries. Obsolete products are collected, sorted, and separated in the EOL management process and allocated to the different recycling processes.^36−38^ Mixed or contaminated postconsumer scrap is recycled in refiners to produce casting alloys.^18,36,39^ Using it to produce wrought alloys would require additional separation and alloy-sorting methods, such as improved sorting infrastructure, robotics, sensor-based sorters, or the use of eddy current separation.^40^

#### Technology Stocks

Technology stocks are defined as the production capacities needed to meet the material demand for primary and secondary aluminum. They were assumed to have three dimensions: types (the type of technology used), cohorts (the year of construction), and time (the modeling year). Specific energy demands and emission intensities were differentiated by cohorts and types.

Primary aluminum is produced from alumina in an electrolytic reduction process (smelting), during which carbon anodes are consumed. The carbon reacts with oxygen in the alumina, leading to CO_2_ emissions. Other direct GHG emissions are perfluorocarbon (PFC) emissions due to carbon reacting with the fluor from the bath. Electrolysis technologies differ regarding the type of anodes used and the matter in which the alumina is fed. Today, most smelters use prebaked anodes (ca. 95%) while a few Søderberg smelters are still in use. The prebake technology uses multiple prebaked anodes per cell, which need to be replaced after consumption. In Søderberg cells, an anode paste is consumed continuously.^37^

Aluminum scrap is melted in remelters and refiners. The melting furnaces are mainly heated with natural gas. Electrically heated furnaces are already in use and state of the art, but are less common due to lower capacities, process limitations, and often higher energy costs;^36−38^ especially refiners seldom use electrically heated furnaces today.^36,38^

### Drivers and Model Approaches

#### Stock Dynamics

We used a stock-lifetime-driven dynamic model^41^ to describe the dynamics of the in-use stocks and the aluminum cycle.^18^ The different scrap flows were allocated to the recycling processes (remelter and refiner) to supply the casting and semi-production processes with secondary aluminum. The primary aluminum demand was derived from the remaining metal demand not covered by the secondary aluminum. The stock dynamics of the technology assets were also described using a stock-lifetime-driven dynamic model; however, these stocks are driven by the demand for primary and secondary aluminum. We therefore assumed that the production capacity was adjusting to the demand (see A.1 in the Supporting Information for more details on the modeling). We assumed a normally distributed lifetime with a mean of 40–50 years for the smelters and 20–30 years for melting furnaces (see A.2–3 in the Supporting Information). When production capacities reach their EOL, they are assumed to retire (outflows of technologies). Inflows of new technologies cover the replacement of obsolete capacities and the need for increasing production capacities in cases of growing demand.

#### Transfer Coefficients

We used transfer coefficients based on existing literature^18,36,42−44^ to allocate the final products to product categories to allocate EOL scrap between remelters and refiners, and to calculate the various losses throughout the system (Supporting Information, A.1).

The primary and secondary production (marked with “PRI” and “SEC” in Figures 2 and 3) determined in the aluminum cycle were then used in a second step to define the production capacity of the technology layer (Figure 1). Since the actual throughput of industrial assets is usually below the maximum capacity,^45^ a utilization rate was used for the smelters based on the literature and assumptions.^46−48^ Due to missing data, we did not include a utilization rate for the secondary production.

#### Energy and Emissions

Energy demand for primary production and melting was calculated based on specific energy consumption for technology types and cohorts and the aluminum throughput of the electrolysis, remelters, and refiners. While direct CO_2_ emissions from anode consumption were assumed to stay constant over time, specific direct PFC emissions were assumed to be cohort-dependent. Indirect emissions were calculated based on the total electricity demand of the smelters, the global smelter-specific electricity-mix compiled by the IAI,^49^ and the carbon intensities of the energy carriers listed by the IPCC.^50^ GHG emissions from melting were calculated based on the energy demand for melting, a constant emission factor for natural gas heated furnaces,^51,52^ and the global electricity-mix.^53^ (see Supporting Information, A.2–3).

### Data and Uncertainties

We designed different demand and parameter scenarios to account for the uncertainties in modeling the aluminum cycle and the associated energy demand and emissions. We also performed a multidimensional sensitivity analysis to quantify the impacts on the results of the parameters chosen for asset lifetimes, market penetration rates (MPRs), and retrofitting rates. Assumptions are listed in more detail in Supporting Information, A.1–A.3.

### Scenarios

#### Global Aluminum Cycle

Future projections for the global per capita in-use stock were based on two scenarios, a reference demand scenario and a high demand scenario. We used these scenarios to explore the impact of different demand scenarios on the speed of technology penetration. In the reference demand scenario, in-use stock increases from ∼148 kg/capita in 2020 to ∼308 kg/capita by 2050 while 390 kg/capita is reached in the high demand scenario. The scenarios are based on different assumptions on saturation levels and years of the per capita in-use stock (see Supporting Information, A1).

Today, most of the postconsumer (EOL) scrap is recycled through refiners due to the stronger constraints that remelters have in terms of alloy compositions and impurities.^42^ The availability of postconsumer scrap could increase to the point that it exceeds the demand for casting alloys, making a surplus of EOL scrap likely.^20,54,55^ If advanced sorting and recycling processes were developed or new applications for mixed scrap were found,^9,56^ this scrap could also be used in the remelters. For this purpose, we used the parameter “advanced sorting and recycling technologies”. In scenarios where advanced technologies are deployed, we assumed that there would be no quality constraints for recycling of postconsumer scrap in the remelters. EOL-collection rates (∼71% in 2020) as well as (semi-) manufacturing yields (∼60–83% in 2020) were assumed to develop in two ways. In the business-as-usual (BAU) scenario, they would stay on the 2020 level for all (semi-) product categories. In the increasing development scenario, scrap collection rates and yields of the different (semi-) product categories were assumed to gradually increase to 90–95% by 2050.^18^

#### Technology Stocks

Scenarios for the penetration of new technologies are described in Table 1. Inert anodes, as an alternative to the carbon anodes used today, are one of the most promising options to reduce direct emissions in primary production.^57^ Although they have been the focus of research for decades, only prototypes have reached commercial scale, and a technology readiness level (TRL) of 4–7 is reported.^35,58^ We assumed that inert anodes would lead to zero direct emissions. We designed different scenarios for the future use of inert anodes; one where no inert anodes would be used in the future and different combinations of market penetration and retrofitting rates of inert anodes from 2030 onward.^35,59^

**Table 1:** Scenarios for Technology Penetration Rates, Retrofitting, and Lifetime for Smelters and Melting Furnaces

parameters		scenarios	description
smelters	lifetime	low	40 years mean. Standard distribution with a standard deviation of 1/3 of mean value
		high	50 years mean. Standard distribution with a standard deviation of 1/3 of mean value
	penetration rates of inert anodes	no inert anodes	no inert anodes are used
		low	20% of smelter capacity inflows are using inert anodes in 2030, increasing linearly up to 80% in 2040
		high	100% inert anodes in smelter capacity inflows from 2030 onward
	retrofitting rates of inert anodes	no retrofitting	no retrofitting of smelters to inert anodes takes place
		low	1 Mt_annual capacity_/year is retrofitted with inert anodes from 2030 onward. No minimum lifetime of the smelter is assumed to be retrofitted
		high	2 Mt_annual capacity_/year is retrofitted with inert anodes from 2030 onward. No minimum lifetime of the smelter is assumed to be retrofitted
melting furnaces	lifetime	low	20 years mean. Standard distribution with a standard deviation of 1/3 of mean value
		high	30 years mean. Standard distribution with a standard deviation of 1/3 of mean value
	penetration rates of electrically and hydrogen heated furnaces	BAU	no change in furnace types compared to 2020
		electrification	100% of melting capacity inflows are using electrically heated furnaces from 2023
		electrification + hydrogen	Until 2029 same as “electrification”. From 2030, 50% hydrogen and 50% electrically heated furnaces are used

To reduce emissions from secondary melting, replacing natural gas by electricity or (green) hydrogen is the most promising option. Today, hydrogen is not used for high-temperature heating processes on an industrial scale and a TRL of 5 is estimated.^60^ In this study, it was assumed that green hydrogen would become economical by 2030.^61^ Electric melting furnaces were assumed to be already available.^36−38^ In a “high electrification” scenario, electrical heating is used from 2023 for all capacity inflows of melting furnaces (Table 1). In an “electrification + hydrogen” scenario, hydrogen and electrically heated furnaces are used in equal shares from 2030 after solely using electrically heating from 2023 until 2029.

#### Energy and Emissions

We modeled future developments in the specific energy demand of the smelters and melting furnaces as well as changes in the electricity mixes used in both systems (Supporting Information, A.2–3). The future smelter electricity mix was assumed to either remain at the 2020 global average or to improve toward mostly renewable energy sources by 2050, following IAI’s B2DS scenario for 2050.^9^ For the melting furnaces, the global electricity-mix was assumed to follow two scenarios from the IEA:^1^ a more conservative one (Stated Policies) and a more optimistic one (Net Zero Emissions). Indirect emissions were calculated based on the produced aluminum, its specific energy demand, and the carbon intensity of the energy carrier used. Inert anodes were assumed to have zero direct emissions, while PFC emissions in the noninert anode smelters were assumed to decline toward today’s state-of-the-art technologies until 2050 (Supporting Information, A.2–3).

#### GHG Emission Pathways

To integrate the different scenario parameters into relevant storylines that can be used for analyzing emissions scenarios of the aluminum cycle, we defined different scenario pathways. These are explained in Table 2 together with the corresponding parameters used in the scenarios. We used these pathways to quantify the impact of different interventions on annual and cumulative emissions.

**Table 2:**
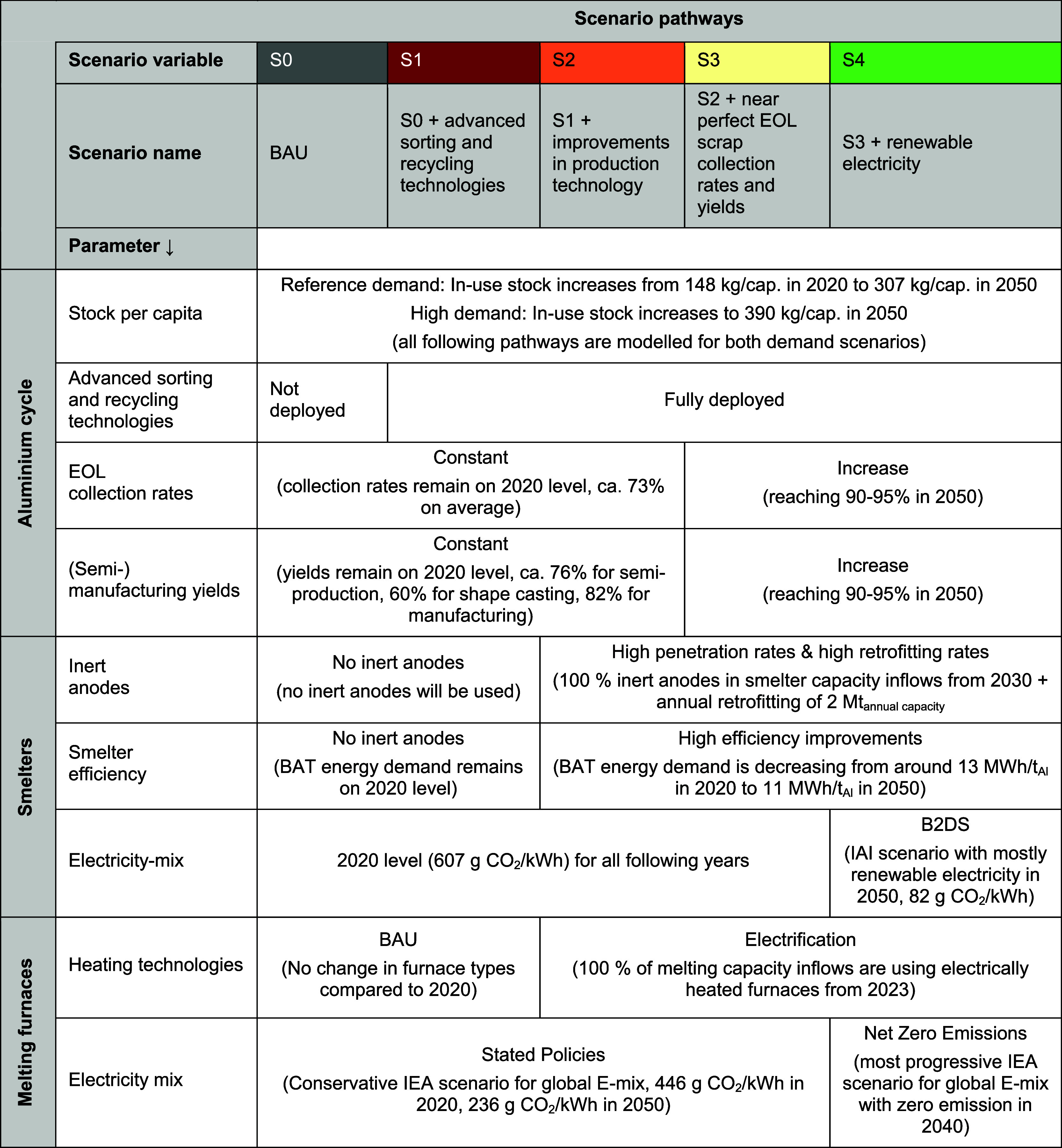
GHG Emission Scenario Pathways and Corresponding Parameter Choices

In order to put the cumulative emissions in context, we assumed carbon budgets for the aluminum sector of 17.9 and 6.4 Gt CO_2_-eq to limit global warming to 2 or 1.5 °C,^62^ respectively, assuming that 1.5% of the total global carbon budget is allocated to the aluminum sector^63^ (see Supporting Information, A.4).

In the BAU scenario (S0), we assumed that not all EOL scrap can be used, future EOL collection rates and yields are constant, no new technologies are implemented, and the electricity-mixes are not (for smelters) or only in a conservative way (global electricity-mix for melting) improved. When sorting and recycling technologies can be improved or incentives are higher to recycle all EOL scrap, less primary aluminum needs to be produced (scenario S1). In scenario S2, new production technologies, such as inert anodes and electrified melting furnaces, are deployed in a progressive way and energy efficiencies increase. In S3, collection rates for postconsumer scrap and manufacturing yields improve to a near perfect level by 2050, in addition to S2. In S4, an electricity-mix mostly based on renewable energy is expected to be reached by 2050.

## Results

### Global Aluminum Cycle

In 2020, the in-use stock was ∼148 kg/cap and 1.2 Gt in total. The demand for final products was ∼86 Mt/a (Figure 2). Approximately 21 Mt/a of 30 Mt/a obsolete products were collected (71%), while most of this was recycled in the refiners to produce casting alloys (15 Mt). There was no scrap surplus and all mixed postconsumer scrap could be absorbed by refiners. In total, ∼80 Mt/a secondary aluminum was produced (including internal refining in the foundries). The rest of the demand was met with primary aluminum (∼66 Mt/a).

In the BAU and reference demand scenario, the in-use stock will reach ∼308 kg/cap in 2050 (Figure 3). The flow of obsolete products will increase to ∼98 Mt/a, and the demand for final products will reach ∼145 Mt/a. Figure 3 shows the scenario for constant EOL collection rates and (semi-) manufacturing yields. This corresponds to a scenario where sorting and recycling technologies are not improving sufficiently to use all EOL scrap, resulting in a scrap surplus; using the same allocation as today, ∼ 53 Mt/a of the ∼98 Mt/a obsolete products would be recycled to casting alloys. Since in 2050, there will only be a demand for ∼34 Mt/a by the refiners for casting alloys, the remaining 19 Mt/a (marked as an orange flow in Figure 3) would need additional separation or sorting to be recycled. If advanced sorting and recycling technologies are deployed, this scrap could be recycled in remelters. When directly replacing primary aluminum, the primary aluminum demand in 2050 could be reduced from ∼94 Mt to ∼75 Mt and secondary aluminum production would increase from ∼153 to ∼172 Mt/a. Sankey diagrams of the quantified aluminum cycle for other scenarios are shown in Supporting Information, B.4.

### Inertia of Industrial Assets

**Figure 4:**
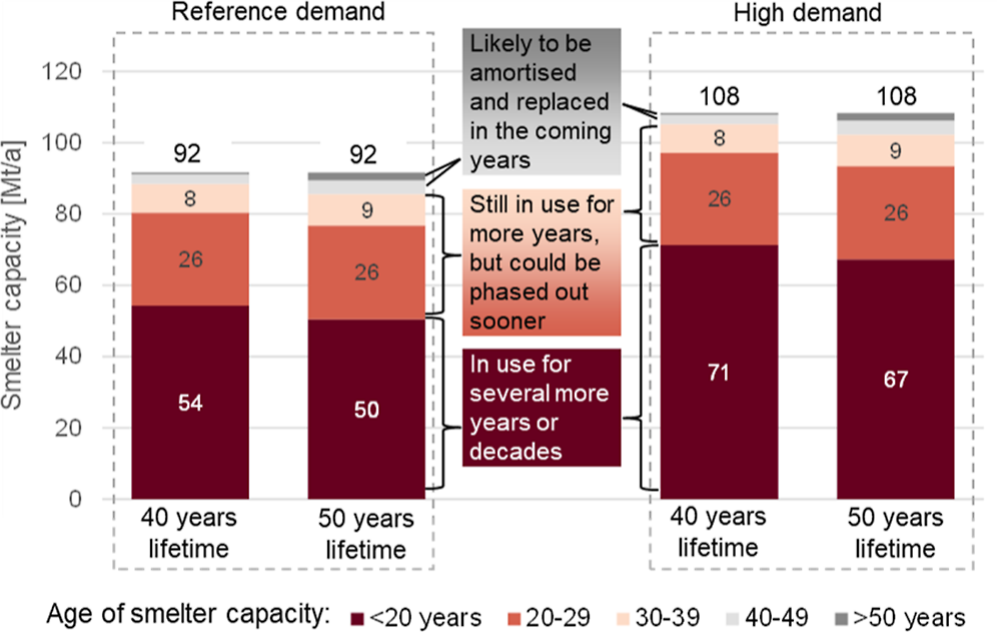
Age distribution of global aluminum smelter capacities in 2030 for reference and high demand, and 40 and 50 years mean lifetime scenarios. Lifetime is assumed to be normally distributed, leading to some smelting capacities being older than the mean lifetime.

Figure 4 presents the cohort composition of industrial assets in different aluminum and asset lifetime scenarios. In 2030, the year in which inert anodes are assumed to be available, between half and two-thirds (51–71 Mt annual capacity) of the smelter capacity will be younger than 20 years and thus likely to remain in use for several more years or even decades before being amortized. Retiring smelters from this age group is economically difficult, resulting in a lock-in effect: retrofitting should be the preferred option to increase inert anode penetration in younger smelters. Less than 10% (<7 Mt annual capacity) of the smelter capacity will be 40 years or older, and thus likely to be replaced in the subsequent years. The influence of the smelter lifetimes on the age distribution is small when comparing 40 and 50 years; the capacity, which is younger than 20 years in 2030 differs by only 4 Mt/a. In comparison, the demand scenario has a higher impact on the age distribution: the younger than 20 years group makes up for 67–71 Mt/a smelter capacity in the high demand scenario compared to 51–55 Mt/a in the reference demand scenario.

### Impact of Assets Lifetimes and Market Penetration Rate on Specific Direct Emissions of the Primary Production

**Figure 5:**
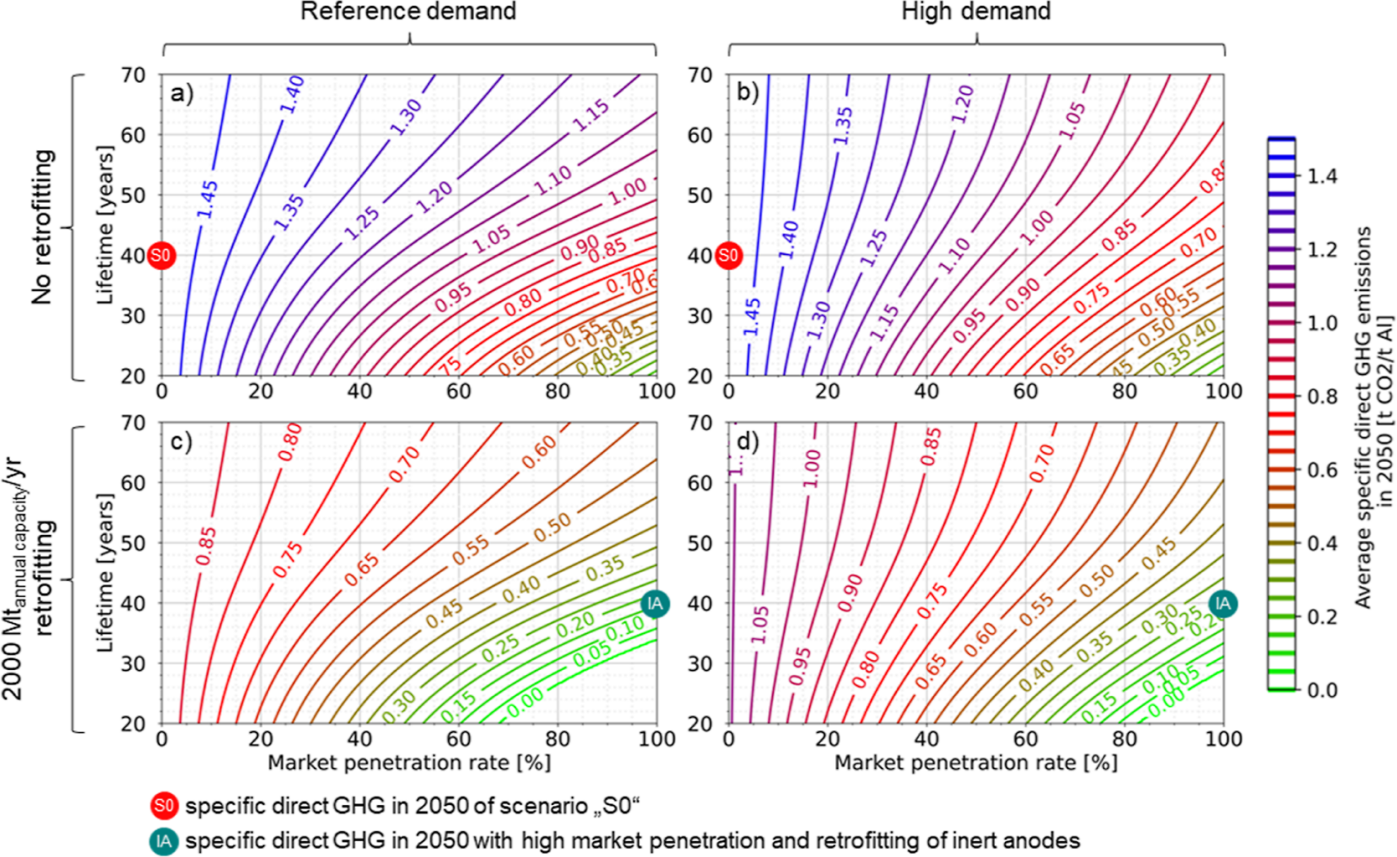
Sensitivity analysis of specific direct emissions of the primary aluminum production in 2050 depending on smelter lifetime and market penetration rate (MPR) of inert anodes. Emissions are shown as contour lines for two demand and retrofitting scenarios, where each line represents one solution for specific direct emissions in 2050. MPR is used as a constant percentage of inert anodes used in new built smelters each year from 2030. Smelter lifetimes are normally distributed and used over the entire model time frame. Here, we show scenarios using the primary aluminum demand without advanced sorting and recycling technologies.

Direct emissions in primary aluminum production are affected by penetration rates of the inert anode technology, the level of retrofitting, the lifetimes of industrial assets, and primary aluminum demand (Figure 5). For a mean smelter lifetime of 40 years, combined with retrofitting and high MPRs of inert anodes from 2030, specific direct emissions can be reduced from ∼2 t_CO_2_-eq._/t_Al_ in 2020 to ∼0.16–0.23 t_CO_2_-eq._/t_Al_ in 2050 (marked with “IA” in Figure 5). However, this requires that from 2030 onward, no new smelters are built using conventional technologies (such as prebake cells) generating direct emissions.

The impact of an increasing MPR becomes smaller with longer lifetimes. Increasing retrofitting (Figure 5c,d) does not change the shape of the contour plots but simply leads to lower specific direct emissions. Reducing direct emissions close to zero by 2050 can only be achieved with substantial retrofitting, a high MPR and shorter smelter lifetimes. Without retrofitting, the potential of a high MPR for reducing the specific direct emissions is slightly higher in the high demand scenario (Figure 5b,d) because inert anodes penetrate the technology stock faster. However, when including substantial annual retrofitting, lower specific emissions are more likely in the reference demand scenario. Here, we show scenarios using the primary aluminum demand without advanced sorting and recycling technologies. The sensitivity analysis and contour plot for scenarios with the advanced sorting and recycling technologies, including “S1” and “S2” can be found in the Supporting Information, B.7.

### GHG Emission Pathways

**Figure 6:**
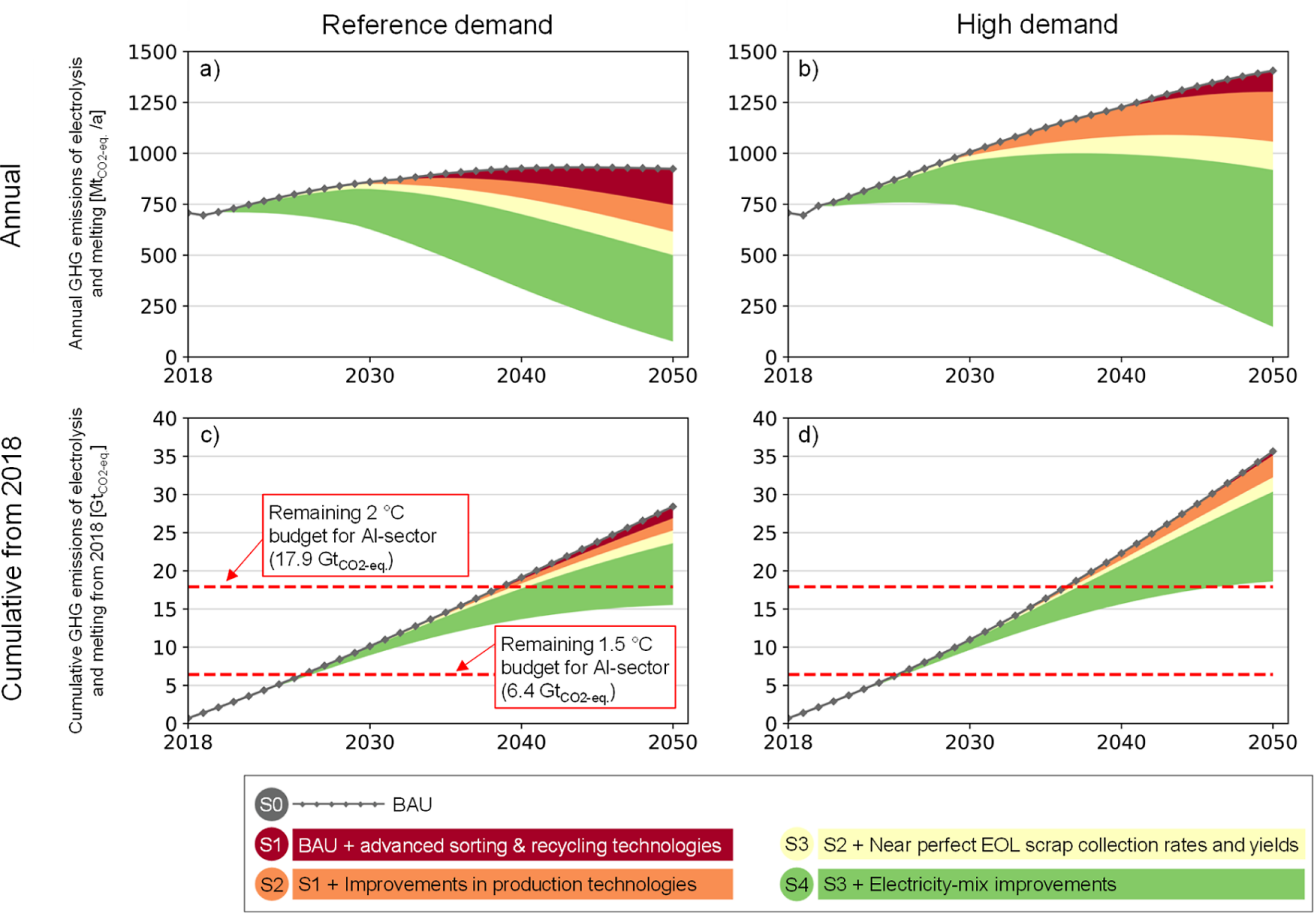
Annual (a and b) and cumulative from 2018 (c and d) GHG emission pathways for smelters and melting furnaces for reference and high demand scenarios, assuming a mean lifetime of 40 years for smelters and 20 years for melting furnaces. GHG emissions are shown in wedge diagrams, where the different pathways are subtracted from a business-as-usual (BAU) pathway and visualized as colored areas beneath to show the reduction potential of each intervention. BAU emissions are shown as a red dotted line on top (S0). Four different wedges are used to show the impact of implementing the following actions on GHG emissions: (S1) deploying advanced sorting and recycling technologies to use all EOL scrap, (S2) implementing technology improvements (inert anodes and electric melting furnaces) to a large extent, (S3) improving EOL scrap collection rates and yields, and (S4) reaching a renewable electricity-mix by 2050. Assumptions on the estimated remaining carbon budgets can be found in Supporting Information, A.4.

While specific emissions are declining for all scenarios, total GHG emissions are harder to abate due to the growing aluminum demand (Figure 6). In the BAU scenario (“S0”), annual emissions would increase from 710 Mt_CO_2_-eq._/a in 2020 to 920 Mt_CO_2_-eq._/a in 2050 in the reference demand scenario (Figure 6a), and up to 1400 Mt_CO_2_-eq._/a in the high demand scenario (Figure 6b). Emissions can be reduced by deploying advanced sorting and recycling technologies to use all available EOL scrap (“S1”), especially in the reference demand scenario where the lower demand for EOL scrap is leading to an earlier scrap surplus. Emission savings by 2050 from technology improvements (“S2”) by implementing inert anodes and electric melting furnaces in a progressive way (see Table 2), together with efficiency improvements, range between 17.5 and 19.4%. Most of the reductions result from implementing inert anodes (12.9–13.8%), followed by increased state-of-the-art energy efficiencies of the smelters (3.5–5.8%) and the implementation of electric melting furnaces (<1%). Emissions can be further reduced when improving EOL collection rates and yields (“S3”). The last wedge shows the reductions due to improvements in the electricity-mix, where mostly renewable energy sources are used by 2050. This intervention has the highest potential to reduce GHG emissions related to electrolysis and melting. Down to 80 and 150 Mt_CO_2_-eq._/a are reached in the reference and high demand scenarios, respectively, when combining all interventions.

All pathways result in cumulative emissions exceeding the 1.5 °C budget of 6.4 Gt_CO_2_-eq._ already before 2030. Cumulative emissions stay within the 2 °C budget for the reference demand scenario when all interventions are implemented, while the high demand scenario is slightly overshooting this budget. The importance of a renewable electricity-mix (S4) is increasing with higher aluminum demand. The difference in cumulative emissions when assuming longer lifetimes for smelters and melting furnaces, 50 and 30 years, respectively, is small. Emission reductions would be ∼0.4 Gt_CO_2_-eq._ smaller in S3 compared to the shorter lifetimes.

## Discussion

### Future Aluminum Production Capacity

Future GHG emissions of the aluminum cycle are determined by the future demand for aluminum—captured by the dynamics of the in-use stocks—and the specific emissions of aluminum production—captured by the dynamics of production capacity stocks. Emissions from an increasing demand can be partially compensated by higher recycling, especially if EOL collection rates are increased. Still, additional primary production and smelter capacity will be needed, especially in the “high demand” scenarios. While the availability of postconsumer scrap will increase, it is unclear if and to what extent it can directly substitute primary aluminum due to the difficulty to meet alloy specifications with a high mixed scrap content. To overcome this challenge, alloy-to-alloy recycling needs to be achieved through design for disassembly and recycling, improved dismantling and sorting, and incentives for recycling.^9,39,40^ The future secondary melting capacity also depends on the evolution of manufacturing yields less manufacturing scrap will require less melting capacity. However, the overall melting capacity will increase due to the growing availability of postconsumer scrap. Higher collection rates will further increase the need for secondary furnaces, as long as it is technically and economically feasible to use this additional scrap.

### New Aluminum Production Technologies and the Inertia of Industrial Assets

New technologies in aluminum production have great potential to reduce emissions. However, the deployment of these technologies is heavily dependent on the dynamics of the existing stocks of production capacity and on their market readiness. For a fast deployment of inert anodes and to avoid lock-ins, it is essential that aluminum smelters can be retrofitted. A similar situation applies to the secondary melting furnaces and the deployment of low-carbon energy sources such as electricity or hydrogen, even if the GHG emissions of secondary production are small compared to primary production. In our model, we focused on inert anodes for smelters, and hydrogen and electrification for melting furnaces, as they are considered to be the most promising technologies.^64^ Other potential technologies for emission reduction are under development, such as carbon capture^58,65^ or carbothermic reduction,^66^ but they are likely to cost more.^58,65,67^

Inert anodes and other emerging technologies are particularly needed to mitigate direct emissions from anode consumption and PFC emissions. Our most ambitious scenarios (S2–S4) rely on high market penetration and retrofitting rates, leading to a share of ∼90% inert anodes in the smelter capacity stock and a substantial decline of specific direct emissions. Alternative scenarios (S0–S1) in which conventional smelting capacities continue to be built after 2030 lead to a lock-in effect, which would need to be compensated by an early retirement of capacities or more retrofitting, making climate targets more difficult and expensive to achieve. Although inert anode cells are claimed to be compatible with existing smelters,^68^ retrofitting options are still unclear in terms of technical feasibility and costs. Finally, a fast penetration of inert anodes does not necessarily lead to an absolute emission reduction: if the change is primarily driven by the need for additional production capacity, as in higher demand scenarios, total emissions increase significantly due to the growing indirect emissions from energy consumption.

Similar conclusions can be drawn for secondary melting furnaces, where fossil fuels need to be replaced by electricity or hydrogen to reduce fuel-related emissions. The assumption that no fossil-fired melting furnaces are to be built in the future is highly ambitious and should be interpreted as an upper boundary for emission reduction. The choice between electricity and hydrogen is highly case-specific: for instance, hydrogen could be favored for melting furnaces with a higher melting capacity and for those using highly contaminated scrap, such as refiners, due to the capacity and feedstock restrictions of electric melting furnaces. Using hydrogen and electricity in melting furnaces will also require adapting infrastructure and processes. Still, the shorter lifetimes of melting furnaces (20–30 years) compared to smelters (40–50 years) allow for a faster replacement of existing furnaces.

### Technology-Explicit GHG Emission Pathways

Our technology-explicit analysis shows that the 1.5 °C carbon budget is by far exceeded in all our scenarios. A 1.5 °C target could only be met by additional measures to those considered in this study, such as carbon capture,^58,65^ an even faster transition to low-carbon electricity sources, or demand reduction. However, aluminum has the potential to reduce emissions in other sectors such as transport or energy^10^ and carbon capture, which is less attractive in the aluminum sector than in the steel or cement sector, for example.^65^ This could call for allocating a higher carbon budget to the aluminum sector. The 2 °C budget, in contrast, is still in reach. However, the aluminum industry needs to become net-zero by 2050 to remain within the 2 °C budget beyond 2050. This is only feasible by implementing all strategies (S4). Otherwise, one will need to rely on carbon capture or negative emissions. Mitigating aluminum production emissions requires combined actions: (1) an improvement of collection and recycling systems to absorb all the available postconsumer scrap, (2) a fast and wide deployment of low-carbon technologies, and (3) a rapid transition of aluminum electrolysis to low-carbon electricity sources. These measures need to be implemented even faster in scenarios with a stronger increase in aluminum demand. This may entail higher production costs as more technologies have to be replaced prematurely before reaching their expected lifetime to overcome lock-ins. Premature retirement of existing assets also takes place due to environmental and health regulations, as for previous Søderberg cells,^69^ or when operating costs exceed the investment costs of new production lines. Operating costs are mainly affected by material and electricity prices and labor costs.^35^ Several production lines, especially in Europe and Northern America, have had to close in recent years due to increasing energy or decreasing aluminum prices.^70^ To increase incentives for low-carbon aluminum production, policy measures such as carbon prices or the support of low-carbon electricity supply can be taken.^35^ This can be complemented by premiums that are already being paid for low carbon aluminum.^71,72^

We introduce technology-explicit MFA as a method to model the transition toward low-carbon industries under different scenarios for demand and technology penetration. Representing production capacities as stocks with lifetimes enables modeling the penetration of new technologies and the replacement of existing ones. This leads to a more realistic assessment of the potential of climate change mitigation strategies. Trade-offs between economic, technical, and environmental aspects can be addressed, such as the need for additional investments due to lifetime reductions, or faster technology penetration but more production and thus emissions in high growth scenarios. We have also shown that the lifetimes of industrial assets underlie a large amount of uncertainty, which can have a significant impact on the penetration speed of emerging technologies and the delay of mitigation strategies. Similarly, emission reduction in the aluminum sector is limited by the future availability of scrap and the capacity of the system to absorb it. For other sectors and material cycles, transitions might be limited by the capacity to develop the capacities needed to mobilize fast enough primary resources.^73^ These factors are currently not well described by the most commonly used models to design future emissions pathways, such as integrated-assessment models.^74,75^ Technology-explicit models of material cycles can fill this gap and support policymakers in designing and evaluating policies to foster low-carbon transitions in the industrial sector.

## Data Availability

Excel and Python files to run the model are available under: 10.5281/zenodo.11090991.

## Supporting Information Available

Detailed description of the methodology of the global aluminum cycle and technology stocks (system definitions, processes and flow descriptions, list of variables, calculation and parameters, assumptions, and projection of key parameters) and additional results (sankeys of global aluminum cycle for different scenarios, stocks of production capacities, future stock shares of inert anodes, direct and indirect GHG emissions from smelters, future stock shares of melting furnaces, GHG emissions from melting, and contour plot for scenarios with advanced sorting and recycling technologies) (PDF)
