# Supplementary material for: Inertia of Technology Stocks: A Technology-Explicit Model for the Transition toward a Low-Carbon Global Aluminum Cycle
Source: Environ Sci Technol. 2024 May 21;58(22):9624–35. doi: 10.1021/acs.est.4c00976 (PMC11155245; doi:10.1021/acs.est.4c00976)
Supplement: Supplementary file 1 — es4c00976_si_001.pdf [file es4c00976_si_001.pdf]

Supporting Information for:

Inertia of technology stocks: A technology-explicit model for the transition towards a low-carbon global aluminium cycle

Authors: Moritz Langhorst[1,*], Romain Guillaume Billy[1], Christian Schwotzer[2], Felix Kaiser[2], Daniel Beat Müller[1]

Affiliations:

1: Industrial Ecology Programme, Department of Energy and Process Engineering, Norwegian University of Science and Technology, Trondheim, 7034, Norway

2: Department for Industrial Furnaces and Heat Engineering, RWTH Aachen University, Aachen, 52064, Germany

*Contact: Moritz.Langhorst@ntnu.no

Summary
Number of pages: 80
Number of figures: 56
Number of tables: 24

Here you can find supplementary information for the detailed methodology of the global aluminium cycle and technology stocks (SI – A) additional results (SI - B).

Table of contents

Supplementary information – A 6

A.1 – Global aluminium cycle 6

System definition 6

Process and flow description 7

List of variables 10

Calculations and parameters 11

Assumptions 22

Projections of key parameters 23

A.2 – Technology layer smelter (electrolysis) 29

System definition and description of flows, stocks and parameters 29

List of system variables 30

Calculations and parameters 30

Assumptions 38

Projections of key parameters 38

A.3 – Technology layer secondary melting 45

System definition and description of flows, stocks and parameters 45

List of system variables 46

Calculations and parameters 47

Assumptions 53

Projections of key parameters 53

A.4 – Carbon budget 59

Supplementary information - B 60

B.1 – Global aluminium cycle – Additional results 60

B.2 – Total stocks of technology (production capacity) 67

B.3 – Stock shares for smelters with inert anodes 68

B.4 – Direct and indirect GHG emission from smelters 70

B.5 – Stock shares for melting furnaces in future 72

B.6 – GHG emissions from melting 74

B.7 – Contour figure of specific direct GHG emissions of the primary aluminium production . 75

References 76

List of figures

Figure S1: Simplified system definition of global aluminium cycle 7
Figure S2: Population data with UN medium variant prospects [10] 13
Figure S3: End-of-life (EOL) scrap collection rates of product categories until 2020 based on [6,17] 22
Figure S4: Parameters for the future development of the global aluminium cycle 23
Figure S5: Potential future per capita stock growth until 2100 adapted from [6] 25
Figure S6: IAI in-use stock scenarios based on [11] 25
Figure S7: Projections used for in-use stock until 2050 26
Figure S8: EOL scrap-collection rates in “increased EOL-collection” 27
Figure S9: Semi-production yields in “increased yields” 28
Figure S10: Manufacturing yields in “increased yields” 29
Figure S11: Simplified system definition of the technology stock system for the smelters 29
Figure S12: Comparison of smelter capacities and primary Al production [11,34,35] 31
Figure S13: Mean utilization rate for smelter based on [11,34,35] 32
Figure S14: Estimated inflow shares of prebake and Søderberg technology based on [37,38,40–42] 33
Figure S15: Description of time-cohort matrix over N periods [43] 34
Figure S16: (Estimated) global average for specific direct GHG emissions based on [4,40,44] 36
Figure S17: Estimated and adjusted BAT energy demand for the global smelters 37
Figure S18: Key parameters for the future development of the global smelter system 39
Figure S19: Market penetration rates for inert anode projections 40
Figure S20: Retrofitting rates for inert anodes projections 41
Figure S21: BAT primary smelter energy demand scenarios 41
Figure S22: Historic global average electricity-mix for smelter and IAI’s B2DS scenario for 2050 [46,58] 42
Figure S23: GHG emission factors for electricity usage in smelter (E-mix projections) 44
Figure S24: Simplified system definition for secondary melting technology stock 46
Figure S25: Historic GHG factors for global electricity [70] 52
Figure S26: Projected energy demand for hydrogen production based on [71] 53
Figure S27: Parameters for future development of the global secondary melting system 54
Figure S28: Market penetration rates for remelters 55
Figure S29: Market penetration rates for refiners 55
Figure S30: Market penetration rates for foundries 56
Figure S31: Development of specific melting energy demand to today’s BAT energy demand in 2050 (based on values in Table 16) 56
Figure S32: GHG emission factors for electricity usage in melting furnaces (E-mix projections)57
Figure S33: Development of total global in-use stock of aluminium under different in-use stock projections 60
Figure S34: Global aluminium cycle in 2020. 60
Figure S35: Global aluminium cycle in 2050 (reference demand, without advanced sorting and recycling technologies, constant, EOL scrap collection rates and yields) (scenario “S0”) 61
Figure S36: Global aluminium cycle in 2050 (reference demand, with advanced sorting and recycling technologies, constant, EOL scrap collection rates and yields) (scenario “S1”) 61

Figure S37: Global aluminium cycle in 2050 (high demand, without advanced sorting and recycling technologies, constant, EOL scrap collection rates and yields) (scenario “S0”) 62
Figure S38: Global aluminium cycle in 2050 (high demand, with advanced sorting and recycling technologies, constant, EOL scrap collection rates and yields) (scenario “S1”) 62
Figure S39: Global aluminium cycle in 2050 (reference demand, with advanced sorting and recycling technologies, increasing EOL scrap collection rates and yields) (scenario “S3”) 63
Figure S40: Global aluminium cycle in 2050 (high demand, with advanced sorting and recycling technologies, increasing EOL scrap collection rates and yields) (scenario “S3”)........................63
Figure S41: Future secondary aluminium production under different demand and parameter scenarios (new and old scrap together) 65
Figure S42: Global aluminium cycle for 2050 (reference demand) with wrought and casting alloy flows. 66
Figure S43: Future primary aluminium production under different demand and parameter scenarios 66
Figure S44: Development of total global aluminium smelter stock (reference demand) 67
Figure S45: Development of total global aluminium smelter stock (high demand) 67
Figure S46: Indirect global GHG emissions of smelters without advanced sorting and recycling technologies (“reference demand”) 70
Figure S47: Indirect global GHG emissions of smelters with advanced sorting and recycling technologies (“reference demand”) 70
Figure S48: Indirect global GHG emissions of smelters without advanced sorting and recycling technologies (“high demand”)..71
Figure S49: Indirect global GHG emissions of smelters with advanced sorting and recycling technologies (“high demand”)..71
Figure S50: Development of melting furnace stock with “high electrification” scenario (100% of melting capacity inflows are using electrically heated furnaces from 2023) for **reference demand** scenario and constant EOL scrap collection rates and yields 72
Figure S51: Development of melting furnace stock with “high electrification” (100% of melting capacity inflows are using electrically heated furnaces from 2023) for **high demand** scenario and constant EOL scrap collection rates and yields..72
Figure S52: Development of melting furnace stock with “electrification + hydrogen” (until 2029 same as “high electrification”. From 2030 50% hydrogen and 50% electrically heated furnaces are used in capacity inflows) for **reference demand** scenario and constant EOL scrap collection rates and yields 73
Figure S53: Development of melting furnace stock with “electrification + hydrogen” (until 2029 same as “high electrification”. From 2030 50% hydrogen and 50% electrically heated furnaces are used in capacity inflows) for **high demand** scenario and constant EOL scrap collection rates and yields 73
Figure S54: Annual global GHG emissions of secondary aluminium melting for different technology projections and furnace lifetimes (reference demand)...74
Figure S55: Annual global GHG emissions of secondary aluminium melting for different technology projections and furnace lifetimes (high demand) 74
Figure S56: Sensitivity analysis of specific direct emissions of the primary aluminium production in 2050 (with advanced sorting and recycling technologies) 75

List of tables

Table S1: Overview of layers in the aluminium cycle system 6
Table S2: Final and obsolete product categories [6] 9
Table S3: List of system variables for the global aluminium cycle 11
Table S4: Lifetime assumptions, stock shares and manufacturing parameters for product categories [5,6] 13
Table S5: Allocation of semi-products to product categories (xj,i) [6] 15
Table S6: Semi-production yield rates and scrap destination [1,6,7,16] 16
Table S7: Metal yields in refiner and remelter 20
Table S8: EOL parameter for product categories [6,7,18] 20
Table S9: List of system variables and sub-variables of the smelter system 30
Table S10: IPCC GHG emission factors of different energy sources for electricity generation (50th percentiles) in $g_{CO2\text{-}eq.}$/kWh [47] 43
Table S11: GHG emission factors for electricity generation by energy source used for smelter electricity-mix 43
Table S12: Overview of the key parameters in the smelter technology system 45
Table S13: List of system variables and sub-variables of the secondary melting system...........47
Table S14: Estimated historic and current inflow shares for types of melting furnaces based on [1,7,16,62,63] 49
Table S15: Key parameters for melting furnaces [4,62–68] 50
Table S16: Mean energy demands for melting furnaces and BAT energy demands 51
Table S17: Mean electrical efficiencies of SOEC electrolyser 53
Table S18: Overview of the key parameters in the smelter technology system 58
Table S19: Primary and secondary aluminium production for reference demand and **no advanced sorting and recycling technologies** 64
Table S20: Primary and secondary aluminium production for reference demand and **advanced sorting and recycling technologies** 64
Table S21: Primary and secondary aluminium production for high demand and **no advanced sorting and recycling technologies** 64
Table S22: Primary and secondary aluminium production for high demand and **advanced sorting and recycling technologies** 65
Table S23: Development of smelter stocks with inert anodes (reference demand, constant EOL scrap collection rates and yields) 68
Table S24: Development of smelter stocks with inert anodes (high demand, constant EOL scrap collection rates and yields) 69

Supplementary information – A

- Detailed methodology of global aluminium cycle (A.1) and technology stocks (A.2-A.3)
    - System definitions
    - Process and flow descriptions
    - List of variables
    - Calculations and parameters
    - Assumptions
    - Projections of key parameters
- Calculations and parameters (more the methods part)

A.1 – Global aluminium cycle

System definition

The system of the global aluminium production is shown in Figure S1. All following descriptions refer to this nomenclature. In this study, the processes of primary production, secondary production (remelter and refiner), casting, production of semi-products and shape castings as well as the manufacturing to final products are included. This is followed by the use phase and end-of-life management. The production system is divided into the wrought alloy production and the casting alloy production to keep track of the different alloy types. Therefore, different layers are used, one for the aluminium mass flows and one for the mass flows of wrought and casting alloys (see Table S1). Note, that only wrought and casting alloys are differentiated in this study and no further distinction into individual alloy groups is made. In a third layer, some of the flows see are further subdivided into different (semi-) product categories. Wrought alloys are processed to semi-products out of primary aluminium and secondary aluminium from the remelter (processes 1, 2, 4, 6). Casting alloys are processed to shape castings in foundries. They are supplied by secondary aluminium from the refiner and by primary Al from the electrolysis (processes 1, 3, 5, 7). The mining and refining processes upstream of the electrolysis are outside of the system boundary of this study. Stocks are included in the in-use stock process (9), whereby stock accumulation in other processes is neglected in this model. Flows between the processes describe the annual mass flow of aluminium from one process to the other. All processes are mass balanced within themselves. The entire system is also mass-balanced within the system boundaries.

Table S1: Overview of layers in the aluminium cycle system

Layer	Included flows
Aluminium-flow layer	All flows in the system definition
Alloy layer (wrought/casting alloys)	All flows in the system definition, except of losses and alumina
Semi-product and product layer	Wrought alloy semis ($F_{6\text{-}8}$), Shape castings ($F_{7\text{-}8}$), Deoxidation aluminium ($F_{5\text{-}8}$), Final products ($F_{8\text{-}9}$), Obsolete products ($F_{9\text{-}10}$)

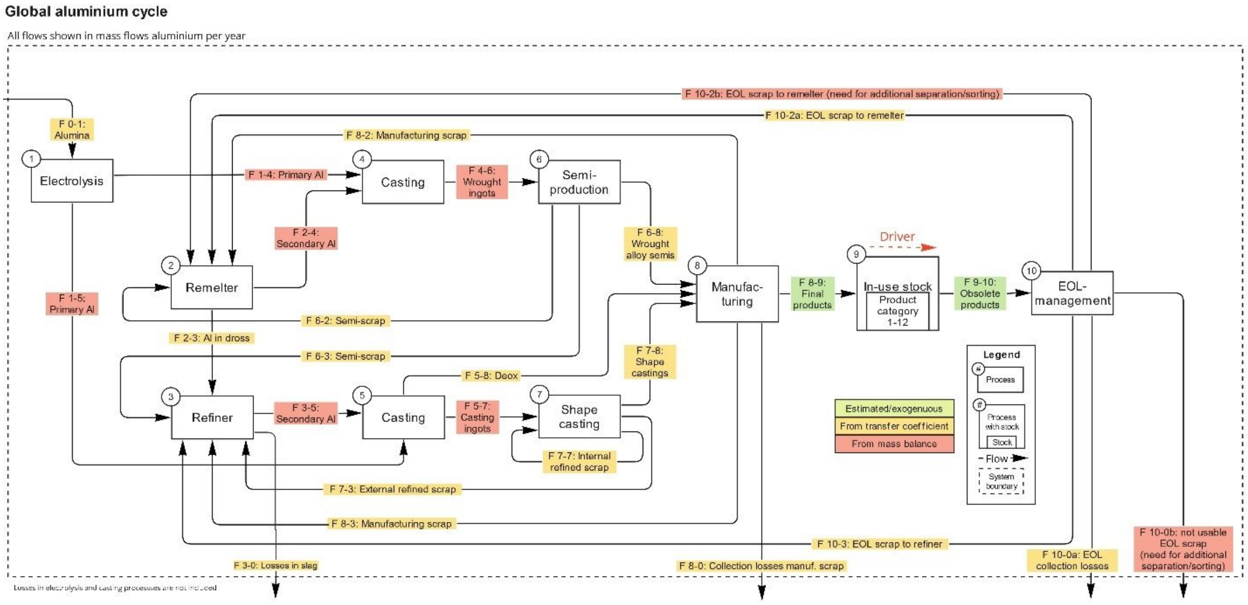

Figure S1: Simplified system definition of global aluminium cycle

Process and flow description

Processes consist of various individual sub-processes, such as different primary production plants or recycling plants, but are aggregated together on a global scale. Also, some flows consist of various individual flows. For instance, the semis-flow ($F_{6\text{-}8}$) represents 7 different flows of wrought alloy semi-products. The same applies to the final products ($F_{8\text{-}9}$) and obsolete products ($F_{9\text{-}10}$) which consist of 12 individual product categories. The flows in Figure S1 are marked in different colours indicating the way of calculation. Processes are numbered from left to right. Flows are labelled according to their origin and the process of destination.

Electrolysis (process 1)

Primary aluminium ($F_{1\text{-}3}$, $F_{1\text{-}4}$) is produced in the electrolysis (process 1). As a simplification, only the metallic aluminium contained in the alumina without any losses is considered for this process as input material. In reality there are about 1 - 2 kg_{Al} or less lost in waste alumina per ton Al produced according to European Aluminium [1]. Billy et al. (2022) [2] report an alumina consumption efficiency of 98,2 % and 99,7 % for two Norwegian smelter lines. Also, there are losses in spent potlining (SPL). Aluminium content in SPL accounts for up to 4 kg_{Al} per ton produced primary aluminium when considering the highest aluminium content (8,5 wt.%) for potlines [3] and upper limit (50 kg) of SPL per ton primary Al [4]. Since only aluminium mass flows are considered in this layer, other input and output materials such as carbon anodes are not included. Carbon dioxide emissions will be considered within the technology layers. The primary aluminium supplies the casting processes of the wrought alloy and casting alloy route.

Casting (processes 4, 5)

The secondary and primary aluminium is cast into wrought and casting ingots ($F_{4\text{-}6}$, $F_{5\text{-}7}$) in the casting processes (4 and 5). Note, that for the sake of simplicity only the term “ingots” is used in this system, although aluminium castings can be called ingot, billet or slab, depending on the size and purpose. Further, it is assumed that the casting for cast alloys (5) is only supplied by primary Al in case that the demand for casting alloys is not met by the secondary Al from refiner. The dilution

or sweetening of the alloyed aluminium with primary aluminium which often takes place in reality [5] is neglected. Also, the casting processes are assumed to work without any losses. Differing from the other semi-products, for the semi product deoxidation aluminium ($F_{5\text{-}8}$) no further processing is needed according to Liu et al. (2013) [6] and thus, this flow is directly entering the manufacturing process. Note also, that for the sake of simplicity primary aluminium is assumed to be transferred as liquid metal to the casthouse. In reality, primary aluminium is often cast into ingots and remelted again together with the secondary aluminium. However, the share remelted primary aluminium is unclear and thus not included.

Remelter and refiner (processes 2, 3)

The remelter process (2) consists of several global distributed remelting plants which use melting furnaces to remelt clean or only light contaminated processing scrap from semi-production and manufacturing as well as clean and separated EOL scrap. During this process dross is generated and recycled in the refiner ($F_{2\text{-}3}$) to recover the contained aluminium.

In the refiner (3), all other scrap is melted and recycled to casting alloys, often under the use of fluxing salt. The molten casting alloys are then casted into ingots in the casting process (5). The generated dross is recycled internally to recover the contained aluminium, whereby some aluminium is lost ($F_{3\text{-}0}$) and remains in non-metallic residues which are used by the cement industry or for mine filling [7].

Semi-production and shape casting (processes 6, 7)

Wrought alloy semi-products ($F_{6\text{-}8}$) are produced out of the wrought alloy ingots ($F_{4\text{-}6}$) in the semi-production process (6). The scrap produced during this process is recycled in the remelter ($F_{6\text{-}2}$) [6]. Collection losses of semi-production scrap are assumed to be neglectable small [6]. Shape castings ($F_{7\text{-}8}$), as the remaining semi-product, are produced in the shape casting process (7), which is also often called foundry. The generated scrap during this process is mainly recycled internally ($F_{7\text{-}7}$) or transported to external refiner plants and recycled there ($F_{7\text{-}3}$).

Manufacturing (process 8)

In the manufacturing process (8) all 9 semi-products are further processed to 12 different product categories. To allocate the semi-products to the final product categories, a constant allocation of the semi-products to the different product categories is used, as shown in Table S5. There are good reasons to question the assumption of an allocation that does not change over time, as especially in electric cars the share of shape castings could decrease compared to internal combustion vehicles. However, quantitative forecasts of the aluminium industry are focussing more on the development of the sectors in general (e.g., transport or packaging) than on how the sectors are composed of semi-products [8], so this assumption is made.

During the manufacturing process processing scarp is generated and recycled either in the remelter ($F_{8\text{-}2}$) or in the refiner ($F_{8\text{-}3}$). Also, some losses during the collection of manufacturing scrap occur ($F_{8\text{-}0}$).

The choice of semi-products as well as the product categories are based on Liu et al. (2013) [6]. The included semi-products are:

- Sheet and plate
- Foil
- Can sheet
- Extrusion
- Wire and cable
- Shape casting
- Other semis (mainly forgings)
- Powder and paste
- Deoxidation aluminium

The 12 considered final product categories as well as product examples are shown in Table S2.

Table S2: Final and obsolete product categories [6]

Abbreviation	Product category	Product examples
BC	Building & construction	Roofing, cladding, window and door frames
TAU	Transportation: Automobiles & light trucks	Engine blocks, suspension components, automobile frames and body panels, wheel rims
TAE	Transportation: Aerospace	Aircraft frames and decking
TOT	Transportation: Others	Railway cars, marine vessels, motorcycles & bicycles
PCA	Packaging: Cans	Beverage cans, aerosol cans
POT	Packaging: Others	Foil for flexible packaging, semi-rigid food containers
ME	Machinery & equipment	Irrigation pipe, ladders, office and hospital equipment
ECA	Electrical: Cables	Wire, cables
EOT	Electrical: Others	Transformers and capacitors, electric lamps
CD	Consumer durables	Air conditioners, refrigerators, dishwashers, cookware
OTN	Other uses: non-destructive use	Other uses except destructive use
OTD	Destructive uses	Metallurgical products for steelmaking

In-use stock (process 9)

The final products ($F_{8\text{-}9}$) enter the in-use stock (9) where they provide a benefit, for instance in form of a car meeting the demand for transport. As soon as the lifespan is over, they leave the in-use stock as obsolete products ($F_{9\text{-}10}$) and enter the EOL-management. The total stock in the in-use process is based on estimations of the in-use stock per capita and historic figures for the global population as well as future projections. The shares of the different product categories in the stock are assumed to stay constant for all years at the values shown in Table S4. The fixed share of product categories is used to be able to follow the stock driven approach and to calculate the system starting from the stock in use. As for the previous assumption of a constant allocation of semi-products to final product categories, this assumption can be questioned, as the demand in individual sectors is predicted to grow differently [8,9]. However, usually the share in the inflows and not in the stock itself is reported in statistics, which complicates the determination of a time varying stock share.

EOL-management (process 10)

EOL-management includes the collection of the obsolete products, the separation and sorting as well as a pre-treatment process before remelting and refining. Since not all obsolete products are collected, losses ($F_{10\text{-}0a}$) occur which also include the pre-melting losses. The EOL scrap is transported to the refiners and recycled there ($F_{10\text{-}3}$) to a large extent but is also recycled in remelters in case of clean and separated EOL scrap ($F_{10\text{-}2a}$). If there is already enough processing scrap from semi-production, shape casting and manufacturing to meet the demand of secondary aluminium for the casting process (5),

If the sum of available EOL scrap and scrap from semi-production, shape casting, and manufacturing exceeds the demand in the casting process (5), the flow of refined EOL scrap ($F_{10\text{-}3}$) is capped so that the demand is just met. In order to recycle the remaining EOL scrap as well, additional separation and sorting is needed to be able to recycle it in the remelters to wrought alloys instead ($F_{10\text{-}2b}$). There it is assumed to replace primary aluminium. When the recycling is not sufficient and the alloys cannot be separated and/or sorted, it is assumed that the scrap leaves the system ($F_{10\text{-}0b}$) and would end up as excess scrap. This procedure is used to show the need for improved sorting and separation technologies. It quantifies the amount of EOL scrap which would be refined in a business as usual development (when using the same allocation of EOL scrap from obsolete product categories to refiner and remelter as today) and EOL scrap that would become excess scrap in the future.

List of variables

All variables, so flows and stock changes, which are used in the system are listed together with the labels used in Table S3. They represent the mass flow of aluminium or aluminium alloy per year. In total, there are 26 flows and, 1 stock and 1 stock change resulting in 28 system variables which need to be determined.

Table S3: List of system variables for the global aluminium cycle

Label of variable	Explanation	Type
$F_{8\text{-}9}$	Total final products	Flow
$F_{9\text{-}10}$	Total obsolete products	Flow
$F_{6\text{-}8}$	Wrought alloy semis to manufacturing	Flow
$F_{8\text{-}2}$	Manufacturing scrap to remelter	Flow
$F_{8\text{-}3}$	Manufacturing scrap to refiner	Flow
$F_{8\text{-}0}$	Scrap losses in manufacturing	Flow
$F_{4\text{-}6}$	Wrought alloy ingots to semi-production	Flow
$F_{6\text{-}2}$	Semi-production scrap to remelter	Flow
$F_{6\text{-}3}$	Semi-production scrap to refiner	Flow
$F_{5\text{-}8}$	Deoxidation aluminium to manufacturing (for steel making)	Flow
$F_{7\text{-}8}$	Shape castings to manufacturing	Flow
$F_{5\text{-}7}$	Casting alloy ingots for shape casting	Flow
$F_{7\text{-}7}$	Internal refined shape casting scrap	Flow
$F_{7\text{-}3}$	External refined shape casting scrap	Flow
$F_{3\text{-}5}$	Secondary Al from refiner (casting alloys)	Flow
$F_{2\text{-}4}$	Secondary Al from remelter (wrought alloys)	Flow
$F_{10\text{-}3}$	EOL scrap to refiner	Flow
$F_{10\text{-}2a}$	EOL scrap to remelter	Flow
$F_{10\text{-}2b}$	EOL scrap to remelter (need for additional separation/sorting)	Flow
$F_{10\text{-}0a}$	Losses in EOL management (collection and pre-melting losses)	Flow
$F_{10\text{-}0b}$	Not usable EOL scrap (need for additional separation/sorting)	Flow
$F_{2\text{-}3}$	Al contained in dross from the remelter which is recovered in the refiner	Flow
$F_{3\text{-}0}$	Losses in refiner	Flow
$F_{1\text{-}4}$	Primary Al for casting of wrought alloys	Flow
$F_{1\text{-}5}$	Primary Al for casting of casting alloys	Flow
$F_{0\text{-}1}$	Aluminium in alumina for electrolysis	Flow
S_9	In-use stock (stock of process 1)	Stock
ΔS_9	Stock change in in-use stock $(S_{1,t} - S_{1,t-1})$	Stock change

Calculations and parameters

The selected time horizon of the global aluminium cycle rages from 1900 to build up the necessary stocks today until 2050. The flows, stocks and stock changes are calculated backwards starting from the in-use stock process.

The necessary equations can be derived from mass balance equations, which are defined for each process as shown in eq. 1 for each year.

$$\sum_i I_{i,t} = \sum_j O_{j,t} + \Delta S_t \quad (1)$$

$\mathrm{I_{i,t}}$	Inflow i of process in year t	$O_{\mathrm{j,t}}$	Outflow j of process in year t
ΔS_t	Stock change of process in year t		

Based on these and on using transfer coefficients and parameters all flows can be quantified.

Starting with the historic and predicted in-use stock per capita, the global population and the estimated stock shares of the specific product categories, the in-use stock of all 12 product categories can be calculated for each year as shown in eq. 2. Note, that the stock shares differ from reported figures on sectoral demand categories because different lifetimes of the product categories can lead to another stock composition than inflow composition. For instance, the average stock shares of packaging products are small compared to other stocks such as the stock of building and construction, although the sectoral demand of building and construction is only about twice as high as the packaging. The reason is, that the packaging products have a significantly shorter lifetime and thus remain only for a short time in the stock. The historic development of the total global in-use stock is shown together with potential future developments used in Figure S33.

Historic population data and the medium variant prospect until 2050 are taken from the United Nations (UN) [10] (Figure S2). The historic in-use stock per capita is derived from IAI data [11]. Future in-use stock per capita is predicted in two different developments, which are explained in more detail at the end of this section. The stock shares per product category are assumed to stay constant, as previously explained, and are shown in Table S4.

$$\mathrm{S_{i,t}} = \mathrm{s_t} * P_t * x_{stock,i} \quad (2)$$

$\mathrm{S_{i,t}}$	In-use stock of product category i in year t [kt]
$\mathrm{s_t}$	In-use stock per capita in year t [kg/capita]
P_t	Population in year t
$x_{stock,i}$	Stock share of product category i of total stock

Table S4: Lifetime assumptions, stock shares and manufacturing parameters for product categories [5,6]

Product category	Mean lifetime	Standard deviation lifetime	Stock share ($x_{stock,i}$)	Manufacturing yield ($\eta_{Manuf,i}$)	Collection rate manuf. scrap ($CR_{Manuf,i}$)	Manuf. scrap to remelter ($x_{Manuf-rem,i}$)	Manuf. scrap to refiner ($x_{Manuf-ref,i}$)
BC	50	15	33,01%	90%	98%	85,5%	14,5%
TAU	20	6	16,40%	84%	98%	29,7%	70,3%
TAE	40	12	0,64%	60%	98%	90,0%	10,0%
TOT	30	9	11,04%	80%	98%	29,7%	70,3%
PCA	1	-	0,54%	75%	98%	90,0%	10,0%
POT	1	-	0,32%	75%	98%	90,0%	10,0%
ME	40	12	10,29%	75%	98%	69,9%	30,1%
ECA	40	12	14,68%	90%	98%	90,0%	10,0%
EOT	20	6	2,68%	80%	98%	71,3%	28,7%
CD	12	3,6	4,50%	80%	98%	73,6%	26,4%
OTN	20	6	5,79%	80%	98%	75,6%	24,4%
OTD	1	-	0,11%	80%	98%	0,0%	100,0%

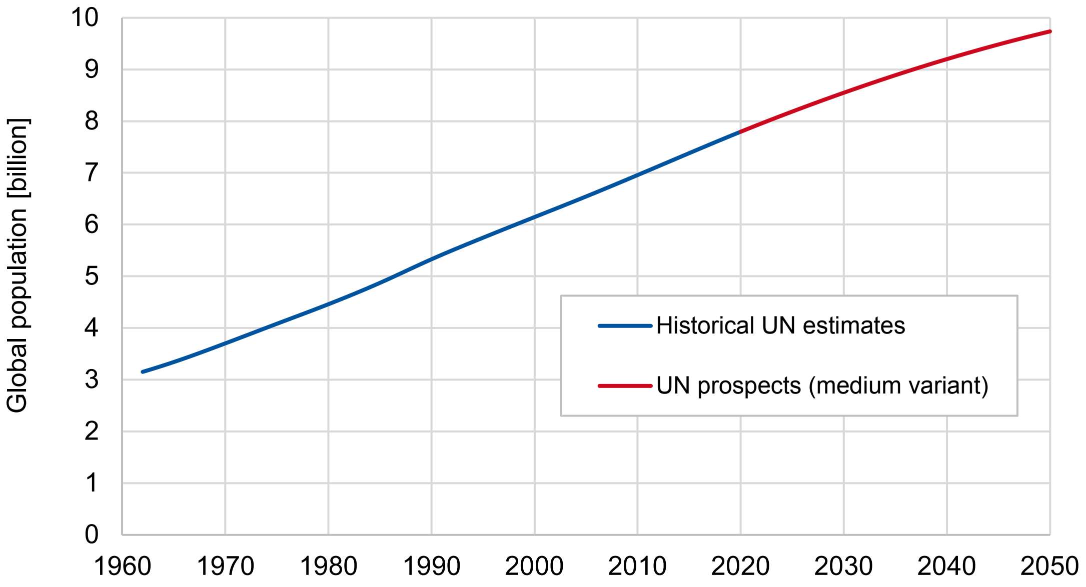

Figure S2: Population data with UN medium variant prospects [10]

Together with the annual stock change and the estimated lifetime, the inflows of new products and the outflows of obsolete products can be calculated, according to equations 3 - 6. For the lifetime, a mean value and standard deviations as shown in Table S4 are used assuming a normal distribution. In order to include the lifetime distribution in the calculations, a python script based on the ODYM model framework [12] is used.

The normal distributed lifetime approach of this python model is based on a probability function, which derives the probability that an input of a certain year is transferred into an output in another year based on the mean value and the standard deviation of the lifetime [13,14]. By this, the outflows of each product category and the resulting inflows are calculated using a normal distribution of

the lifetime. A constant lifetime and no standard deviation were assumed for packaging products and destructive use because of a lifetime of one year, which is the smallest timestep in this mode. Their practical lifetime would be even shorter, such as for beverage cans which could be as short as 60 days [15].

$$\Delta \mathrm{S}_{\mathrm{i,t}} = \mathrm{S}_{\mathrm{i,\,t}} - S_{i,\,t-1} \tag{3}$$

$\Delta \mathrm{S}_{\mathrm{i,t}}$	Stock change of product category i in year t

$$\Delta \mathrm{S}_{\mathrm{t}} = \sum_{i} \Delta \mathrm{S}_{\mathrm{i,t}} \tag{4}$$

$\Delta \mathrm{S}_{\mathrm{t}}$	Total stock change in-use stock in year t

Equation 5 shows how the outflows are calculated based on a lifetime distribution, which describes the probability that an inflow from previous period would leave the stock as an outflow in year t.

$$O_{i,t} \; = I(i,t) \cdot \frac{1}{\sigma\sqrt{2\pi}} e^{-\frac{(t-c-\tau)^2}{2\sigma^2}} \tag{5}$$

$O_{i,t}$	Outflow of obsolete product of product category i in year t [kt]
$I_{i,t}$	Inflow of new final products of product category i in year t [kt]
σ_i	Mean lifetime of product category i [years]
τ_i	Standard deviation of lifetime of product category i [years]

$$I_{i,t} \; = \Delta \mathrm{S}_{\mathrm{i,t}} + O_{i,t} \tag{6}$$

Summing up all product categories leads to the total inflow of new products (F_{8-9}) and the total outflow of obsolete products (F_{9-10}) for each year.

$$F_{8-9,\,t} = \sum_{i} I_{i,t} \tag{7}$$

$$F_{9-10,\,t} = \sum_{i} O_{i,t} \tag{8}$$

To derive the demand for the semi-products, the allocation of the semi-products to the final product categories, shown in Table S5, is used. For instance, machinery and equipment products need 38 % sheet and plate, 39 % extrusion and 22 % casting products as semi-products.

$$D_{Semi\,j,t} = \sum_i (I_{i,t} * x_{j,i} * \frac{1}{\eta_{Manuf,i}}) \qquad 9$$

$D_{Semi\,j,t}$	Demand for semi-product j in year t [kt]
$x_{i,j}$	Share of semi-product j in product category i
$\eta_{Manuf,i}$	Manufacturing yield of product category i
$I_{i,t}$	Inflow of new final products of product category i in year t [kt]

The shape casting flow ($F_{7\text{-}8}$) as well as the deoxidation aluminium flow ($F_{5\text{-}8}$) can be calculated immediately using eq. 9 because they are not aggregated together. The wrought alloy semi-products ($F_{6\text{-}8}$) can be calculated by adding the remaining 7 semi-products.

Table S5: Allocation of semi-products to product categories ($x_{j,i}$) [6]

Product category	Sheet &Plate	Foil	Sheet Can	Extrusion	Wire & Cable	Shape Casting	Deoxidation	Other	Powder & Paste
BC	18%	-	-	72%	-	5%	-	5%	-
TAU	15%	2%	-	12%	-	67%	-	4%	-
TAE	50%	-	-	50%	-	-	-	-	-
TOT	15%	2%	-	12%	-	67%	-	4%	-
PCA	-	-	100%	-	-	-	-	-	-
POT	-	100%	-	-	-	-	-	-	-
ME	38%	-	-	39%	-	22%	-	-	-
ECA	-	-	-	-	100%	-	-	-	-
EOT	33%	-	-	25%	22%	21%	-	-	-
CD	45%	19%	-	17%	-	18%	-	-	-
OTN	17%	-	-	18%	-	16%	-	-	49%
OTD	-	-	-	-	-	-	100%	-	-

The manufacturing scrap per product category can be calculated as:

$$Scrap_{Manuf,i,\,t} = \frac{I_{i,t} * (1 - \eta_{Manuf,i})}{\eta_{Manuf,i}} \qquad 10$$

$Scrap_{Manuf,i,\,t}$	Manufacturing scrap of product category i [kt]
$I_{i,t}$	Inflow of new final products of product category i in year t [kt] (outflow of manufacturing process)

The manufacturing scrap is allocated to the remelter and refiner based on the values in Table S4 whereby a collection rate of 98 % is used [6] and 2 % are lost. All manufacturing scrap from casting alloys (shape castings and deoxidation Al) is allocated to the refiner. Of the wrought alloys (all other semi-products), 90 % are allocated to the remelter, 10 % to the refiner which is based on [5].

Using this, the manufacturing scrap which is refined, remelted or lost can be calculated.

$$F_{8-2,t} = \sum_i Scrap_{Manuf,i,\,t} * x_{Manuf-rem,i} * CR_{Manuf,i,t} \quad 11$$

$F_{8-2,t}$	Remelted manufacturing scrap in year t [kt]
$x_{Manuf-rem,i}$	Share of remelted manuf. scrap of product i
$CR_{Manuf,i,t}$	Collection rate of product category i in year t

$$F_{8-3.t} = \sum_i Scrap_{Manuf,i,\,t} * x_{Manuf-ref,i} * CR_{Manuf,i,t} \quad 12$$

$F_{8-3.t}$	Refined manufacturing scrap in year t [kt]
$x_{Manuf-ref,i}$	Share of refined manufacturing scrap

$$F_{8-0.t} = \sum_i Scrap_{Manuf,i,\,t} * (1 - CR_{Manuf,i,t}) \quad 13$$

$F_{8-0,t}$	Lost manufacturing scrap [kt]

Similar, the amount of scrap generated during the semi-production and shape casting can be calculated as shown in eq. 14 using the semi-production yields from Table S6.

Table S6: Semi-production yield rates and scrap destination [1,6,7,16]

**Semi-product**	**Semi-production yield (η_j)**	**To remelter ($x_{Semi-rem,j}$)**	**To internal refining ($x_{Semi-ref,int,j}$)**	**To external refining ($x_{Semi-ref,ext,j}$)**
Sheet and Plate	72,5 %	100 %	-	-
Foil	78,9 %	100 %	-	-
Can Sheet	72,0 %	100 %	-	-
Extrusion	76,6 %	100 %	-	-
Wire and Cable	76,6 %	100 %	-	-
Shape Casting	60,0 %	-	95 %	5 %
Deoxidation Al	100 %	-	-	-
Other	76,6 %	100 %	-	-
Semis "	100 %	-	-	-

$$Scrap_{Semi-prod,j,t} = \frac{D_{Semi\,j,t} * (1 - \eta_{Semi,j})}{\eta_{Semi,j}} \quad 14$$

$Scrap_{Semi-prod,j,t}$	Semi-manufacturing scrap of semi-product j in year t
$\eta_{Semi,j}$	Yield of semi-manufacturing of semi-product j
$D_{Semi\,j,t}$	Demand for semi-product j in year t [kt]

Thus, the respective amounts of remelted scrap and refined scrap can be derived using the shares of scrap destination of Table S6.

$$F_{6-3,t} = \sum_j Scrap_{Semi-prod,j,t} * x_{Semi-ref,ext,j} \quad 15$$

$Scrap_{Semi-prod,j,t}$	Semi-production scrap of semi j (without shape casting and deoxidation) in year t [kt]
$x_{Semi-ref,ext,j}$	Share of external refined scrap of semi-product j

$$F_{6-2,t} = \sum_j Scrap_{Semi-prod,j,t} * x_{Semi-rem,j} \quad 16$$

$Scrap_{Semi-prod,j,t}$	Semi-production scrap of semi j (without shape casting and deoxidation) in year t [kt]
$x_{Semi-rem,j}$	Share of remelted scrap of semi-product j

$$F_{7-3,t} = \frac{D_{Shape\ castings,t} * (1 - \eta_{Semi,\ Shape\ casting})}{\eta_{Semi,\ Shape\ casting}} * x_{Semi-ref,ext,shape\ casting} \quad 17$$

$F_{7-3,t}$	External refined shape casting scrap in year t [kt]
$D_{Shape\ castings,t}$	Demand for shape castings in year t [kt]
$\eta_{Semi,\ Shape\ casting}$	Yield of semi-manufacturing of shape castings
$x_{Semi-ref,ext,shape\ casting}$	Share of external refined shape casting scrap

Assuming no losses in the collection of semi-production scrap, the demand for wrought alloy ingots (or billets or slabs) can be calculated through mass-balancing the semi-production process.

$$F_{4-6,t} = F_{6-8,t} + F_{6-3,t} + F_{6-2,t} \quad 18$$

Similar, the demand for casting alloy ingots can be calculated mass-balancing the shape casting process and adding the output of shape castings and the external refined scrap.

$$F_{5-7,t} = \mathrm{F}_{7-8,\mathrm{t}} + \mathrm{F}_{7-3,\mathrm{t}} \quad (19)$$

The end-of-life scrap is either remelted, refined, not usable due to additional need for separation and sorting or lost during the collection. Lost scrap would be disposed or incinerated whereby the not usable scrap remains available for recycling.

The directly remelted scrap is calculated as shown in eq. 20. The assumed EOL scrap collection rates are based on Liu et al. (2013) [6] and estimations from the IAI [17], as shown in Figure S3. Future collection rates are assumed to stay either constant or increase in different projections (see later in this section)

$$F_{10-2a,t} = \sum_{i} O_{i,t} * CR_{EOL,i,t} * x_{EOL\,scrap,rem,\,i} * \eta_{pre-melt,i} \quad (20)$$

$CR_{EOL,\,i,t}$	EOL collection rate of product category i
$x_{EOL\,scrap,rem,\,i}$	Share of remelted EOL scrap of product category i
$\eta_{pre-melt,i}$	Pre-melt processing yield of product category i

In order to derive the amount of EOL scrap, which is allocated to the refiner, the remaining demand for aluminium in the refiner needs to be derived. This has to be done in an iterative way due to the circulating flows. In a first step, the allocation rates from Table S8 are used to define the amount of EOL scrap to refining when the allocation of EOL to remelter and refiner stays at today's level (marked with "BAU" for business as usual).

If the demand in the casting process (5) after the refiner (equal to the sum of F_{5-7} and F_{5-8}) is higher than the entering scrap flows (F_{10-3}, F_{2-3}, F_{6-3}, F_{7-3}, F_{8-3}) into the refiner, the flow of EOL scrap to refiner (F_{10-3}) is not changed and remains at the BAU value. The remaining metal demand for the casting process is then met by primary aluminium. If "EOL scrap to refining" exceeds a value so that there would be more inflows in the refiner than secondary aluminium is necessary (F_{3-5}) to meet the casting alloy demand, the "EOL scrap to refining" flow is capped.

Therefore, an "adjusted" flow of EOL scrap to refining is calculated (eq. 22), which is based on the actual demand for secondary aluminium produced in the refiners. If the "adjusted" flow is below the "BAU" value, it shows that not the entire EOL scrap, which would be allocated with the today's allocation, can go to the refiner. In this case, only the "adjusted" value is allocated to the refiner, otherwise the "BAU" value. The metal yields for refiner and remelter are listed in Table S7 (eq. 23).

$$Scrap_{EOL,\,ref,\,BAU,t} = \sum_{i} O_{i,t} * CR_{EOL,i,t} * x_{EOL\,scrap,ref,\,i} * \eta_{pre-melt,i} \quad (21)$$

$Scrap_{EOL,\,ref,\,BAU,t}$	EOL scrap to refiner with same allocation as today (BAU) [kt]
$x_{EOL\,scrap,ref,\,i}$	Today's share of remelted EOL scrap of product category i

$$Scrap_{EOL,ref,adj,t} = \frac{F_{5-7} + F_{5-8}}{\eta_{ref}} - [F_{6-3,t} + \mathrm{F}_{2-3,t} + \mathrm{F}_{8-3,\mathrm{t}} + \mathrm{F}_{7-3,\mathrm{t}}] \qquad 22$$

η_{ref}	Metal recovery rate in refiner
$Scrap_{EOL,ref,adj,t}$	"Adjusted" EOL scrap going to refiner [kt]
F_{2-3}	Al in dross (unknown)

$$F_{10-3,t} = \begin{cases} Scrap_{EOL,ref,adj,t} & , \quad Scrap_{EOL,ref,adj,t} < Scrap_{EOL,ref,BAU,t} \\ Scrap_{EOL,ref,BAU,t} & , \quad Scrap_{EOL,ref,adj,t} \geq Scrap_{EOL,ref,BAU,t} \end{cases} \qquad 23$$

$F_{10-3,t}$ *EOL scrap to refiner in year t [kt]*

In case there is EOL scrap with an additional need for separation and sorting, meaning that the "adjusted" flow of EOL scrap to refining is used, this scrap either goes to the remelter (in case of successful separation) ($F_{10\text{-}2b}$) or is not used and leaving the system ($F_{10\text{-}0b}$). To model this, the "$\text{scrap}_{\text{add. separation}}$" flow from eq. 24 is used to define the amount of scrap with need for additional separation. If advanced sorting and recycling processes are developed so all EOL scrap can be used, the EOL scrap is allocated to the remelter (eq. 25), if not it is leaving the system (eq. 26).

$$Scrap_{add.\ separation,t} = \begin{cases} Scrap_{EOL,ref,BAU,t} - Scrap_{EOL,ref,adj,t} & , \quad Scrap_{EOL,ref,adj,t} < Scrap_{EOL,ref,BAU,t} \\ 0 & , \quad Scrap_{EOL,ref,adj,t} \geq Scrap_{EOL,ref,BAU,t} \end{cases} \qquad 24$$

$Scrap_{additional\ separation,t}$ EOL scrap with need for additional separation in year t [kt]

$$F_{10-2b} = \begin{cases} Scrap_{add.\ separation,t} & , \quad \textit{with advanced sorting and recycling} \\ 0 & , \quad \textit{without advanced sorting and recycling} \end{cases} \qquad 25$$

F_{10-2b} EOL scrap to remelter (need for additional separation or sorting)

$$F_{10-0b} = \begin{cases} 0 & , \quad \textit{with advanced sorting and recycling} \\ Scrap_{add.\ separation,t} & , \quad \textit{without advanced sorting and recycling} \end{cases} \qquad 26$$

F_{10-0b} EOL scrap not usable (need for additional separation or sorting)

Table S7: Metal yields in refiner and remelter

Secondary melting plant	Metal yield	Reference/comment
Remelter	95 %	Derived from [7]
Refiner	98 %	Derived from [7]

Table S8: EOL parameter for product categories [6,7,18]

Product category	Pre-melt processing yield ($\eta_{pre-melt,i}$)	EOL-scrap to remelter ($x_{EOL\ scrap,rem,\ i}$)	EOL-scrap to refiner (todays value, excluding reallocation to scrap surplus) ($x_{EOL\ scrap,ref,\ i}$)
BC	100%	49%	51%
TAU	97%	5%	95%
TAE	100%	49%	51%
TOT	100%	49%	51%
PCA	99%	80%	20%
POT	97%	-	100%
ME	97%	9%	91%
ECA	97%	-	100%
EOT	97%	-	100%
CD	97%	-	100%
OTN	97%	-	100%
OTD	-	-	-

As a last input material in the refiner, the flow of aluminium in dross, coming from the remelter, ($F_{2\text{-}3}$) needs to be derived. Without advanced sorting and recycling technologies, meaning some EOL scrap remains unused, the aluminium in dross can be calculated based on the inflows and the metal yield of the remelter. With advanced sorting and recycling and thus usable EOL scrap, it depends on several other flows as shown in eq. 27 and 28.

$$F_{2-3} = \begin{cases} \dfrac{A * (1-\eta_{Rem})}{\eta_{Rem}}, & with\ advanced\ sorting\ and\ recycling \\ (F_{10-2a} + F_{8-2} + F_{6-2}) * (1-\eta_{Rem}), & without\ advanced\ sorting\ and\ recycling \end{cases} \quad (27)$$

η_{Rem} Metal yield in remelter

A Auxiliary value

$$A = F_{10-2a} + F_{8-2} + F_{6-2} + Scrap_{EOL,\,ref,\,BAU,t} - \left[\frac{F_{5-7,t} + F_{5-8,t}}{\eta_{ref}} - [F_{6-3,t} + F_{8-3,t} + F_{7-3,t}]\right] \quad (28)$$

Losses in EOL-management occur due to the collection losses and pre-melt treatment losses, which is calculated in eq. 29.

$$F_{10-0a,t} = \sum_i O_{i,t} * (1 - CR_{i,t}) * (1 - \eta_{pre-melt,i}) \quad (29)$$

Mass-balancing the remelter and refiner processes leads to the respective flows of secondary aluminium. It is assumed that all scrap flows are reused in the same year.

$$F_{2-4,t} = F_{8-2,t} + F_{10-2a,t} + F_{10-2b,t} + F_{6-2,t} - F_{2-3,t} \quad (30)$$

$$F_{3-5,t} = F_{6-3,t} + F_{2-3,t} + F_{7-3,t} + F_{8-3,t} + F_{10-3,t} - F_{3-0,t} \quad (31)$$

The demand for primary aluminium can be derived by mass-balance based on the missing amount of aluminium to meet the demand in the casting processes. If there is enough secondary aluminium from the refiner, no primary aluminium is used in the casting alloy casting process (5).

$$F_{1-4,t} = F_{4-6,t} - F_{2-4,t} \quad (32)$$

$$F_{1-5,t} = \begin{cases} F_{5-7,t} + F_{5-8,t} - F_{3-5,t}, & F_{3-5,t} < F_{5-7,t} + F_{5-8,t} \\ 0, & F_{3-5,t} \geq F_{5-7,t} + F_{5-8,t} \end{cases} \quad (33)$$

The total primary demand can be calculated by adding up both primary flows. Further, this is assumed to be equal to the aluminium content in the alumina (F_{0-1}) when neglecting aluminium losses in the electrolysis.

$$F_{0-1,t} = D_{Primary\ total,\ i} = F_{1-4,t} + F_{1-5,t} \quad (34)$$

$D_{Primary\ total,\ i}$	Total demand for primary Al [kt]

Data for EOL scrap collection rates is taken from Liu et al. (2013) [6] for values until the year 2009. For the year 2020 data was derived from IAI data [17]. In between linear interpolation was used. This is shown in Figure S3. Significant differences for some product categories, such as packaging others (POT) or consumer durables (CD), for 2009 and 2020 values lead to a sharp increase between both years. Also, the collection rate for transport aerospace is slightly decreasing between 2009 and 2020 through these assumptions. However, this assumption was made to include the most recent values for collection rates for 2020.

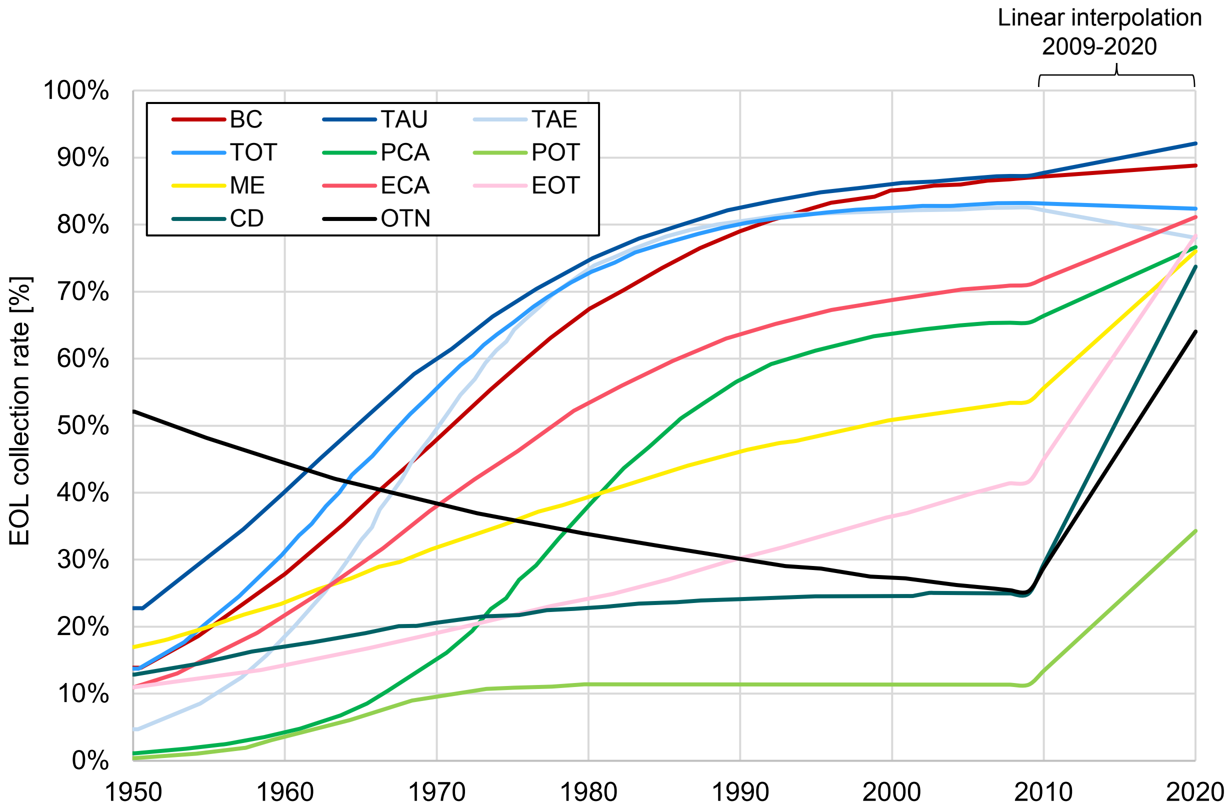

Figure S3: End-of-life (EOL) scrap collection rates of product categories until 2020 based on [6,17]

Assumptions

For a better overview of all assumptions made in order to model and calculate the global aluminium cycle, these are listed in the following:

1. The in-use stock per capita, as the main driver of the aluminium demand, is taken for historical values from IAI's global aluminium cycle [11] and is based on different projections for future development assuming that the stock reaches a saturation level at one point.
2. The composition of the different product categories in the stock is assumed to be constant for all years, also for future years. In future, the demand could shift away from casting alloys due to less demand in the transport sector (e.g., less engine blocks) leading to even more scrap with an additional need for separation/sorting (or new demands for casting alloys are needed).
3. The allocation of semi-products to the final product categories is constant over time.
4. Estimated product lifetimes are taken from Liu et al. (2013) [6] and are assumed to be normal distributed. Product categories with a lifetime of only one year are assumed to have a constant lifetime.
5. Aluminium losses in primary production are neglected, as the alumina consumption efficiency is high (>98 %) [2]
6. No dilution or sweetening with primary aluminium is included in the casting alloy casting (process 5). Primary aluminium is only allocated to the casting alloy casting for mass-balancing if the scrap inputs are too low to meet the demand.
7. Losses in both casting processes are neglected.
8. Collection losses of semi-production scrap are neglected.

9. Losses during internal refining in the foundries and shape casting are neglected.
10. Aluminium losses in the remelter are all allocated as dross to the refiner where the dross is melted using flux salt to recover the containing aluminium and other metals.
11. Metal yield and recovery rate for remelter and refiner are constant over time and for all alloys/products.
12. Manufacturing and semi-production yields are assumed constant for historic values. Future values depend on the used parameter projection.
13. EOL scrap collection rates are based on two different references [6,17] and interpolated between 2009 and 2020. Future values depend on the used parameter projection.
14. EOL scrap is recycled in the remelter to wrought alloys if advanced sorting and recycling technologies are implemented.
15. No time delays for the processing of aluminium flows are considered apart from the products in the in-use process. This means that for instance scrap flows are recycled instantaneous in the same year or that products entering the in-use process are all produced in the same year.

Projections of key parameters

In order to analyse how the global aluminium cycle may develop in the next decades until 2050, potential projections for different parameters are used. The key parameters studied are the demand of aluminium products, i.e., the stock per capita, the development of advanced sorting and recycling technologies, the EOL scrap collection rate as well as the semi-production and manufacturing yields. This is illustrated in Figure S4. For the in-use stock, projections until 2100 are used to take its long-term trend into account.

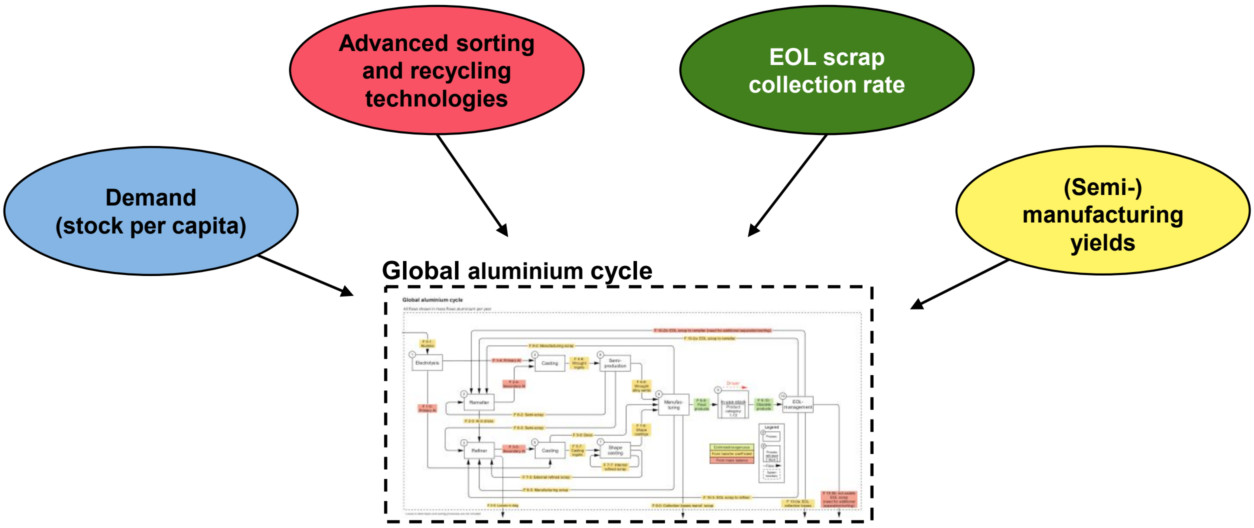

Figure S4: Parameters for the future development of the global aluminium cycle

Stock per capita

Since the stock per capita is the main driver of the global aluminium demand in this study, its historic and future development has a crucial role. Factors influencing what kind of stock growth occurs are for instance the economic development of developing countries and in which speed

their stock per capita is equalising with developed countries. Often, estimations of the stock growth are linked to the development of the gross domestic product (GDP) [19,20]. Furthermore, the development of new products out of aluminium and changed compositions of the actual stock can lead to a higher stock in the future. An example would be an increased substitution of steel products in vehicles by light-weight aluminium components.

In a first step, the historic development is estimated based on IAI data. For the future stock development, different projections are used to represent potential growth patterns. The basis for the historic stock per capita for the years 1962 until 2019 is taken from IAI's global aluminium cycle [11]. In addition to this, a regression analysis as applied to the per capita stock data in order to achieve a smooth development without major deviations in individual years and to represent the long term development. To build up the stock before 1962, an exponential growth starting in the year 1900 is assumed.

In order to predict future stock growth, a Gompertz model as presented in Liu at al. (2013) [6] and shown in eq. 35 was used. This is used to continue at the 2019 stock level with the same growth rate. Furthermore, the potential long-term development of the stock is taken into account by using saturation levels and a saturation times. In the saturation year, about 98 % of the saturation level is reached. The equation is solved numerical (e.g., in Excel) for alpha and beta under the constraints of keeping the growth rate in year 2019 at the same level as in the historical data and reaching 98 % of the saturation level in the respective saturation year.

$$s_{\mathrm{t}} = \frac{s_{sat.}}{1 + \left(\frac{s_{sat}}{s_0} - 1\right) * \exp\left(\alpha * \left(1 - \exp\left(\beta * t\right)\right)\right)} \tag{35}$$

s_{t}	In-use stock per capita in year t [kg/capita]
$s_{sat.}$	Saturation level [kg/capita]
s_o	In-use stock in year 0 (2019)
α, β	Parameter

Potential projections for future stock growth are shown in Figure S5 with the first legend value indicating the saturation level in kg per capita and the second value indicating the saturation year. As saturation levels 300, 350, 400 and 600 kg were used together with the years 2075 and 2100 as saturation years. Different saturation levels and times were adapted from [6], where 600 kg per capita are used in a high scenario, 400 kg in a medium scenario, and 200 kg per capita in a low scenario together with 2075 and 2100 as saturation years. In contrast to saturation levels in [6], the 200 kg saturation level is not considered further at this point, as it seems unlikely in view of today's global or European and North American average. Instead, a 300 kg saturation level used as the lowest saturation level in Figure S5. Also, a 350 kg saturation level for 2075 is added. The in-use stock per capita in 2019 according to IAI [11] was at around 425 kg for North America, at around 278 kg and at around 189 kg for China [11], with a global average of 142 kg. The main assumption for future stock development is here, that developing countries and regions with lower stock per capita equalise in the future gradually with developed countries such as North America or Europe.

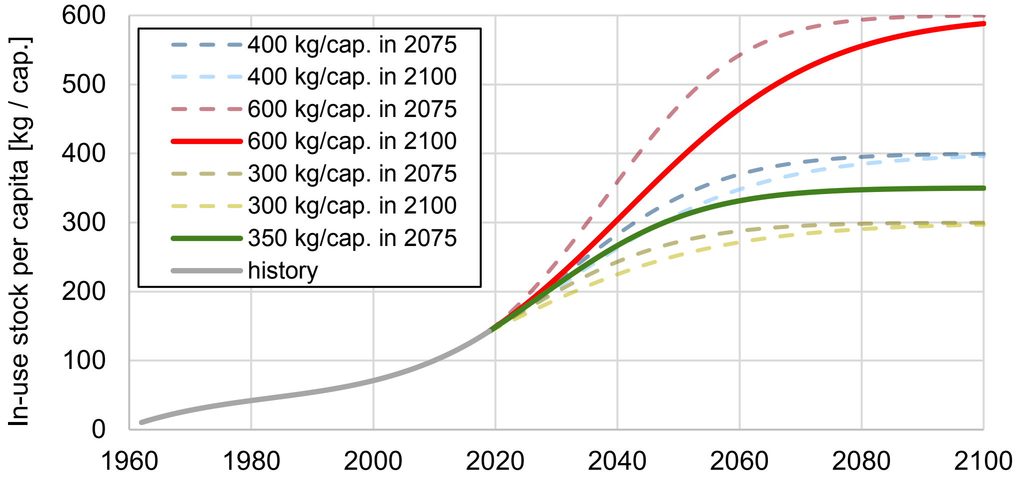

Figure S5: Potential future per capita stock growth until 2100 adapted from [6]

In IAI's global aluminium cycle [11], potential future stock growth scenarios are published as well. The IAI differentiates between a reference, a high demand scenario and a low and high substitution scenario. The reference and high demand scenarios, which are shown in Figure S6, are used further in this study as an upper and lower boundary of the per capita stock. The average global per capita stock reaches 285 kg and 380 kg in 2050 in the IAI's reference and high demand scenarios respectively.

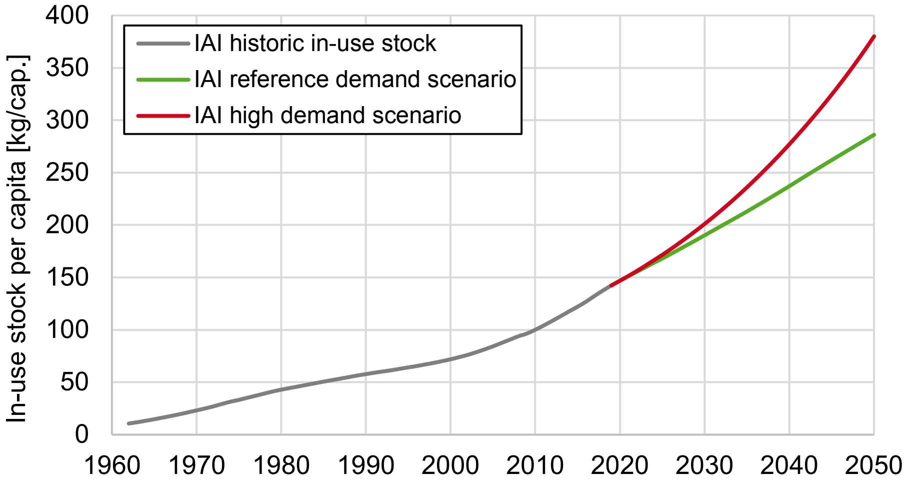

Figure S6: IAI in-use stock scenarios based on [11]

The IAI scenarios themselves are not used in this model because the assumptions behind these scenarios are unknown. Furthermore, modelling a stock growth using the Gompertz function is a common method used in literature [6,19–21] and the assumptions in terms of saturation level and time are transparent.

However, the per capita stock (kg/cap) projections used in this study are aligned with the IAI's scenarios, which are used as a reference point here. The "600 kg/cap in 2100" stock growth from Figure S5 (continuous red curve) reaches 390 kg/cap in 2050 and thus is similar to the high demand scenario from the IAI (380 kg/cap). Thus, the "600 kg/cap in 2100" stock growth is used as a high demand projection in this study. The main difference between both developments is

that the IAI is predicting a more exponential growth in their high demand scenario (Figure S6). The “350 kg/cap in 2075” stock growth (marked in continuous red) is the closest one of the projections in Figure S6 to the IAI reference scenario in terms of 2050 values (the IAI scenarios reach until 2050). Thus, this stock growth is chosen as a reference projection in this study. However, the reference projection used here differs from the IAI reference scenario both in terms of the stock level in 2050 and the shape of the stock growth until 2050. The IAI reference scenario is following a linear trend until 2050, while the reference projection used in this study already reach its turning point in year 2028 with a decreasing growth rate afterwards. This would lead to a decreasing total stock growth, and thus a decrease of the demand for new products, which does not correspond to the IAI reference demand scenario. For the reference demand used here, about 307 kg per capita are reached in 2050. The development for both until 2050 is shown in Figure S7.

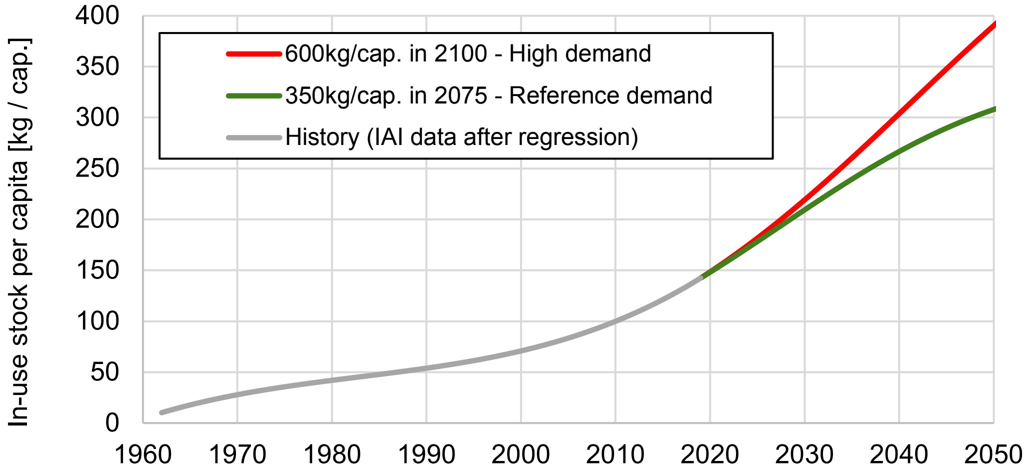

Figure S7: Projections used for in-use stock until 2050

Development of advanced sorting and recycling technologies

The second parameter is the question if advanced sorting and recycling technologies can be developed or not.

When the availability of post-consumer scrap increases to the point that it exceeds the demand for casting alloys, a surplus of EOL scrap becomes likely [22–24]. If advanced sorting and recycling processes are developed or new applications are found for mixed scrap [25,26], this scrap could also be used in the remelters. For this purpose, we use the parameter “advanced sorting and recycling technologies”. In scenarios where the advanced technologies are deployed, we assume that there are no quality constraints for recycling of post-consumer scrap in the remelters.

Since wrought alloys account for the major part in new and obsolete products, a recycling into wrought alloys again would be feasible. Examples of technology improvements could be eddy current separation or the usage of robotics, deposit schemes and smart direct recycling systems to improve the quality of the sorted scrap [27]. Also, alloy-to-alloy recycling, so keeping extruded, rolled and cast aluminium apart, smart dismantling technologies, an improved scrap preparation and advanced sorting systems to avoid mixing alloys could lead to a higher quality of the recycled

scrap. Especially the alloy-to alloy recycling is mentioned to avoid the cast sink, so the recycling of mixed wrought alloys to casting alloys [27]. The alloy-to-alloy recycling is especially done in manufacturing scrap recycling where alloys can be separated better compared to post-consumer scrap. An example for alloy-to-alloy recycling is given by Novelis and Volvo [28]. They implemented a closed-loop recycling where the manufacturing scrap is recycled in a closed loop to the same products so the demand for primary aluminium is claimed to be lowered. Covanta [29] aims to separate non-ferrous metals from municipal solid waste by using induction sorting to air-separate different alloys. Further, limiting the number of alloys in products could lead to easier separation of alloys in the scrap [5]. Also, increased shares of electric vehicles and their lower demand for casting alloys compared to internal combustion vehicles is lowering the demand for future casting alloys. The development of new shape-cast product applications could counteract this [5]. Since the price difference between secondary and primary aluminium is relatively small (about 10 - 15 %), often primary aluminium is used for wrought alloys and post-consumer scrap is downcycled into casting alloys. An increased price difference due to the future excess scrap could lead to higher incentives to improve separation and sorting technologies and to make the EOL-scrap usable for wrought alloys [30].

End-of-life scrap collection rate

Increased collection rates of EOL-scrap can lower the demand for primary aluminium and thus the total energy demand significantly and are another important parameter in the global aluminium cycle. As potential projections in this study, the future collection rates either not increase further after 2020 and remain at this level (see Figure S3) or they increase gradually to 95 % (for building and construction as well as transport products) and 90 % (all remaining product categories) in 2050. The "increasing" projection is based on the "near perfect collection" scenario conducted by Liu et al. (2013) [31], whereby the authors mention that this scenario is very optimistic and could be difficult to achieve for products such as aluminium foil. However, this scenario is used to point out the impact of increased collection rates for the total material and energy demand and the resulting emissions of the global aluminium cycle. The development of the collection rates in the increased EOL-collection projection, which is based on the Gompertz model as for the in-use stock growth, is shown in Figure S8.

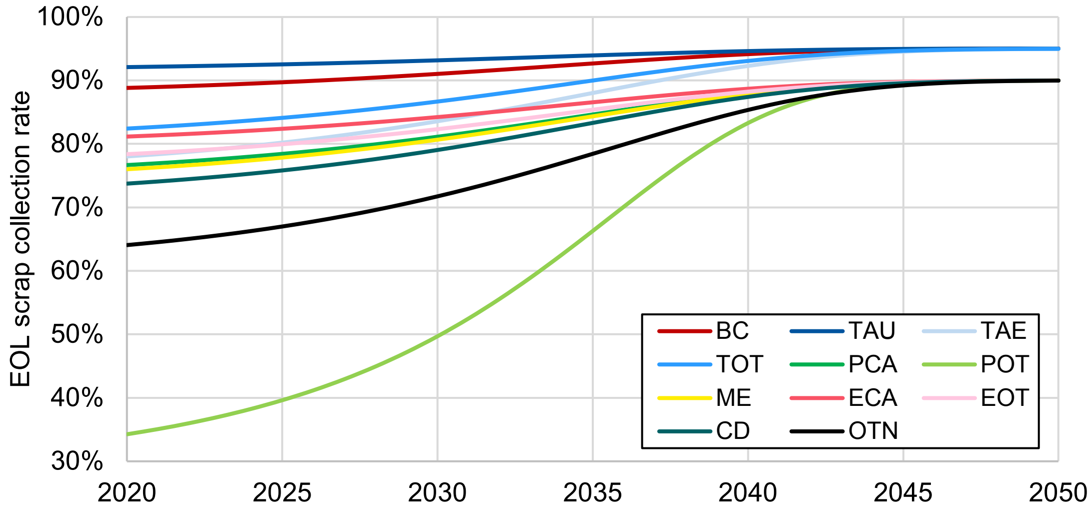

Figure S8: EOL scrap-collection rates in "increased EOL-collection"

Semi-production and manufacturing yield

To consider potential material efficiency improvements in the semi-production and manufacturing processes the respective yields are chosen as a fourth parameter. Yield is defined as the metal output relative to all inputs of a process. As described for the EOL collection rates, the processing yields remain constant in one projection and gradually increase in another. Manufacturing yields are thereby assumed to increase from 2023 onwards to 95 % and semi-production yields to increase to 90 % in 2050, based on the "technologies for yield improvement" scenario conducted by Liu et al. (2013) [31]. Improvements in the material efficiency could be achieved by near-net shape casting [25] or smart-connected processes [32]. The assumed development of both yields is shown in Figure S9 and Figure S10 for the respective semi-products and final product categories.

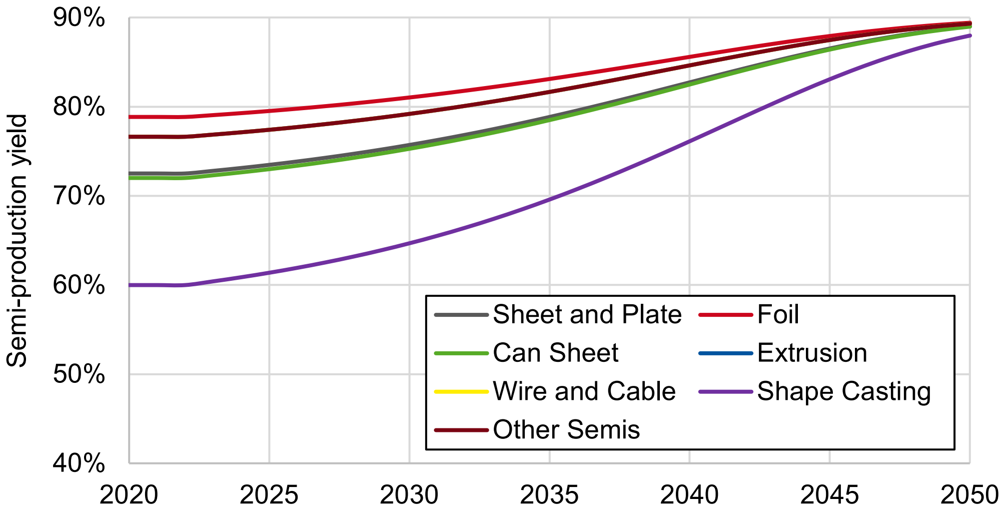

Figure S9: Semi-production yields in "increased yields"

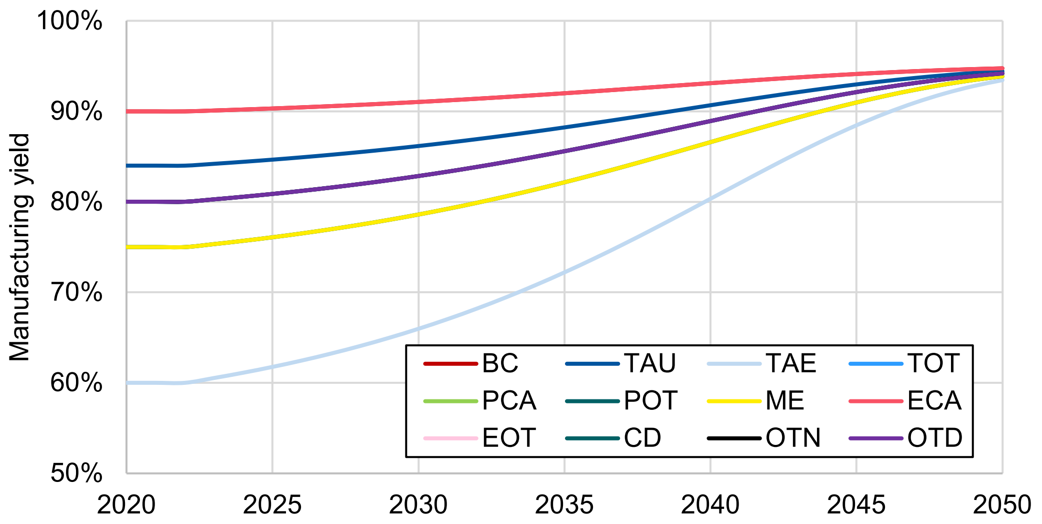

Figure S10: Manufacturing yields in "increased yields"

A.2 – Technology layer smelter (electrolysis)

System definition and description of flows, stocks and parameters

The stock-driven MFA system for technology stocks in primary production is shown in Figure S11. The system consists of the technology stock of smelters as a process aggregating the global production capacity of all smelters, new capacity entering the stock ($F_{0\text{-}T1}$) and obsolete capacity leaving the system ($F_{T1\text{-}0}$). The technology stock or production capacity is what drives the inflows of new production capacity. The production capacity itself is driven by the previously derived primary production. A utilization rate of the smelters is used since there is more production capacity than actual production (Figure S13.).

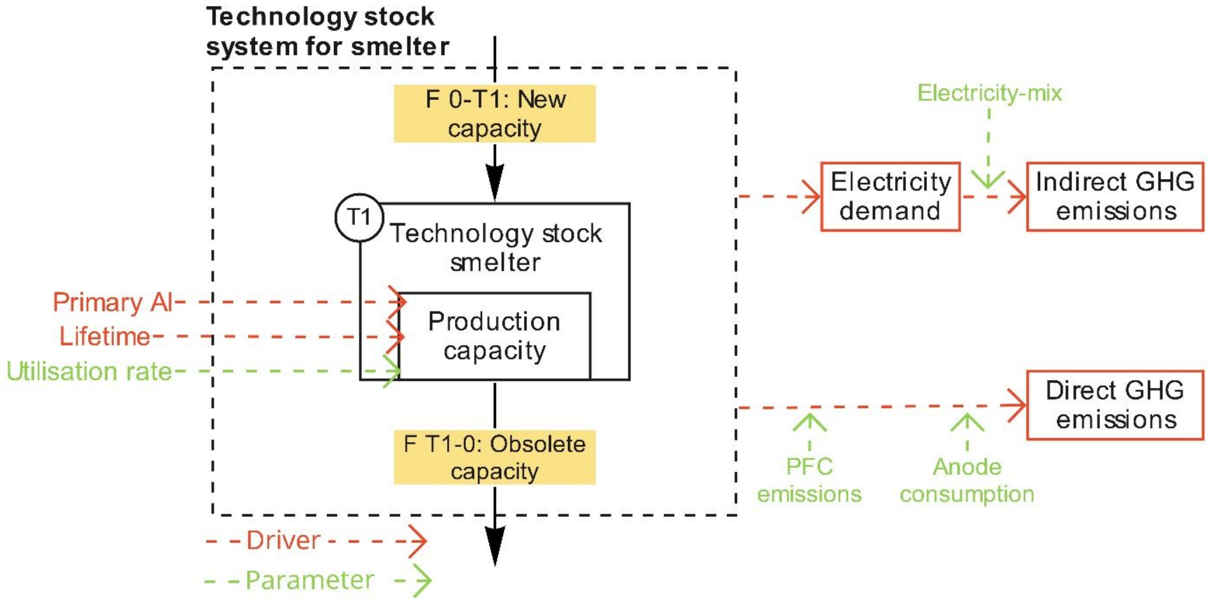

Figure S11: Simplified system definition of the technology stock system for the smelters

To determine the outflows of capacity or obsolete capacity, a lifetime approach is used as explained previously for the obsolete aluminium products. The inflow of new smelter capacity is leaving the stock as obsolete capacity when its lifetime is over (following a lifetime distribution). Capacity inflows can vary between prebake or Søderberg electrolytic cells and inert anode cells as a potential future technology.

The mass flow of primary produced aluminium multiplied by the specific energy leads to the total energy demand for primary production. The resulting GHG emissions are then calculated based on the direct emissions emitted by the smelter and the indirect emissions, which are based on the electricity demand and the energy carrier used for the electricity generation.

List of system variables

The system variables are listed in Table S9 together with sub-variables used for further calculation. In total, there are two main flows and one stock representing the total smelter capacity. These consist of ancillary variables which represent the flows and stock of the specific technology, namely prebake, Søderberg and inert anode electrolysis cells.

Table S9: List of system variables and sub-variables of the smelter system

Label of system variable/ sub-variable	Explanation	Type
$\mathbf{F_{0\text{-}T1}}$	**Total inflow of new smelter capacity**	**Flow**
$F_{0\text{-}T1,\ PB}$	Inflow of new prebake capacity	Flow
$F_{0\text{-}T1,\ SS}$	Inflow of new Søderberg capacity	Flow
$F_{0\text{-}T1,\ IA}$	Inflow of new inert anode capacity	Flow
$\mathbf{F_{T1\text{-}0}}$	**Total obsolete smelter capacity**	**Flow**
$F_{T1\text{-}0,\ PB}$	Outflow of obsolete prebake capacity	Flow
$F_{T1\text{-}0,\ SS}$	Outflow of obsolete Søderberg capacity	Flow
$\mathbf{S_{T1}}$	**Total stock of smelter capacity**	**Stock**
$S_{T1,\ PB}$	Stock of prebake smelter capacity	Stock
$S_{T1,\ SS}$	Stock of Søderberg smelter capacity	Stock
$S_{T1,\ IA}$	Stock of inert anode smelter capacity	Stock
$\mathbf{\Delta S_{T1}}$	**Total stock change of smelter capacity**	**Stock change**

Calculations and parameters

The first step to determine the smelter system is to calculate the total smelter capacity or the total stock of capacity (eq. 36). The main driver of the production capacity stock is the primary production from the global aluminium cycle.

The production capacity is defined as the potential annual primary aluminium production of the global aluminium smelters. The actual throughput of the smelters, the annual primary aluminium production, would be below the capacity. Companies, such as Alcoa [33] often publish their production capacity as “nameplate capacity”, which is estimated based on design capacity and

normal operating efficiencies and thus does not necessarily represent the maximum possible production. To take this into account, an average utilization rate of the smelters is used. The utilization rate is defined as the percentage of actual primary production in relation to total capacity. The utilization rate varies significantly depending on the sources used for both the global smelter capacity as well as the actual global primary aluminium production. To estimate the utilization rate, the annual primary production data from IAI [11] and U.S. Geological Survey (USGS) [34] are used. For the global smelter capacity, data from USGS [34] for the years 1994 until 2021 and from Pawlek (2022) [35] for the years 2012 until 2021 are used. In both data, temporary idled and standby capacity is included, but not permanently shut down capacity. Pawlek [35] reports that data from China is often imprecise and varies on the sources, which could lead to differences in both datasets. The different datasets for primary production and smelter capacity are shown in Figure S12. Based on a combination of the two capacity data sets and two primary production data sets, four (two for 1994 until 2011) utilization rates are calculated. The utilization rate varies widely on the sources and lays somewhere between 73 % and 89 % for the year 2021. To calculate the total stock, a mean value of these together with a regression curve (3rd degree polynomial) is used for the period between 1994 and 2021. Historic utilization rates are assumed to be at the level of 1994 (87 %). Future utilization rates are assumed to increase linearly again to 90 % in the year 2030. This is shown in Figure S13. All smelter technologies are assumed to have the same utilization rate.

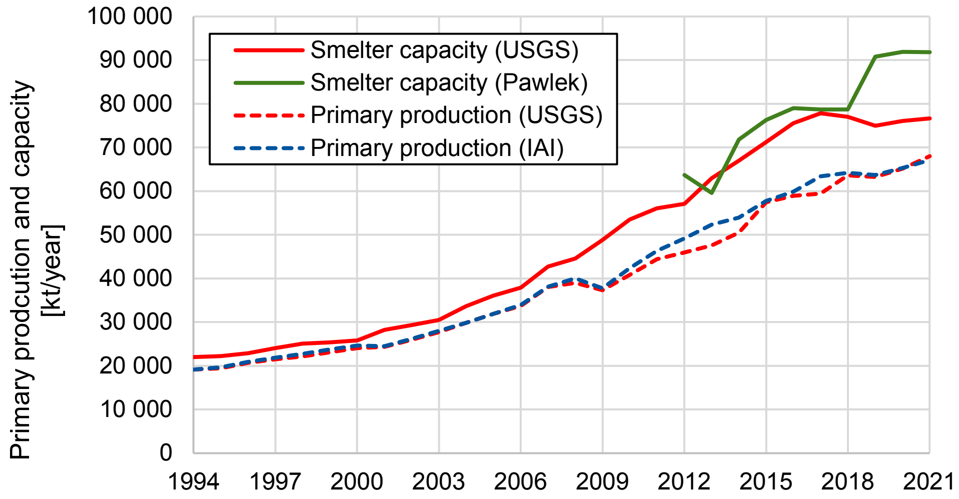

Figure S12: Comparison of smelter capacities and primary Al production [11,34,35]

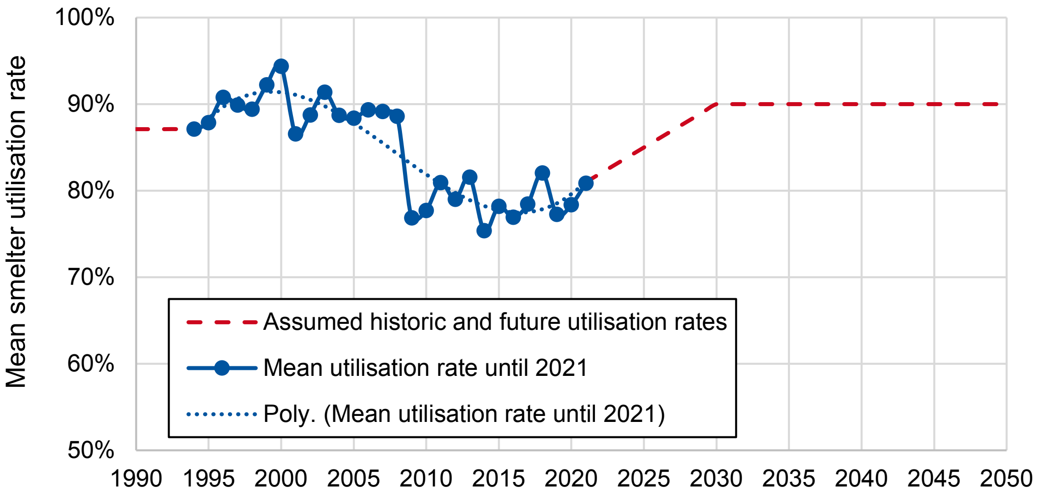

Figure S13: Mean utilization rate for smelter based on [11,34,35]

$$S_{T1,\,t} = \frac{Primary\ Al_t}{UR_t} = \frac{F_{1-4,\,t} + F_{1-5,t}}{UR_t} \qquad (36)$$

$S_{T1,\,t}$ Total smelter capacity stock in year t$F_{11-5,\,t}$Primary Al for casting of wrought alloys in year t (from Al cycle layer) [kt]

$F_{11-6,\,t}$ Primary Al for casting of casting alloys in year t (from Al cycle layer) [kt]

UR_t Average utilization rate of all smelters in year t

The total inflows and outflows of smelter capacity ($F_{0\text{-}T1}$, $F_{T1\text{-}0}$) are derived in the same way as for the in- and outflows of the in-use process of the aluminium cycle system using a lifetime distribution and a python script based on the ODYM model framework [12].

The in- and outflows are based on the stock development and annual stock change and a mean lifetime of the smelter. The terminology of lifetime can have various meanings in this case. Especially a technical and economic lifetime needs to be differentiated. For instance, the electrolysis cell can become unusable if important components break down and can no longer be repaired. On the other side, economic reasons can lead to closed or shutdown capacity when the smelter is not operating economical anymore due to older technology compared to other smelters. Also, political reasons might play a role. For instance, excess capacity in one country could constrain investments in another country [36]. Furthermore, retrofitting of existing capacity could extend the lifetime of the electrolysis cell. In this study, however, no distinction is made between the actual cause of retirement of the smelters. Instead, the lifetime is determined empirically from statistical data. For the mean smelter lifetime, 40 and 50 years with a standard deviation of 13 and 17 years (a third of the lifetime) respectively are assumed in two different projections.

New capacity can consist of the construction of new production plants or by heavy retrofitting of existing capacity. Thereby, the type of technology can vary between prebake and Søderberg electrolytic cells as well as inert anode cells as a potential future technology. The distinction

between new inflows and retrofitting is not always clear. For instance, when production capacity of a smelter is increased significantly by an increased amperage, no physical capacity would be added but it would be considered as an inflow of new capacity. Also, when a different technology is used (e.g., prebake instead of Søderberg or inert anodes) it would usually be considered as a new inflow. On the other hand, retrofitting would be done on existing capacity without changing the technology itself and more improving its efficiency. In this model, new inflows are used for all added production capacity due to increased demand or replacing retired smelters. Retrofitting does not affect the capacity but only the technology and thus the resulting emissions in case of the inert anodes. In contrast to the previously description of retrofitting, this is not explicitly considered as a new inflow in this model but would technically count as such.

In order to take the different technologies of prebake and Søderberg smelters in the stock into account, the inflow shares of the respective technology for the past are estimated. Until the year 1930, the prebake technology was the dominant technology [37]. In the following years the Søderberg technology became the more and more dominant technology [37]. An exponential growth of the Søderberg technology in the inflowing capacity is assumed from 0 % in 1930 to 90 % in 1939. It is reported that China started to build their first prebake smelter in 1975, which became the major technology from 1994 onwards, and closed all Søderberg technology by 2005 [38]. The last Søderberg smelter in America opened in 2007 in Brazil [39]. The IAI [40] reports a share of prebake technology for the global primary production of 63 % for 1990, increasing to around 95 % in 2019. To achieve a similar stock share for 2019 in this model, it is assumed that the inflow share of prebake technology increased exponentially from 10 % in 1964 to 98 % in 1994. It is assumed that the inflow share of Søderberg smelters remains at 2 % due to investments of for instance Russia in eco-Søderberg smelter [41,42]. The development of the estimated inflow shares of the respective technology is shown in Figure S14, whereby the actual development would not be steady.

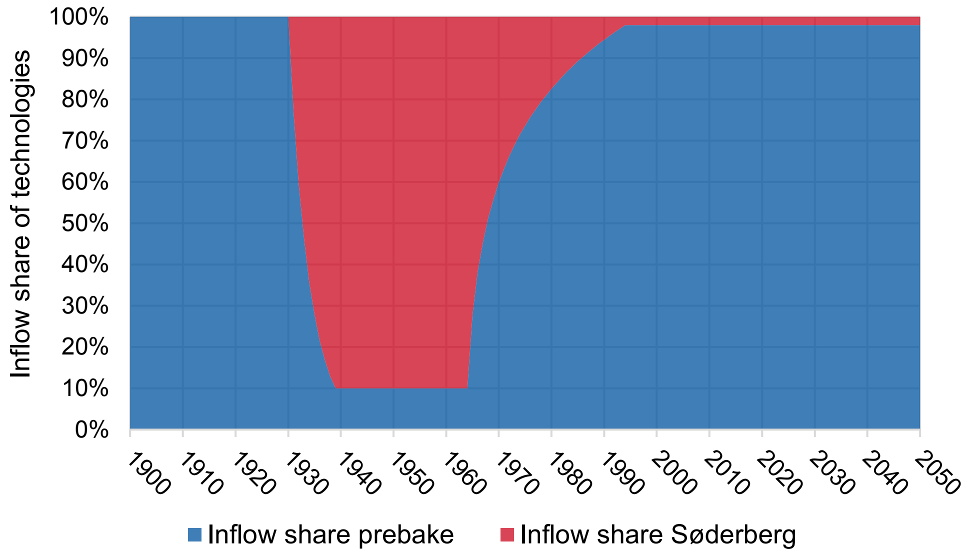

Figure S14: Estimated inflow shares of prebake and Søderberg technology based on [37,38,40–42]

The actual inflows of the prebake and Søderberg technologies are then calculated based on the estimated inflow shares in the cohorts and the total inflows.

$$F_{0-T1,PB,\,c} = \mathrm{F}_{0-\mathrm{T1,c}} * x_{inflow\,PB,c} \qquad 37$$

$F_{0-T1,PB,c}$ Inflow of prebake capacity of cohort c [kt]$\mathrm{F}_{0-\mathrm{T1,c}}$ Inflow of total smelter capacity of cohort [kt/a]
$x_{inflow\,PB,c}$ Share prebake technology in cohort c

$$F_{0-T1,SS,\,c} = \mathrm{F}_{0-\mathrm{T1,c}} * x_{inflow\,SS,c} \qquad 38$$

$F_{0-T1,SS,ct}$ Inflow of Søderberg capacity of cohort [kt]$\mathrm{F}_{0-\mathrm{T1,c}}$ Inflow of total smelter capacity of cohort [kt]
$x_{inflow\,SS,c}$ Share Søderberg technology in cohort c

By using the ODYM model framework [12], a time-cohort matrix for the total smelter stock can be calculated. The time-cohort matrix is a two-dimensional matrix, describing the cohort composition of the stock for each year. A cohort describes the period or year in which an inflow entered the stock. The general concept of a time-cohort matrix is shown in Figure S15. For instance, the $S_{T,0}$ entry describes the remaining amount of cohort 0 (the inflow which entered the stock in period 0) in the stock at time T. The $S_{T,C}$ entry describes the stock in period T from cohort C. The time-cohort matrices for the prebake and Søderberg smelters stock can then be calculated using previously estimated inflow or cohort shares. This is exemplary shown for the prebake smelter in equation 39, whereby the calculation for the Søderberg smelter would follow the same procedure. The calculation based on the time-cohort matrix, as used here, is a simplification which can only be used when the lifetime is equal for all types in the cohorts. Based on this, the stock of the respective technology can be calculated for each year (eq. 40).

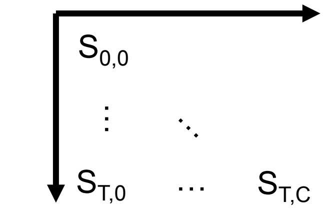

Figure S15: Description of time-cohort matrix over N periods [43]

$$S_{t,c,PB} = S_{c,t} * x_{inflow\,PB,c} \qquad 39$$

$S_{t,c,PB}$ Prebake smelter stock in year t from cohort c$S_{t,c}$Total smelter stock in year t from cohort c

$$S_{T1,PB,t} = \sum_{c=0}^{c=t} S_{c,t,PB} \qquad 40$$

$S_{T1,PB,t}$ Prebake smelter stock in year t$S_{c,t}$Total smelter stock from cohort c in year t

To study the impact of inert anodes on the future smelter stock, different market penetration and retrofitting projections are used. It is assumed that inert anodes would replace or would be built instead of the prebake smelters. The 2 % inflow share of Søderberg smelters is not changed. The inflow of inert anode capacity is thus based on two different types. For new smelter capacity, different market penetration rates or inflow shares of inert anode technology within the inflows of prebake technology are assumed. In addition, the existing prebake capacity can be retrofitted to inert anode technology. For the retrofitting, annual amounts of retrofitted capacity are assumed in different developments.

The stock of new built smelters using inert anodes is calculated based on the stock-cohort matrix of the prebake smelters and the inflow shares of inert anodes in the inflowing prebake smelters (eq. 41). For the retrofitting, it is assumed that existing smelters are retrofitted to inert anode cells and that the retrofitting does not affect the lifetime of the smelter. Due to this simplification, the stock of retrofitted smelters can be calculated by cumulating the annual amount of retrofitting. Thus, the stock-share of inter anode smelters in relation to the total smelter stock can be calculated as shown in eq. 42. Also, the remaining stock share can be calculated (eq. 43).

$$S_{t,c,IA} = S_{t,c,PB} * x_{inflow\ IA\ in\ PB,c} \qquad 41$$

$S_{c,t,IA}$ Inert anode smelter stock in year t from cohort c$x_{inflow\ IA\ in\ PB,c}$ Inflow share inert anode smelter in prebake smelter from cohort c

$$x_{stock,\ IA,t} = \frac{\sum_{c=0}^{c=t} S_{c,t,IA} + \sum_{0}^{t} RF_{IA,t}}{S_{T1,t}} \qquad 42$$

$x_{stock,\ IA,t}$	Stock-share of inert anode technology in year t
$RF_{IA,t}$	Retrofitting to inert anode smelter technology in year t [kt/a]
$S_{T1,t}$	Total stock of smelter capacity in year t [kt]

$$x_{stock,\ rest,t} = 1 - x_{stock,\ IA,t} \qquad 43$$

$x_{stock,\ rest,t}$ Remaining stock-share not using inert anodes in year t

Based on the derived stock composition, the direct GHG emissions of the global smelters can be calculated. As a simplification and because the share of Søderberg technology is small, no distinction is made between prebake and Søderberg technology in terms of the direct emissions. In fact, the PFC emissions are different for Søderberg smelters (0,5 - 0,54 $kg_{CO2\text{-}eq.}$/t Al for 2019) and prebake smelters (0,16 $kg_{CO2\text{-}eq.}$/t Al for non-China PFPBs and 0,8 $kg_{CO2\text{-}eq.}$/t Al for PFPBs in China in 2019) [40]. The global average PFC emissions were 0,55 $kg_{CO2\text{-}eq.}$/t Al. Further it is assumed that in inert anode smelters, direct emissions for both, anode consumption and the PFC-emissions would be eliminate. The total direct GHG emissions can thus be calculated based on the total calculated primary production, the specific direct emissions for non-inert anode smelter and the stock-share of non-inert anode smelter as shown in equation 44. The specific direct GHG emissions consist of emissions related to the anode consumption and PFC emissions. The specific emissions due to anode consumption are assumed to be constant at 1,4 $t_{CO2\text{-}eq.}/t_{Al}$. The average PFC emissions are reported by IAI [40] for 1990 (5,06 $t_{CO2\text{-}eq.}/t_{Al}$) until 2019 (0,55 $t_{CO2\text{-}eq.}/t_{Al}$. It is assumed that the PFC emissions decrease to a value of 0,1 $t_{CO2\text{-}eq.}/t_{Al}$ in 2050. This is based on the lowest value for today's smelter reported in IAI's Anode Effect Survey [44]. Hereby, it was assumed that the PFC emissions would decrease over time and not over the cohorts. However, this assumption can be questioned since it is likely that the PFC emissions would depend on the cohort, so the year of construction. The development of both emissions and the resulting total amount of direct GHG emissions is shown in Figure S16.

$$\begin{aligned} GHG_{smelter,\,direct,t} &= Primary\ Al_t * GHG_{direct,\,specific,t} * x_{stock,\,rest,t} \\ &= (F_{1-4,\,t} + F_{1-5,t}) * GHG_{direct,\,specific,t} * x_{stock,\,rest,t} \end{aligned} \quad 44$$

$GHG_{smelter,\,direct,t}$ Total direct GHG emissions in year t [$Mt_{CO2\text{-}eq.}$]

$GHG_{direct,\,specific,t}$ Specific direct GHG emissions in year t [$t_{CO2\text{-}eq.}/t_{Al}$]

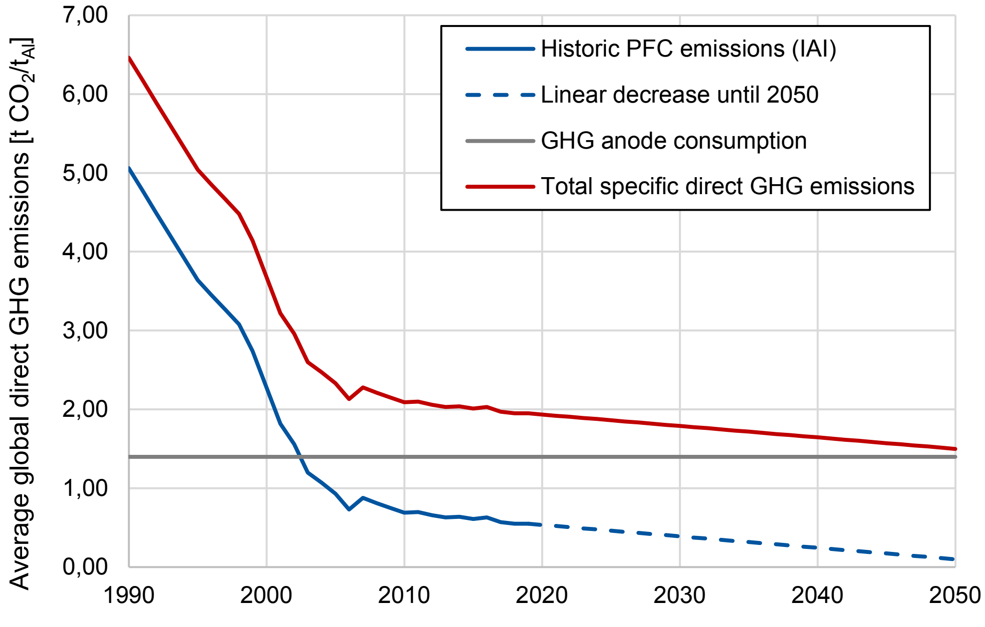

Figure S16: (Estimated) global average for specific direct GHG emissions based on [4,40,44]

The indirect GHG emissions resulting from the electricity demand of the smelter are based on the total energy demand ($E_{smelter}$) and the electricity mix (equation 46). The total energy demand can be derived from the specific energy demand and the total primary production (equation 45). As a simplification, the same average specific energy demand is assumed for all smelting technologies, although smelter using inert anodes might have an increased energy demand. In order to account for different potential developments in the state-of-the-art energy demand, different projections or developments are used for this.

To calculate the global average specific energy demand ($e_{specific}$), estimates of the best available technique (BAT) specific energy demand are used. These are assigned to the cohorts, so the inflowing smelter capacity of the specific year, assuming that new built smelters were using the BAT. The BAT specific energy demand is estimated based on literature and industrial figures. A regression curve of the BAT specific energy is used to estimate the long-term trend, which is shown in Figure S17. The regression also agrees with literature data for the years between 1910 and 1940 [45]. This curve is defines then the specific energy of the respective cohort.

Since the industrial values are describing DC (direct current) values excluding losses for the rectification from AC to DC, they are lower than the average energy demand reported by the IAI in AC (alternating current) values. To achieve a similar average energy demand for 2020, a correction value of about 1,1 kWh/kg_{Al} is added to the regression curve for all years. This corresponds well with today's difference between the DC and AC value of about 0,9 kWh/kg_{Al}. The correction value was derived backwards after quantifying the system. Note, that the adjusted BAT energy demand is still below the IAI average in the figure below because of an inflow delay. This means, that older technology with a higher energy demand remain in the system for several years.

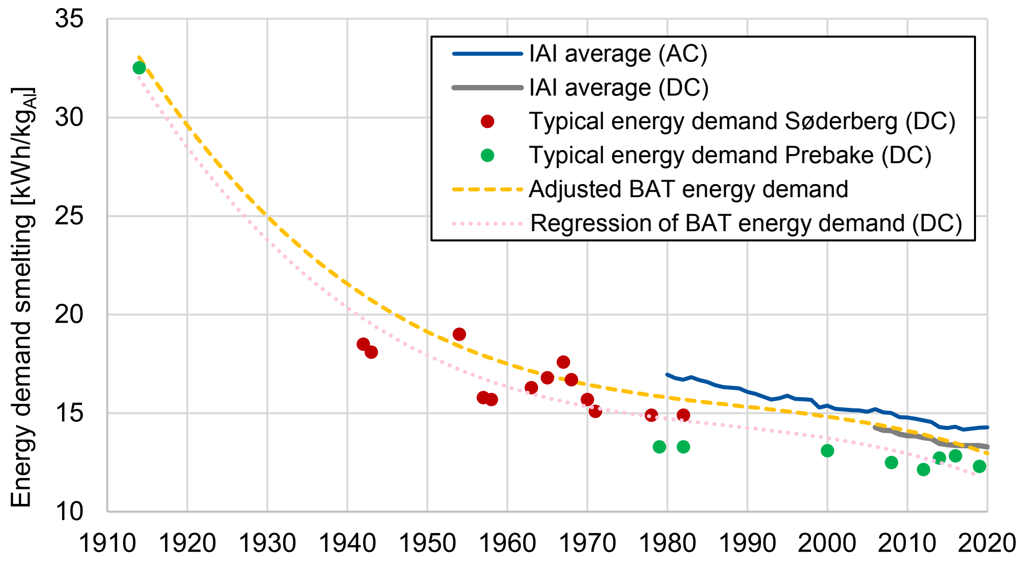

Figure S17: Estimated and adjusted BAT energy demand for the global smelters

The historic carbon intensity of the electricity generation is based on IAI's data [46] for the global shares of the respective energy carriers together with the respective emission factors taken from IPCC [47]. These emission factors include upstream processes for the electricity generation.

For the potential future development of the electricity-mix used for the smelters, different projections are used.

$$E_{smelter,t} = Primary\ Al_t * e_{specific,t} = (F_{1-4,\ t} + F_{1-5,t}) * e_{specific,t} \qquad 45$$

$E_{smelter,t}$ Total electricity demand of global smelters in year t [GWh]$e_{specific,t}$Average specific electricity demand in year t [kWh/kg$_{Al}$]

$$GHG_{smelter,\ indirect,t} = E_{smelter,t} * CI_{EL,t} \qquad 46$$

$GHG_{smelter,\ indirect,t}$ Total indirect GHG emissions (electricity) in year t [Mt$_{CO2\text{-}eq.}$]
$CI_{EL,t}$ Carbon intensity of electricity generation (E-mix) in year t [kt$_{CO2\text{-}eq.}$/GWh]

Assumptions

For an overview of all assumptions made to model and calculate the technology stock system of the global smelters, these are listed in the following:

1. The demand for smelter capacity follows the demand for primary aluminium production.
2. The utilization rate is assumed to increase linearly to 90 % in 2030 and is assumed equal to 1994 level (87 %) for all years before 1994.
3. The same utilization rate for all smelters is assumed.
4. Prebake and Søderberg technologies have the same lifetime.
5. Søderberg smelters are assumed to remain at an inflow share of 2 % for future years.
6. PFC emissions are assumed to decrease linearly to 0,1 t_{Co2}/t_{Al} in 2050 (the today's BAT).
7. Direct emissions due to anode consumption are assumed to be constant at 1,4 t_{CO2}/t_{Al} for all years.
8. No distinction is made for prebake and Søderberg smelters in terms of specific direct emissions.
9. Inert anodes only replace or are used instead of prebake and not Søderberg cells.
10. Inert anodes have no direct emissions.
11. Inert anodes can be used from 2030 onwards.
12. All technologies (prebake, Søderberg, inert anodes) are assumed to have the same energy demand.

Projections of key parameters

To analyse how the technology stock of the global smelters may develop until 2050, different projections of the key parameters are used. To analyse the impact of the smelter lifetime on the stock development and inflows and outflows of smelter capacity, two different lifetime projections are used. To study the impact of inert anodes on the direct emissions, different projections for potential market penetration (addressing new inflows of production capacity), which is the inflow

share of inert anode technology in the prebake smelters, and retrofitting (technology of existing production capacity is changed) are defined. Indirect emissions depend on the specific energy demand and the carbon intensity of electricity generation, so the electricity-mix. Figure S18 gives an overview of the key parameters studied here.

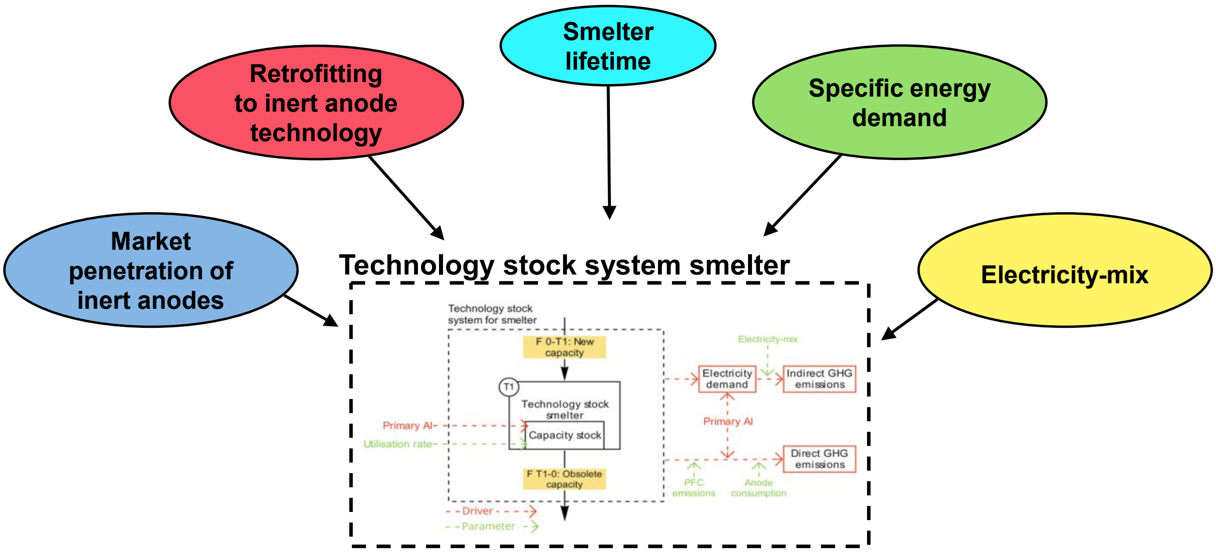

Figure S18: Key parameters for the future development of the global smelter system

Smelter lifetime

The lifetime of the smelter is difficult to estimate and can vary from plant to plant. Therefore, two different lifetimes for the smelters of 40 years in a “low lifetime” and 50 years in a “high lifetime” projection are used in this study. A standard deviation of 13 and 17 years respectively is assumed to account for the uncertainty. This is based on mean age of 40 years of closed Søderberg smelters in North and South America which are reported with start-up and closure date by Barber and Tabereaux (2014) [39]. In the Ecoinvent report (2009) [48], an average lifetime of 50 years is reported for an aluminium electrolysis plant, whereby individual components can have a shorter lifetime. For the standard deviation, 30 % of the mean lifetime was used based on the procedure of Liu et al. (2013) [6] for the aluminium product categories.

Market penetration and retrofitting of inert anodes

Inert anodes (IA) could eliminate the direct emissions from aluminium smelters. However, this technology is still at the development stage and is being tested at an industrial scale [49]. Although the improvements regarding the implementation of inert anodes is uncertain, they are expected to be fully commercialised by 2030 [50]. Elysis [51], a joint venture between Alcoa and Rio Tinto, as well as Rusal [52] are currently researching the development of inert anodes. Together these companies produced around 11,4 Mt primary aluminium in 2020, around 17 % of the global primary production. It could be possible, that the technology would be sold to other companies when these companies have success. China’s research progress in this field is uncertain and not reported. Furthermore, it is uncertain how complex it would be to retrofit the prebake cells with inert anode technology. Elysis [51] for instance, claim that inert anode cells are designed for retrofitting existing smelters with a drop-in replacement. On the other hand, when also combining the inert anode technology with other improvements, such as wettable cathodes etc, it could be more complex to upgrade or retrofit the existing cells. Furthermore, when retrofitting is more

complex and thus the respective cells need to be closed for the period of retrofitting, only a certain amount of smelter capacity could be retrofitted at once, i.e., in one year. For instance, Luo (2008) [53] assumes that no more than 10 % of the total capacity could be retrofitted each year. This would correspond to 7,6 Mt retrofitted capacity when using the USGS capacity [34] for 2021. However, this amount of retrofitting each year is only likely when there are high incentives to make this retrofitting reasonable.

Due to these uncertainties, different projections for the market penetration rate of inert anodes and the yearly amount of retrofitting are used. In general, it is assumed that inert anodes could be implemented from 2030 onwards. The market penetration rate of the inert anodes corresponds to the share of inert anodes in the inflowing prebake capacity.

For the market penetration rate of inert anodes, a "high" and a "medium" projection are defined. In "high" it is assumed that from 2030 onwards only inert anodes are used in new smelter capacity (excluding the Søderberg smelters). In "medium" it is assumed that in 2030 inert anodes are used in 20 % of the new built smelters (excluding the Søderberg smelters) increasing to 80 % in 2040 and remaining at this level until 2050. This should represent a case where not all companies might use the inert anode technology although it is successful and used by others. In addition, a "no inert anodes" development is used to show the development when no inert anodes are used in the future. The projections for the market penetration of inert anodes are shown in Figure S19.

In contrast to the previously mentioned 10 % of yearly retrofitting, the yearly retrofitting is assumed to be at 2 Mt annually from 2030 onwards in a "high" projection in this study. This correspond to about 2,6 % of the USGS [34] reported global smelter capacity for 2021. In a "medium" projection, the yearly retrofitting would be at 1 Mt smelter capacity annually. In "no retrofitting", no additional retrofitting takes place. The projections for retrofitting of the smelter to inert anode technology are shown in Figure S20.

In total, 7 different pathways can be obtained using these parameters, whereby no retrofitting takes place in the "no inert anodes" market penetration.

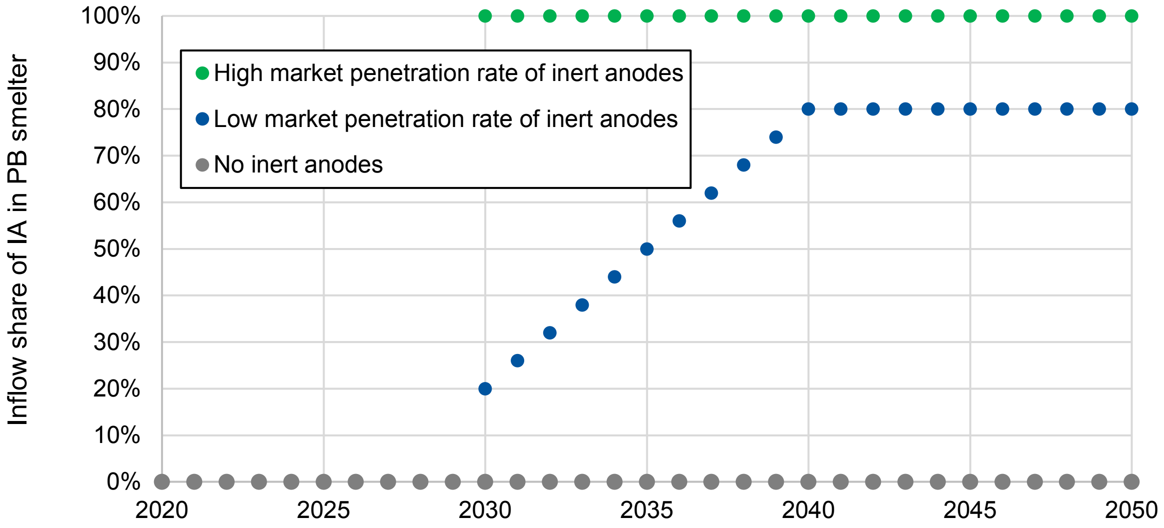

Figure S19: Market penetration rates for inert anode projections

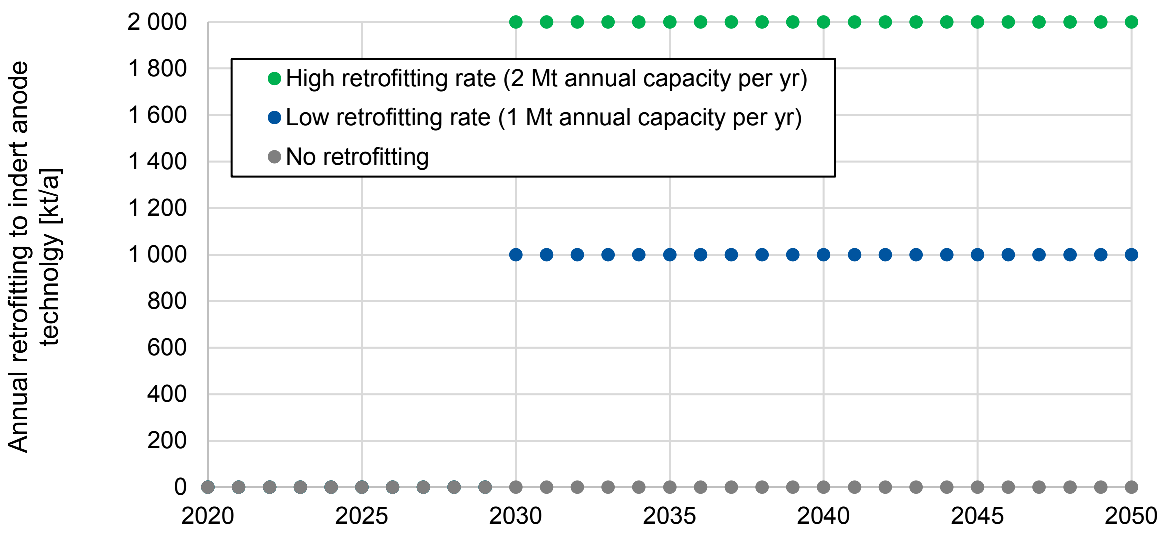

Figure S20: Retrofitting rates for inert anodes projections

Specific energy demand

To study the impact of improvements in state-of-the-art technology for the smelter, different projections for the future development of the BAT technology are used. These continue from the adjusted BAT energy demand from Figure S17, which is at 12,96 kWh/kg_{Al} for the year 2020. In a "moderate efficiency improvements" projection, it is assumed that the BAT energy demand is decreasing linearly to 12,14 kWh/kg_{Al}, which is the lowest energy demand found for today's smelter operating in China [38], in 2030. Afterwards the BAT energy demand remains on this level. In "high efficiency improvement", the same development until 2030 is assumed followed by a further linear decrease to 11 kWh/kg_{Al}. This value is based on reported potential energy demand for inert anode cells in combination with vertical electrode cell (VEC) [54–56]. In a "efficiency BAU" projection it is assumed that the BAT energy demand is not decreasing further, whereby the average energy demand would still decrease when older technology is replaced. The developments of the estimated BAT energy demand in these projections are shown in Figure S21.

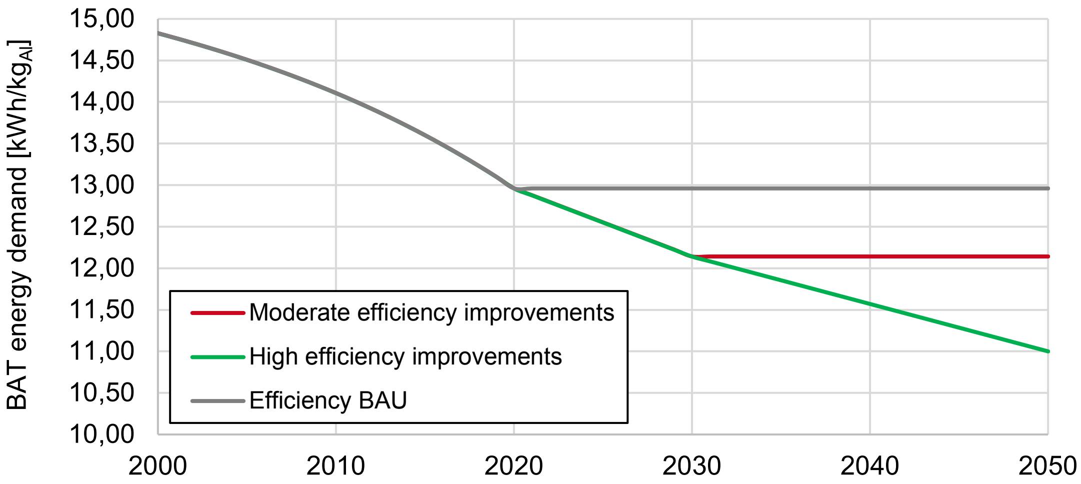

Figure S21: BAT primary smelter energy demand scenarios

Electricity-mix

Due to the high electricity demand of the primary production, the carbon intensity of the electricity generation or the electricity-mix (E-mix) used for the smelter has a crucial role in terms of the total GHG emissions of the primary production. The carbon intensity depends on the energy source of the electricity used in the smelters and the respective carbon emission factors. The historic average global E-mix of the smelter is reported by IAI [46] for the years 1980 until 2020. It is important to know, that the E-mix used by the smelter differs significantly from the average E-mix used for other applications of electricity (other industries, households, etc.). Due to the high electricity demand, smelters are often located close to economical energy sources such as hydro power [57]. This is leading to another electricity-mix for smelters (shown in Figure S22) compared to the global average electricity-mix. However, most of the electricity for aluminium smelter today is generated by coal (55,7 %), followed by hydro power (30 %). The share of coal based electricity increased significantly in the last decades due to the increased primary production in China where 82 % of the electricity for the smelter is based on coal plants [46].

To study the future development of the E-mix, two different projections are used. The “B2DS E-Mix” projection follows the “Beyond 2 Degree Scenario” (B2DS) published by the International Energy Agency and used in IAI’s GHG Pathway to 2050 [58]. In the “B2DS” scenario, electricity generation with coal would be reduced to 4 % in 2050 while nuclear and especially renewable energy sources are increasing. IAI’s B2DS E-mix for 2050 is shown together with the historical data in Figure S22. For the transition between 2020 and 2050 a linear interpolation is used in this model. The second projection is the “BAU E-mix”. The “BAU E-mix” follows the IAI’s “BAU” scenario [58] where the E-mix remains at the 2020 level until 2050.

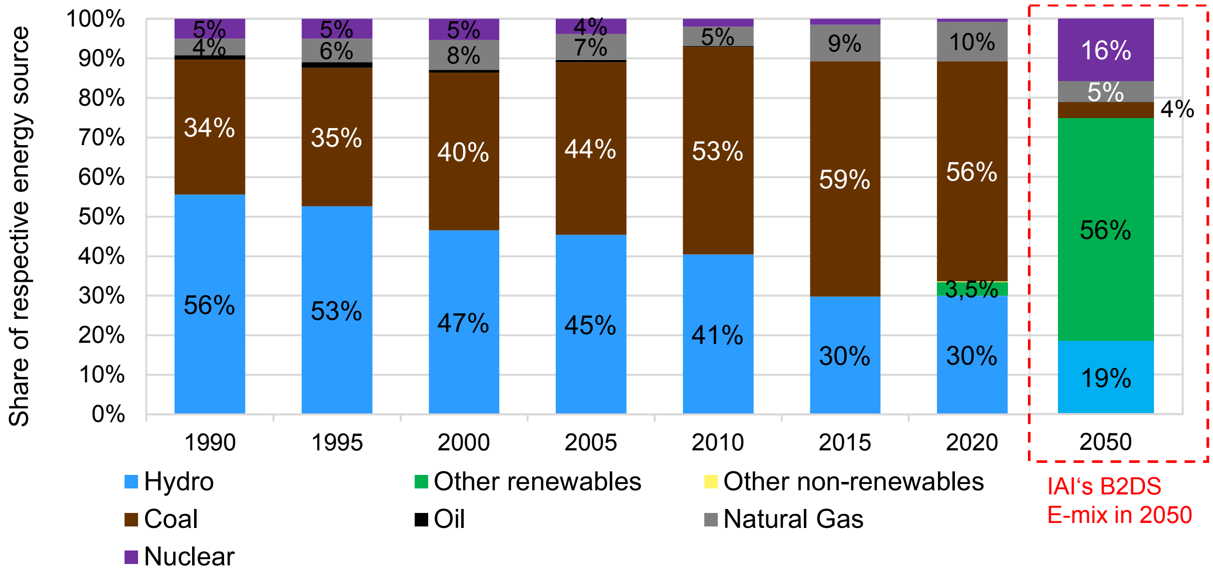

Figure S22: Historic global average electricity-mix for smelter and IAI’s B2DS scenario for 2050 [46,58]

To derive the resulting GHG emissions of the respective electricity-mix, GHG emission factors for the electricity generation compiled and reported by the IPCC [47] are used. Separate emission factors for the different energy sources are used here in order to take a changing electricity-mix

into account. The IPCC emission factors are based on a review of numerous published lifecycle assessments (LCAs) and reported with a minimum and maximum value as well as 25th, 50th and 75th percentiles. For this study, the 50th percentile figures of the GHG emission factors are used, which means that 50 % of all included data is below (or equal to) this value. The IPCC data is shown in Table S10. For the carbon intensity of the smelter electricity-mix in this study, mean values of this data are used when the respective energy source is not separately reported. For solar energy, a mean value of the PV (photovoltaic) and CSP (concentrating solar power) is used. For “other renewables” a mean value of the wind and solar energy is assumed in this study. “Other non-renewables” are treated as natural gas. The resulting GHG emission factors used in this study are shown in Table S11. Due to the included upstream emissions, the GHG emission factor for electricity generated by natural gas is higher than the emission factor used for natural gas used as a fuel for the melting furnaces (202 $g_{CO2\text{-}eq.}$/kWh [59,60]) in this study. There, only direct emissions (so scope 1 [61]) are included.

Table S10: IPCC GHG emission factors of different energy sources for electricity generation (50th percentiles) in $g_{CO2\text{-}eq.}$/kWh [47]

	Bio-power	Solar		Geothermal.	Hydro	Ocean	Wind	Nuclear	Natural gas	Oil	Coal
		PV	CSP								
GHG factor [$g_{CO2\text{-}eq.}$/kWh]	18	46	22	45	4	8	12	16	469	840	1001

Table S11: GHG emission factors for electricity generation by energy source used for smelter electricity-mix

	Hydro	Coal	Nuclear	Natural gas	Oil	Other renewables	Other non-renewables
GHG factor [$g_{CO2\text{-}eq.}$/kWh]	4	1001	16	469	840	23	469

Combining the GHG emission factors of Table S11 with the previously defined electricity demand projections (“B2DS E-mix” and “BAU E-mix”), the GHG emissions for the electricity used in the smelters can be derived. The developments of the respective projections are shown in Figure S23.

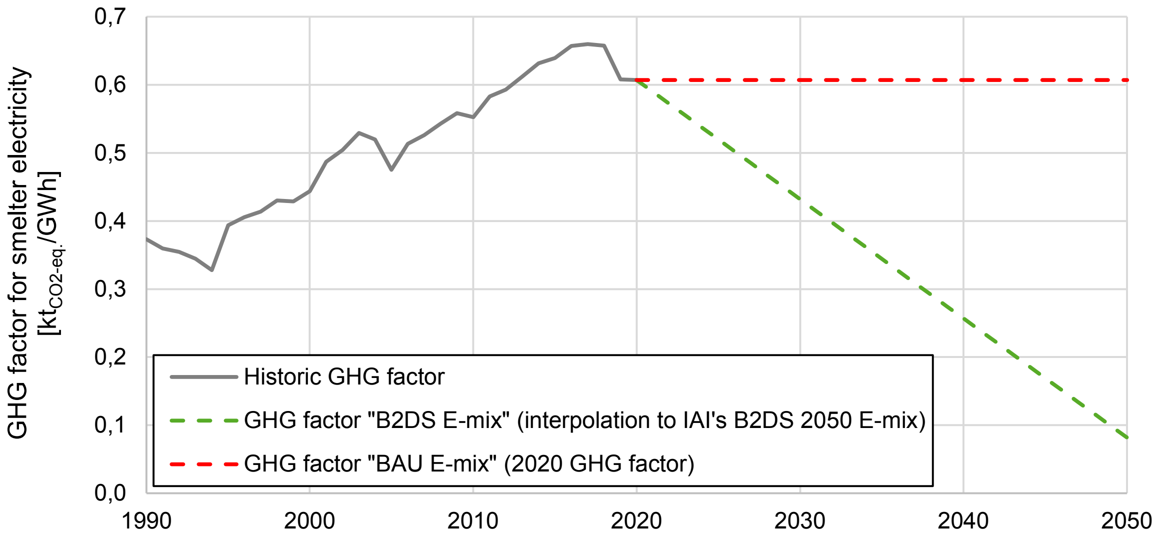

Figure S23: GHG emission factors for electricity usage in smelter (E-mix projections)

Overview of the key parameters in the smelter technology system

An overview of the parameters of the smelter technology system, which are presented in this chapter and are changed in future scenarios, is shown in Table S12.

Table S12: Overview of the key parameters in the smelter technology system

Parameter	Parameter choice	Description
Smelter lifetime	Low lifetime	40 years mean lifetime with 13 years standard deviation (30 % of mean value)
	High lifetime	50 years mean lifetime with 17 years standard deviation (30 % of mean value)
Market penetration rate (MPR) of inert anodes	No inert anodes	No inert anodes are used
	Low MPR	20 % inflow share in 2030 increasing linearly to 80 % in 2040. Constant on 40 % for 2040 - 2050
	High MPR	100 % inflow share in new prebake smelters from 2030 onwards
Retrofitting (RF) to inert anode technology	No RF	No production capacity is retrofitted to inert anode technology
	Low RF	1 Mt production capacity is retrofitted to inert anode technology
	High RF	2 Mt production capacity is retrofitted to inert anode technology
Specific energy demand	Efficiency BAU	BAT technology is not improving further
	Moderate efficiency improvements	BAT technology improves to a specific energy demand of 12,14 kWh/kg_{Al} for primary production
	High efficiency improvements	BAT technology improves to a specific energy demand of 11 kWh/kg_{Al}
Smelter electricity-mix (E-mix)	BAU	E-mix of smelters remains at 2020 level
	B2DS	E-mix of smelters in 2050 evolves to the B2DS (Beyond 2 Degrees) E-mix from IAI [58], where mostly renewable energy sources are used. Years in between are linearly interpolated.

A.3 – Technology layer secondary melting

System definition and description of flows, stocks and parameters

The secondary melting is divided into three types of melting processes, namely remelter (T2), refiner (T3) and foundry (T4), as shown in Figure S24. This is based on the defined processes in the global aluminium cycle, whereby the foundry corresponds to the internal refining in the shape-casting. As for the smelter stock before, the melting processes aggregate the global production capacity of all respective types of melting furnaces. The necessary capacity stock of these processes is what drives the inflows of new capacity and is defined by the throughput of the respective processes in the aluminium cycle. In contrast to the smelters, no utilization rate is included in the melting system due to missing data on this.

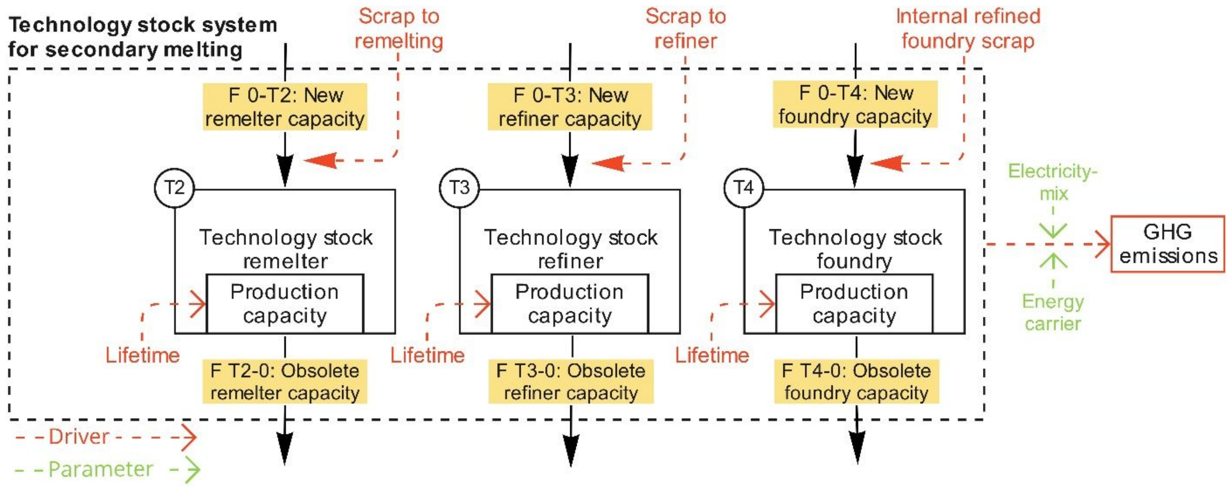

Figure S24: Simplified system definition for secondary melting technology stock

The inflowing capacity for the three melting processes ($F_{0\text{-}T2}$, $F_{0\text{-}T3}$, $F_{0\text{-}T4}$) describes the capacity which needs to be added per year due to a stock increase or due to the replacement of obsolete capacity. As for the smelter, the type of inflowing technology can vary. While today mostly natural gas furnaces are used, an increased usage of electric heated or hydrogen fired furnaces could be possible in a future development.

Different furnace types for today's industrial practice and for potential future developments or projections are defined for the respective melting process. This is based on today's state of the art technology and assumptions on the today's stock or inflow composition and how this may evolve in future projections. For the remelter, natural gas fired reverberatory and inductively heated furnaces are used today with hydrogen fired furnaces in potential future projections. In refiners, rotary drum and reverberatory furnaces are used today. Inductive and hydrogen fired furnaces are used for potential future projections in the refiner. In foundries, shaft furnaces, fossil fired crucibles, inductively heated crucibles and reverberatory furnaces are used today and hydrogen fired furnaces in potential future projections.

The outflows of obsolete capacity ($F_{T2\text{-}0}$, $F_{T3\text{-}0}$, $F_{T4\text{-}0}$) represent retiring capacity whose lifetime is over. Thereby, the same lifetime for all melting furnaces or technologies is assumed. The description of a furnace lifetime is similar to that of the smelter lifetime.

The throughput of secondary melted aluminium in these three processes together with the specific energy demand of the different furnace types defines the total energy demand. The resulting GHG emissions are then based on the energy demand of these processes and the energy carriers used in the furnaces or for electricity generation.

List of system variables

The system variables used in the secondary melting system are listed in Table S13 together with sub-variables. In total, there are three main inflows and three main outflows, three stocks representing the different melting capacities and the respective stock changes. The sub-variables represent the specific types of melting furnaces in the flows or stocks.

Table S13: List of system variables and sub-variables of the secondary melting system

Label of system variable/ sub-variable	Explanation	Type
$\mathbf{F_{0\text{-}T2}}$	**Total inflow of new remelter capacity**	**Flow**
$F_{0\text{-}T2,\ reverberatory}$	Inflow of remelter capacity using reverberatory furnaces	Flow
$F_{0\text{-}T2,\ induction}$	Inflow of remelter capacity using induction furnaces	Flow
$F_{0\text{-}T2,\ H2}$	Inflow of remelter capacity using hydrogen furnaces	Flow
$\mathbf{F_{0\text{-}T3}}$	**Total inflow of new refiner capacity**	**Flow**
$F_{0\text{-}T3,\ rotary}$	Inflow of refiner capacity using rotary furnaces	Flow
$F_{0\text{-}T3,\ reverberatory}$	Inflow of refiner capacity using reverberatory furnaces	Flow
$F_{0\text{-}T3,\ induction}$	Inflow of refiner capacity using induction furnaces	Flow
$F_{0\text{-}T3,\ H2}$	Inflow of refiner capacity using hydrogen furnaces	Flow
$\mathbf{F_{0\text{-}T4}}$	**Total inflow of new refiner capacity**	**Flow**
$F_{0\text{-}T4,\ shaft}$	Inflow of foundry capacity using rotary furnaces	Flow
$F_{0\text{-}T4,\ crucible\ fossil}$	Inflow of foundry capacity using rotary furnaces	Flow
$F_{0\text{-}T4,\ induction}$	Inflow of foundry capacity using rotary furnaces	Flow
$F_{0\text{-}T4,\ reverberatory}$	Inflow of foundry capacity using rotary furnaces	Flow
$F_{0\text{-}T4,\ H2}$	Inflow of foundry capacity using rotary furnaces	Flow
$F_{T2\text{-}0}$	Total outflow of obsolete remelter capacity *	Flow
$F_{T3\text{-}0}$	Total outflow of obsolete refiner capacity *	Flow
$F_{T4\text{-}0}$	Total outflow of obsolete foundry capacity *	Flow
S_{T2}	Total stock of remelter capacity *	Stock
S_{T3}	Total stock of refiner capacity *	Stock
S_{T4}	Total stock of foundry capacity *	Stock
ΔS_{T2}	Total stock change of remelter capacity *	Stock change
ΔS_{T3}	Total stock change of refiner capacity *	Stock change
ΔS_{T4}	Total stock change of foundry capacity *	Stock change

* with same sub-variables as the respective inflows

Calculations and parameters

The first step to determine the secondary system is to calculate the total melting capacity or the total stock of capacity for the three melting processes. To account for the total necessary capacity including losses, the sum of all scrap flows entering the process are used. This is exemplary shown for the remelter in equation 47. For the foundry melting capacity, the internal refined scrap flow ($F_{4\text{-}4}$) of the global aluminium cycle is used. As mentioned before, no utilization rate is included here.

$$S_{T2,\,t} = Scrap\ to\ remelter_t = F_{6-2,\,t} + F_{8-2,t} + F_{10-2a,t} + F_{10-2b,t} \tag{47}$$

$S_{T2,\,t}$ Total stock of remelter capacity in year t$F_{i-10,\,t}$ Scrap flow from process i to remelter in year t (from Al cycle layer) [kt/a]

To calculate the composition of the different melting stocks, inflow shares for the different furnace types are used for the historic and current inflows. These are shown in Table S14.

Today, there are mainly reverberatory furnaces used in remelter plants [1]. Induction furnaces are used to melt material of smaller sizes such as turnings [62]. Boin and Bertram (2005) [7] list the scrap types from fabrication which are allocated to remelter in the EU. Based on this it is derived that around 10 % of the wrought alloy scrap are turnings in their model. Thus, it is assumed that the historic inflows in the remelter process consist of 90 % reverberatory furnaces and 10 % induction furnaces. Of the reverberatory furnaces it is assumed that 80 % single chamber and 20 % twin chamber furnaces are used. This is based on the assumption that mostly clean processing scrap is melted in the remelter and thus can be melted in the single chamber furnaces. For the induction furnaces, coreless as well as channel induction furnaces are assumed to be usable in equal proportions.

The composition of the refiner furnaces is based on the European scrap refining model from European Aluminium [16], where 70 % of the refined scrap is melted in rotary drum furnaces and 30 % is melted in reverberatory furnaces. Thus, this share is also used in this study. Both, fixed axis and tilting rotary drum furnaces are assumed. For the reverberatory furnaces, only twin chamber furnaces are assumed here.

Since no data was available regarding the composition of the melting furnaces in the foundries, it is assumed that shaft, crucible and reverberatory furnaces are used equally. Thereby, crucible furnaces are assumed to be heated inductively and fossil with natural gas in equal shares. It is assumed that only coreless induction furnaces are used, which is based on Schmitz (2006) [63]. For the reverberatory furnaces, only single chamber furnaces are assumed.

The total inflows ($F_{0\text{-}T2}$, $F_{0\text{-}T3}$, $F_{0\text{-}T4}$) and outflows ($F_{T2\text{-}0}$, $F_{T3\text{-}0}$, $F_{T4\text{-}0}$,) of melting capacities are derived in the same way as for the aluminium cycle system and the smelter using a lifetime distribution and a python script based on the ODYM model framework [12]. Thereby the in- and outflows are based on the stock development and annual stock change and an assumed mean lifetime. For the mean lifetime, 20 and 30 years with a standard deviation of 7 and 10 years respectively are assumed in two different projections.

Table S14: Estimated historic and current inflow shares for types of melting furnaces based on [1,7,16,62,63]

Melting process	Furnace type	Assumed historic inflow share in melting process	Comment
Remelter	Reverberatory furnace	90 %	Of this 80 % single, 20 % twin chamber
	Induction furnace	10 %	Coreless and channel
Refiner	Rotary drum furnace	70 %	Fixed axis and tilting
	Reverberatory furnace	30 %	Twin chamber
Foundry	Shaft furnace	33 %	
	Crucible furnace (fossil)	17 %	
	Induction	17 %	Coreless
	Reverberatory furnace	33 %	Single chamber

The inflows of the respective furnace type are calculated based on the historic and potential future inflow shares as shown exemplarily in equation 48 for the reverberatory furnaces used in the remelter.

$$F_{0-T2,reverberatory,\,c} = \mathrm{F}_{0-\mathrm{T2,c}} * x_{inflow\;remelter,\,reverberatory,c} \tag{48}$$

$F_{0-T2,reverberatory,c}$	Inflow of reverberatory furnaces for remelter of cohort c [kt]
$\mathrm{F}_{0-\mathrm{T2,c}}$	Total inflow of new remelter capacity of cohort c [kt]
$x_{inflow\;remelter,\,reverberatory,c}$	Inflow share reverberatory furnaces for remelter in cohort c

The stocks of the respective furnace types (S_{T2}, S_{T3}, S_{T4}) can be derived from the stock-cohort matrix as explained for the smelters.

The respective outflows can be derived from the annual stock change and the inflows. This is shown exemplarily in equation 49 for the reverberatory furnaces leaving the remelter stock.

$$\begin{aligned} F_{T2-0,reverberatory,\,t} &= F_{0-T2,reverberatory,\,t} - \Delta \mathrm{S}_{\mathrm{T2,\,reverberatory,t}} \\ &= F_{0-T2,reverberatory,\,t} - (S_{T2,reverberatory,t} - S_{T2,reverberatory,t-1}) \end{aligned} \tag{49}$$

$F_{T2-0,reverberatory,\,t}$	Outflow of reverberatory furnaces from remelter in year t [kt]Δ
$\mathrm{S}_{\mathrm{T2,\,reverberatory,t}}$	Stock change of reverberatory furnaces in remelter in t [kt]
$S_{T2,reverberatory,t}$	Stock of reverberatory furnaces in remelter in t [kt]

The total energy demand of the melting processes and the global secondary melting system in total are calculated based on average specific energy demands for the respective furnace types. These are based on the state-of-the-art energy demands (Table S15) and the estimations and comments mentioned above. When several values for an energy demand are available or when the furnace types are further differentiated, mean values were used. Since no data is available for the energy demand of hydrogen fired furnaces, the same energy demand as for the natural gas alternatives is used. The resulting energy demands are shown in Table S16 together with the best available technique value (the lowest value found for the respective energy demand).

Table S15: Key parameters for melting furnaces [4,62–68]

Type	Variation	Appli-cation*	Max. capacity [t]	Max. melting rates [t/h]	Energy source	Energy demand melting [kWh/t_{Al}]	References
Reverberatory	Standard (single chamber)	Remelter, Refiner, Primary (M, H)	200	30	Natural gas	575 - 740	4,63,67
	Closed well (twin chamber)	Remelter (M, H)	200	28	Natural gas	675 - 850	4,63,67
Crucible	Fuel-heated	Foundry (M, H)	<1	<1	Natural gas	780 - 1230	4,63,66
	Resistance-heated	Foundry (M, H)	1,2	<1	Elec-tricity	750	4,63,66
	Coreless induction	Foundry, Remelter (M, H)	9	8,8	Elec-tricity	511	63,64
	Channel induction	Remelter, Primary (M)	55	11	Elec-tricity	427 - 1000**	4,62,63,69
Rotary drum	Fixed axis	Refiner (M)	150	20	Natural gas	520 - 1306	4,63
	Tilting	Refiner (M)	30	7	Natural gas	556 - 750	4,63,66
Shaft	-	Foundry	20	5	Natural gas	580 - 917	4,63,68

*M: Melting; H: Holding

**upper value for melting and holding together

Table S16: Mean energy demands for melting furnaces and BAT energy demands

Melting process	Furnace type	Mean energy demand [kWh/t$_{Al}$]	Comment	BAT energy demand [kWh/t$_{Al}$]
Remelter	Reverberatory furnace	679	80 % mean value of recuperative and regenerative for single chamer; 20% mean value of recuperative and regenerative for twin chamber	460
	Induction furnace	466	Mean value channel and coreless	427
	Hydrogen furnace	679	Same as reverberatory assumed	-
Refiner	Rotary drum furnace	783	Mean value fixed axis and tilting	520
	Reverberatory furnace	763	Mean value of recuperative and regenerative twin chamber furnace	525
	Induction	466	50% coreless, 50 % channel assumed	427
	Hydrogen furnace	773	Mean value of rotary and reverberatory assumed	-
Foundry	Shaft furnace	749	748,5	580
	Crucible furnace (fossil)	1005		780
	Induction	511	Coreless	427
	Reverberatory furnace	658	Single chamber	460
	Hydrogen	703	Mean value of recuperative and regenerative single chamber furnace assumed	-

Using these mean values for the specific energy demand of the melting furnaces together with the flow of molten aluminium and the stock shares of the furnace used, the total energy demand of the respective melting furnace can be calculated. The flow of molten aluminium can be taken from the global aluminium cycle system. This is exemplarily shown in equation 50.

$$E_{remelter,reverberatory,t} = \mathrm{F}_{2-4,t} * x_{stock,reverberatory,remelter} * e_{specific,reverberatory,t} = \mathrm{F}_{2-4,t} * \frac{S_{T2,reverberatory,t}}{S_{T2,t}} * e_{specific,reverberatory,t} \quad (50)$$

$\mathrm{F}_{2-4,t}$	Secondary Al from remelter (from global aluminium cycle) [kt]
$E_{remelter,reverberatory,t}$	Energy demand of all reverberatory furnaces in remelter process in year t [GWh]
$x_{stock,reverberatory,remelter,t}$	Share of reverberatory furnaces in remelter stock
$e_{specific,reverberatory,t}$	Specific energy demand of reverberatory furnace [kWh/t$_{Al}$]

Based on the energy demand of the furnaces and the GHG emission factors of the respective energy carrier the resulting carbon intensity and GHG emissions can be derived. For natural gas, a constant emission factor of 202 $g_{CO2\text{-}eq.}$/kWh is used [59,60].

For the electric heated furnaces (induction), the average carbon intensity of the global electricity generation is used. The historic carbon intensity of the electricity generation from 2000 until 2020 is taken from [70], where also the upstream processes are included as for the smelters. In contrast to the smelter, there are no separate electricity-mixes reported for the melting furnaces, so the global average electricity-mix is used. The development is shown in Figure S25. The carbon intensity of the global electricity-mix was peaking in 2007 at almost 490 $g_{CO2/kWh}$ and has been decreasing in the last years until 2020 to around 435 $g_{CO2/kWh}$. For the electricity-mix of the years before 2000, the 2000er figure is assumed (dashed line). For the potential future development, different projections are used.

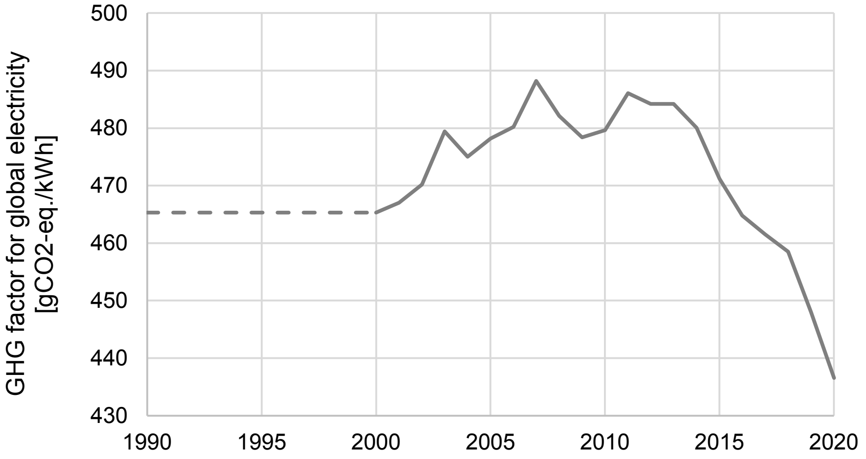

Figure S25: Historic GHG factors for global electricity [70]

For hydrogen heated furnaces the GHG emissions of the hydrogen production needs to be included. Today, most of the hydrogen produced is grey hydrogen which is produced out of natural gas [71]. For grey hydrogen, an GHG emission factor of 353 $g_{CO2\text{-}eq.}$/kWh is used [72]. For (green) hydrogen produced in electrolysers using electricity, electrical efficiencies are used to derive the electricity demand. For the SOEC electrolyser (solid oxide electrolysis cells), which is the latest developed technology, efficiencies as shown in Table S17 are reported [71]. For the years in between, linear interpolation is used. Based on this efficiency and the carbon intensity of the electricity generation (GHG factors), the carbon intensity per kWh hydrogen produced by in electrolysers can be calculated. The GHG factors of the electricity are again based on the potential future projections and the historical data above and the global electricity-mix is assumed. It is assumed that grey hydrogen is used for the years where the resulting carbon intensity is below the carbon intensity of hydrogen produced in electrolysers. This results from the higher GHG factors of the electricity generation compared to grey hydrogen. Hydrogen from electrolysers is assumed to be used when the electricity-mix contains more renewable energy so it’s GHG factor is below the grey hydrogen. Linear interpolation for the years in between results in an energy demand for hydrogen production as shown in Figure S26.

Table S17: Mean electrical efficiencies of SOEC electrolyser

	Today	2030	Long term
Electrical efficiency [%]	77,5	80,5	90

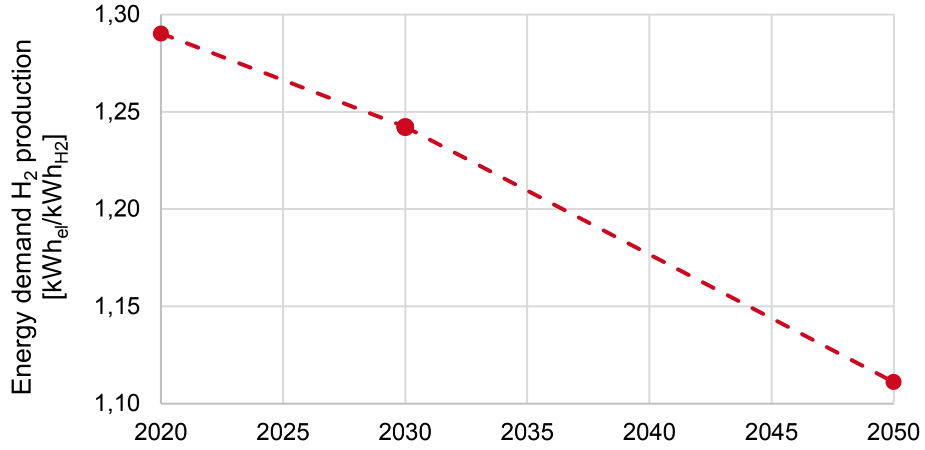

Figure S26: Projected energy demand for hydrogen production based on [71]

Assumptions

For an overview of all assumptions made in order to model and calculate the technology stock system of the global secondary melting, these are listed in the following:

1. The demand for melting capacity follows the amount of secondary production, i.e., the scrap flows which need to be recycled.
2. No utilization rate is assumed for melting furnaces, so no excess capacity of plants not working on full capacity is included.
3. All melting furnaces are assumed to have the same lifetime.
4. The global electricity-mix is used for resulting GHG emissions of electricity consumption.
5. The inflow-/stock shares as presented above are used for the global average. This is based on assumptions and reports regarding EU recycling systems (see above).
6. Hydrogen could be used from 2030 onwards for melting furnaces.
7. Grey hydrogen would be used as long as the GHG emissions are below the (green) hydrogen from electrolysers.

Projections of key parameters

T analyse how the technology stock of the global secondary melting may develop until 2050, different projections are used. To analyse the impact of the furnace lifetime on the stock development and inflows and outflows of melting furnace capacity, two different lifetime projections are used. To study the impact of alternative heating technologies, such as hydrogen and electrically heated furnaces, different market penetration projections are defined. To analyse

the impact of improvements of the specific energy demand, another projection is used. As the electricity-mix defines the carbon intensity of electric and hydrogen heated furnaces, two different projections for its potential development are used. Figure S27 gives an overview of the parameters studied here.

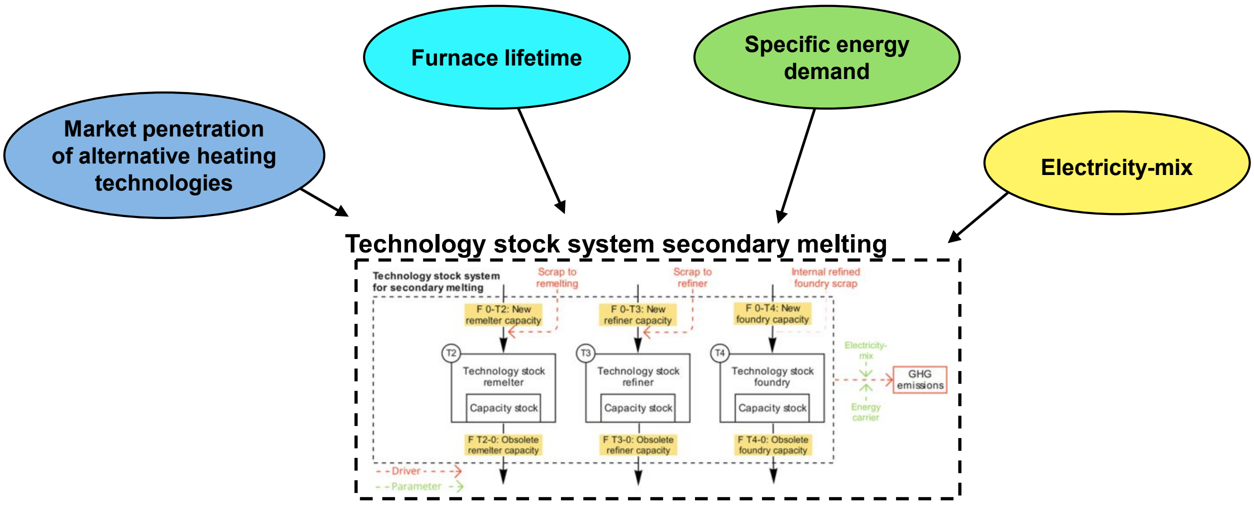

Figure S27: Parameters for future development of the global secondary melting system

Melting furnace lifetime

As for the smelter system, different lifetimes of the melting furnaces are assumed. Again, the data situation on this is poor and the lifetime can vary from furnace to furnace. Thus, two different lifetime estimations or projections are used to study the impact of this on the occurring outflows and resulting inflows and stock compositions. In “low lifetime”, 20 years with a standard deviation of 7 years is assumed. In “high lifetime”, 30 years with a standard deviation of 10 years is assumed.

StrikoWestofen [73] mention 20 years of service life for their shaft melting furnace. In the Ecoinvent report (2009) [48], 25 years of lifetime are reported for an aluminium melting furnace. Bacchetti (2018) [74] report 10 - 20 years for gas fired crucible furnaces, 15 - 25 years for reverberatory furnaces and 20 – 25 years for tower/shaft furnaces in the aluminium sector. For the standard deviation, 30 % of the mean lifetime were used as it is done for the smelters and aluminium product categories.

Market penetration of alternative heating technologies

To study how a change from mostly natural gas heated melting furnaces towards alternative heating technologies may be achieved and how this would impact the energy demand and the resulting GHG emissions, different projections are defined. For this, different time varying inflow shares or market penetration rates for different furnace types and technologies are used as previously explained.

In a “BAU” (business as usual) projection, the furnaces used remain at today’s practice and at the same inflow shares as presented in Table S14, namely mostly natural gas fired furnaces.

In a “Electrification” projection, it is assumed that only electric induction furnaces are used when new melting capacity is built. Today, only a few melting furnaces are heated with induction.

Especially in the refiner, no inductive or other electric heating is used. Induction furnaces are mainly used to melt cleaner scrap or primary feedstock [4,62,63]. The induction furnaces and the energy demand listed in Table S15 are used. Also, the need for channel induction furnaces to have a remaining melt in the channel and the relatively small capacity of coreless induction furnaces could cause difficulties when only using induction furnaces for melting. However, this projection is used to show the most extreme case in terms of electrification of the melting furnaces.

To take the potential usage of hydrogen fired melting furnaces into account, the "Electrification and hydrogen" projection is used. It is assumed that hydrogen heated furnaces are used from 2030 onwards together with inductively heated furnaces. This is based on the expected cost competitiveness of green hydrogen between 2028 and 2035 [75] and that electrically heated furnaces might be the more economically beneficial solution in some cases. In the years from today (2023 included) until 2029 it is assumed that the "Electrification and hydrogen" projection follows the "Electrification" projection and only inductively heated furnaces are used in inflowing and newly built furnaces.

The resulting inflow shares or market penetration rates are visualised in the following:

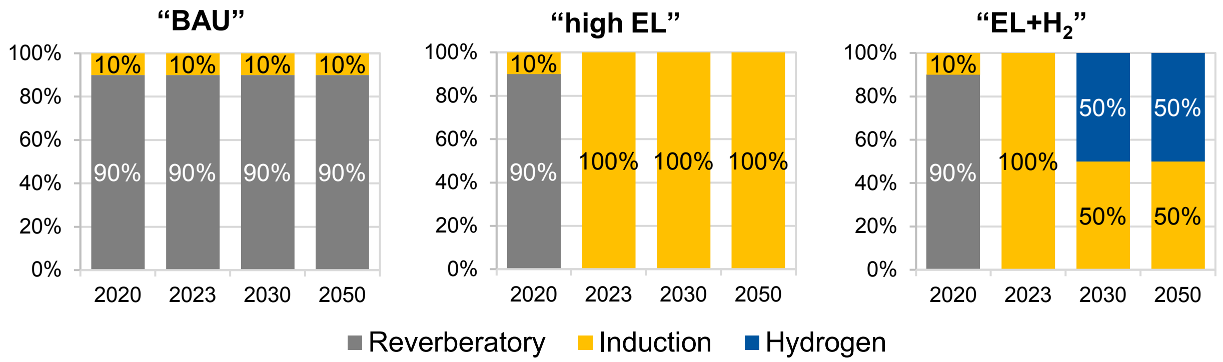

Figure S28: Market penetration rates for remelters

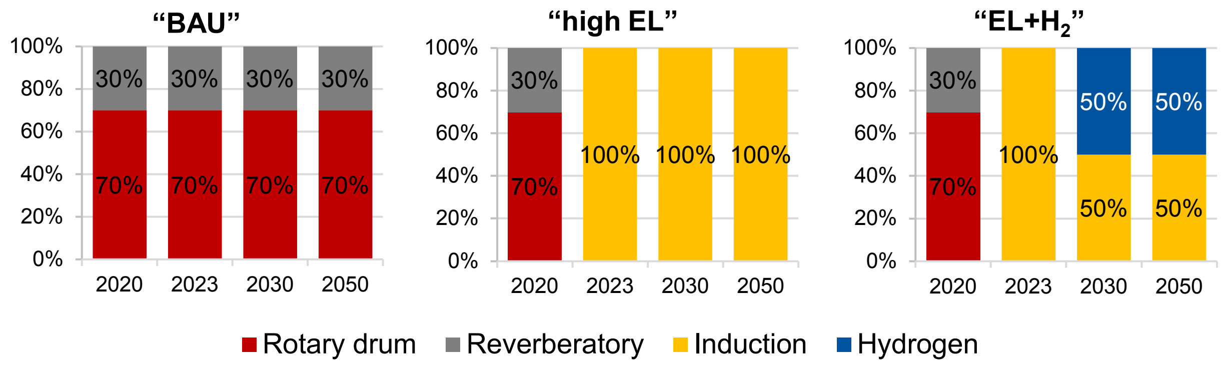

Figure S29: Market penetration rates for refiners

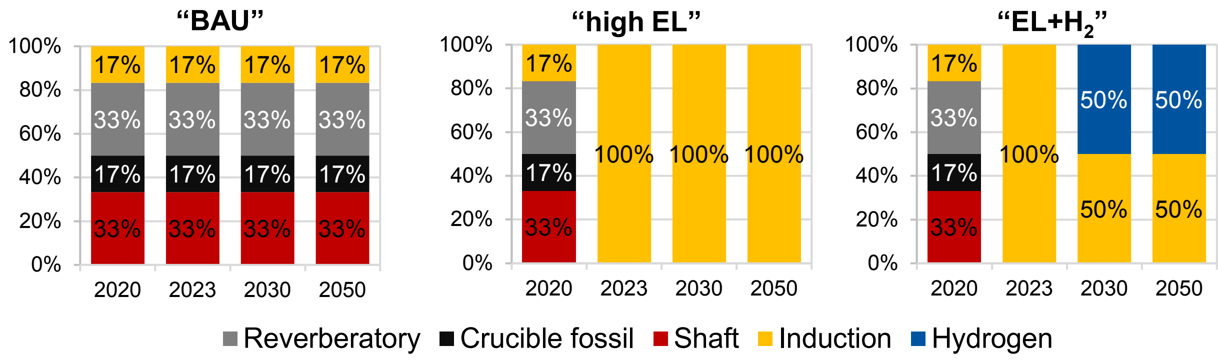

Figure S30: Market penetration rates for foundries

Specific energy demand

To analyse the impact of improved efficiencies of the melting furnaces, an "improved efficiencies" projection is defined. This follows the "BAU" projection from before in terms of furnace types, so mostly natural gas fired furnaces are used. In addition, the mean energy demand of the respective furnace type is decreasing linearly until 2050 to the BAT value presented in Table S16, which means to the lowest energy demand found for today for the respective furnace type (Figure S31). These improvements could be achieved by using more recuperative and regenerative burners for heat recovery, oxy-fuel burner or better insulation.

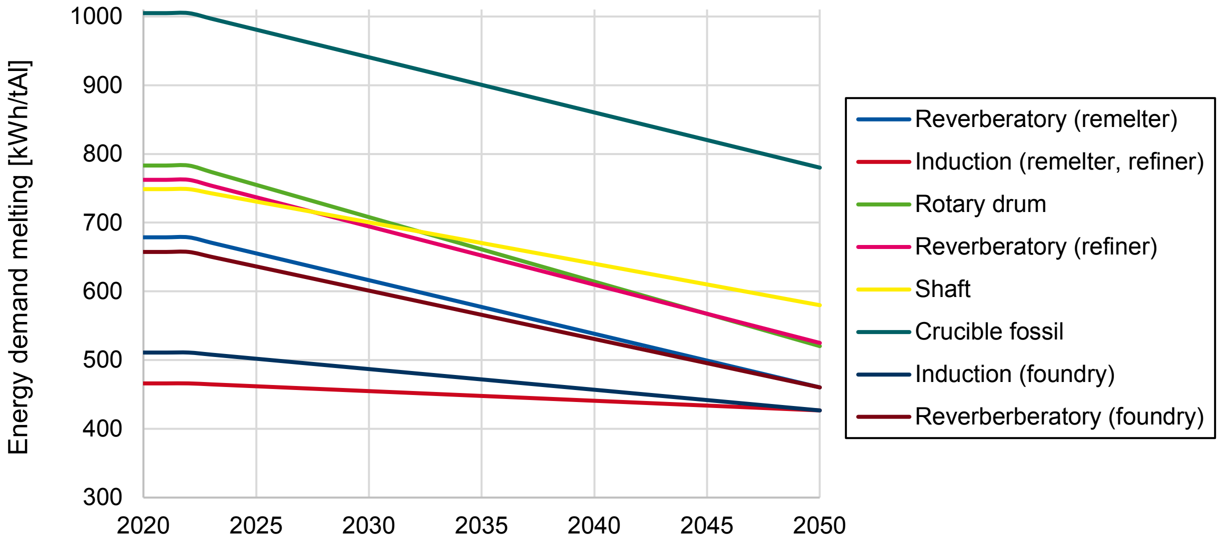

Figure S31: Development of specific melting energy demand to today's BAT energy demand in 2050 (based on values in Table S16)

Electricity-mix

As described before, the global electricity-mix is assumed for the electrically heated melting furnaces and for the hydrogen production contrary to the smelters where a separate electricity mix was used. This is because the electricity-mix for the smelter is reported separately, which is not the case for other industries such as the secondary aluminium industry.

For the global electricity-mix and the resulting GHG emissions, two projections are used in this study. In the "BAU" projection, the carbon intensity of the global electricity generation is only decreasing slowly in the next decades until 2050. In the "NZE" (Net Zero Emissions) projection,

the carbon intensity is decreasing much faster. Both projections are based on IEA [76] scenarios. The “BAU” used in this study is based on IEA’s “STEPS” (Stated Policies) scenario, which provides a more conservative development for the future. The IEA reports the shares of the energy sources used for electricity generation on average for 2030 and 2050. Based on this together with the GHG factors from Table S10, the GHG factors for the future electricity generation in the “BAU” projection can be calculated whereby years in between are linearly interpolated. The “NZE” projection in this study is based on the eponymous scenario defined by the IEA, the “Net Zero Emissions by 2050” scenario. In this more optimistic scenario, the global temperature increase until 2050 would be limited to 1,5 °C. The carbon intensity of electricity generation would decrease to 138 $g_{CO2\text{-}eq.}$/kWh and to (or even below) zero in 2040. For the years 2020 to 2030 linearly interpolation is used. The resulting developments in both projections of the carbon intensity or GHG emissions for the global electricity generation are visualised in Figure S32 together with the historic data.

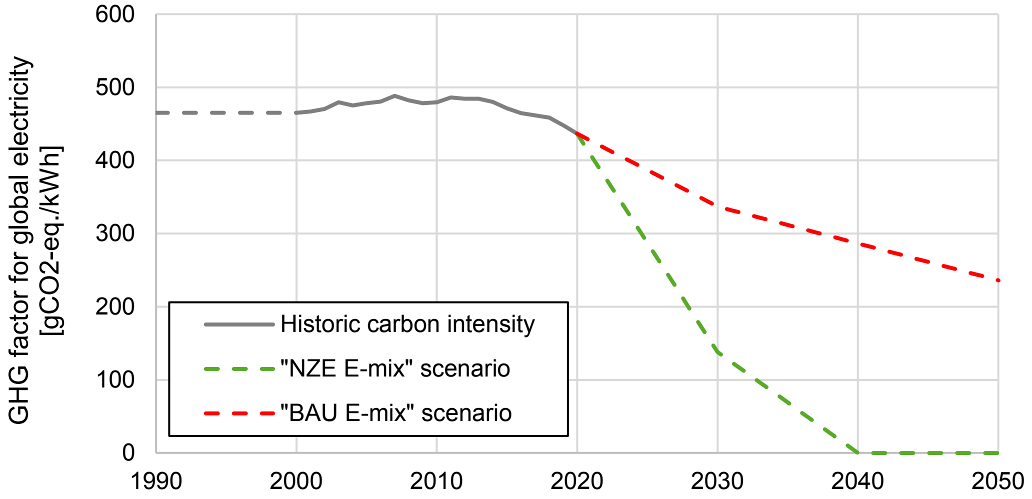

Figure S32: GHG emission factors for electricity usage in melting furnaces (E-mix projections)

Overview of the key parameters in the secondary melting technology system

An overview of the key parameters of the secondary melting technology system, which are presented in this chapter and are changed in future developments, is presented in Table S18.

Table S18: Overview of the key parameters in the smelter technology system

Parameter	Parameter choice	Description
Furnace lifetime	Low lifetime	20 years mean lifetime with 7 years standard deviation (30 % of mean value)
	High lifetime	30 years mean lifetime with 10 years standard deviation (30 % of mean value)
Market penetration of alternative heating technologies	BAU	Used technology in melting furnaces is not changed and specific energy demand not improved
	Electrification	Only electric heated melting furnaces (induction) is used in new production capacity from 2023 onwards
	Electrification and hydrogen	Only electric heated melting furnaces from 2023 - 2029; 50% / 50% electric and hydrogen heated melting furnaces from 2030 onwards
Specific energy demand	BAU	Used technology in melting furnaces is not changed and specific energy demand not improved
	Improved efficiency	Same furnace types as today, but specific energy demand decreases linearly to today's BAT (lowest energy demand for respective furnace type)
Electricity-mix (E-mix)	BAU	E-mix (global average) follows IEA's [76] STEPS (Stated Policies) scenario (most conservative development) to 2050
	NZE	Global E-mix follows IEA's [76] NZE (Net Zero Emissions) scenario to 2050 (carbon intensity of electricity generation decreases to zero in 2040)

A.4 – Carbon budget

The carbon budget used in this study is based on the IPCC global carbon budget to limit global warming to 1.5 °C and 2 °C, and its allocation to the aluminium sector. The IPCC [77] reports 420 $Gt_{CO2\text{-}eq.}$ as a budget to limit global warming to 1.5 °C and 1170 $Gt_{CO2\text{-}eq.}$ for 2 °C (67% likelihood) starting from 2018. We allocate 1.5% of this budget to the aluminium sector, which is based on the approach of Teske et al. [78] who calculated this share based on an integrated assessment model. This leads to ~6.4 $Gt_{CO2\text{-}eq.}$ and ~17.9 $Gt_{CO2\text{-}eq.}$ as remaining 1.5 °C and 2 °C budgets for the aluminium sector.

To verify this share, we assumed that all sectors must contribute equally, based on their emissions in 2018, to stay within the budget. Using total GHG emissions of the aluminium sector reported by the IAI [79] and the total global GHG emissions in 2018 [80], leads to a share of 2% for the total aluminium sector. When only including the electrolysis and secondary melting, as in our study, one end up at 1.5%. Allocation the carbon budget in equal shares to each sector based on today's emissions can lead to misleading results since emission reductions in other sectors due to the use of aluminium are not included. However, this is more used to verify the previous assumption at this point. The approach of allocating the available carbon budget equally to each sector is also followed by Watari et al. [81].

In comparison, the IAI [82] uses 16.9 and 25.5 $Gt_{CO2\text{-}eq.}$ as 2018-2050 carbon budgets for their 1.5 °C and Beyond 2 °C Scenario (B2DS). However, their carbon budget is meant to be for the entire aluminium sector, including alumina refining, semi-production, transport, and other auxiliaries. Also, it is unclear if the IAI used a 50% or 2/3 likelihood to reach the corresponding global warming. Thus, it is hard to compare to our estimated carbon budget but lays in the same magnitude.

Supplementary information - B

- Additional figures and results

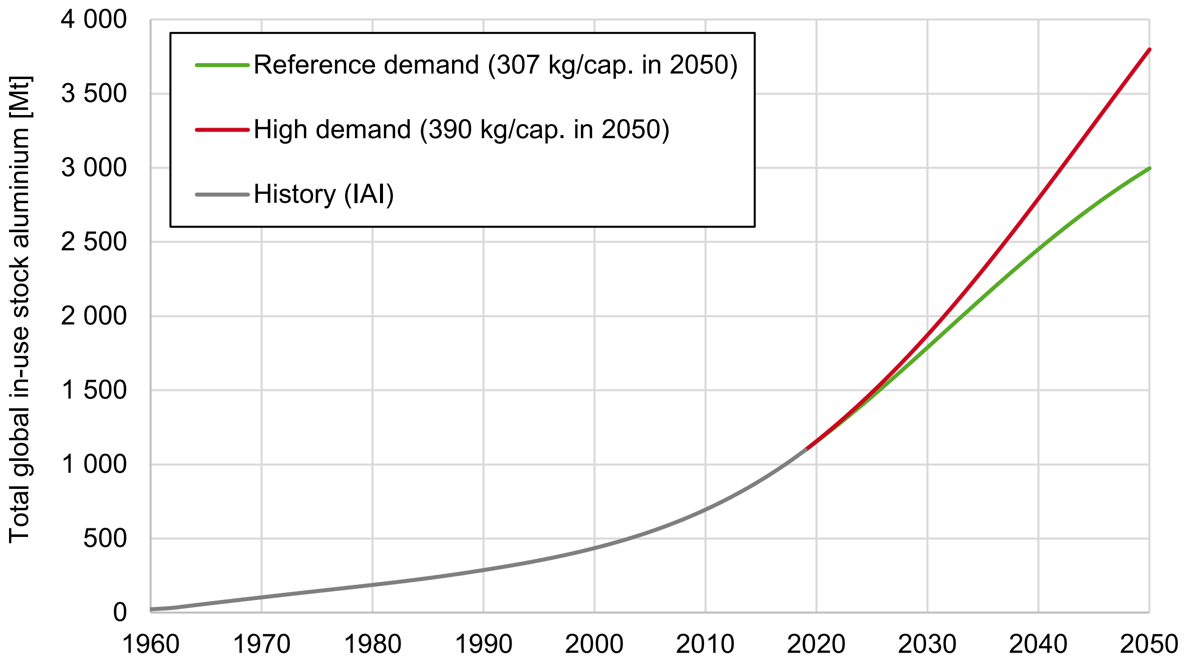

Figure S33: Development of total global in-use stock of aluminium under different in-use stock projections

B.1 – Global aluminium cycle – Additional results

Here, additional Sankey figures are shown for 2020 as a starting point again and 2050 in a reference demand and high demand scenario (for constant and increasing EOL collection rates and processing yields). This is followed by annual numbers for the primary and secondary aluminium production for 2020, 2030, 2040 and 2050.

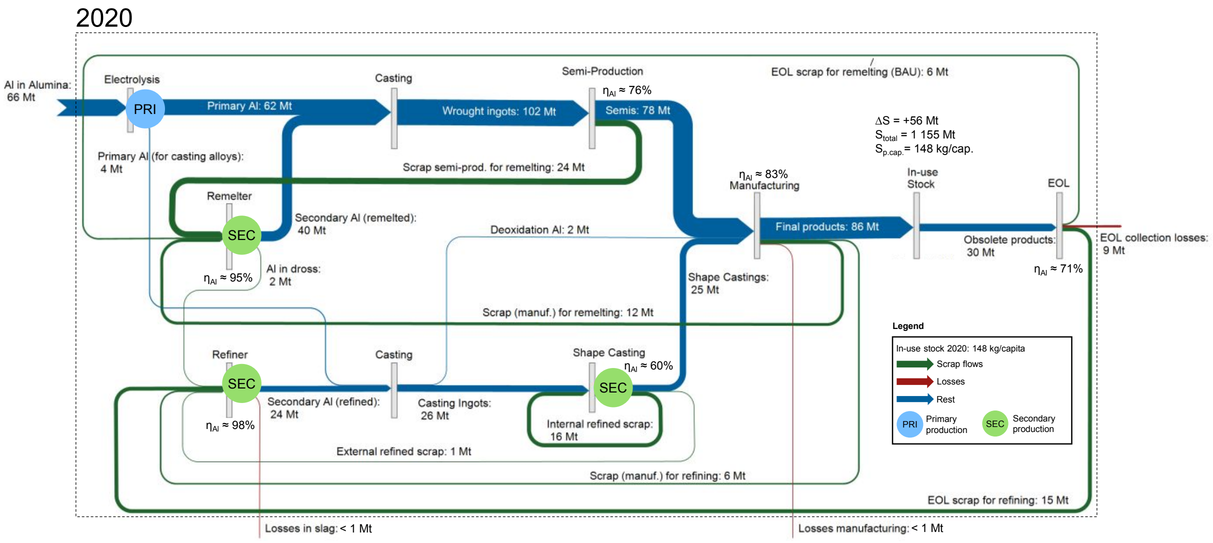

Figure S34: Global aluminium cycle in 2020.

Flows are shown for aluminium content only. Scrap flows are shown in green, losses in red and all other flows, such as liquid metal and (semi-) products, in blue. Semi-product and final product flows are further differentiated into 9 semi-product categories and 12 final product categories.

2050 – Reference demand & business-as-usual ("S0")

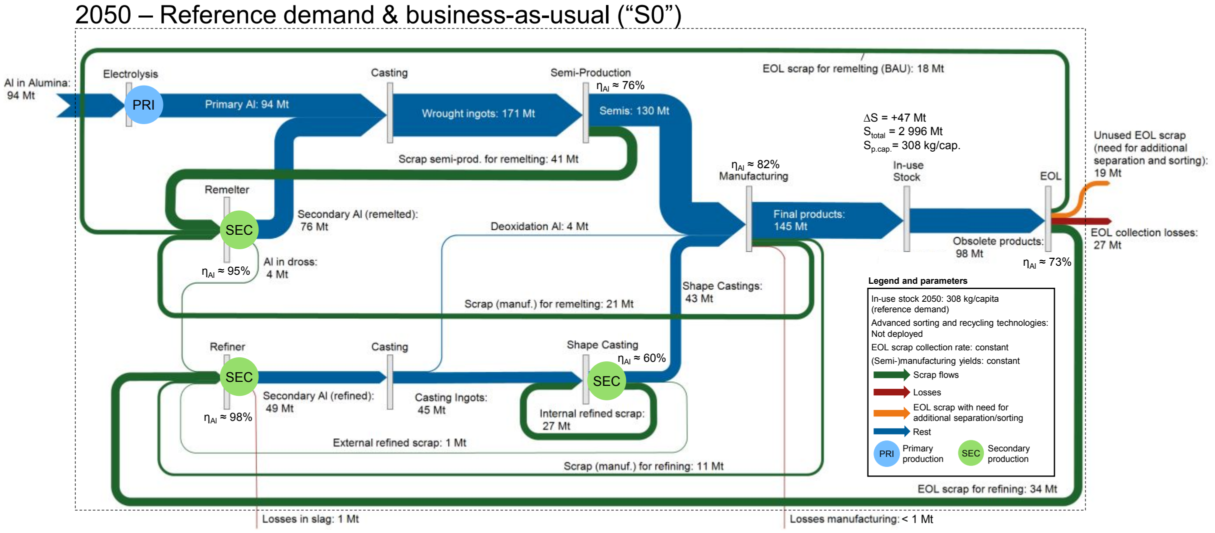

Figure S35: Global aluminium cycle in 2050 (reference demand, without advanced sorting and recycling technologies, constant, EOL scrap collection rates and yields) (scenario "S0")

2050 – Reference demand & advanced sorting and recycling technologies ("S1")

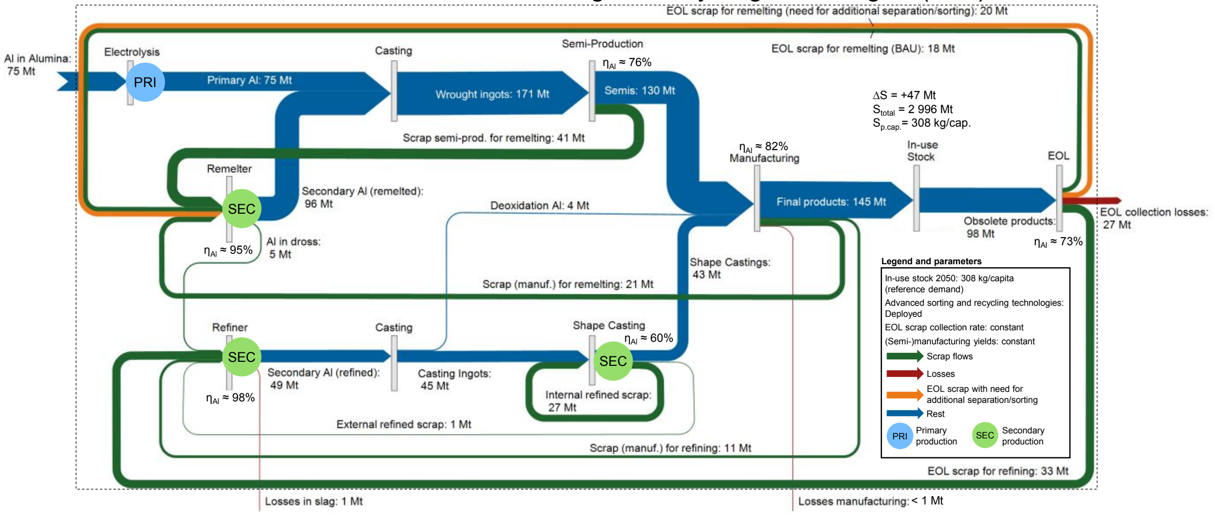

Figure S36: Global aluminium cycle in 2050 (reference demand, with advanced sorting and recycling technologies, constant, EOL scrap collection rates and yields) (scenario "S1")

2050 – High demand & business-as usual (“S0”)

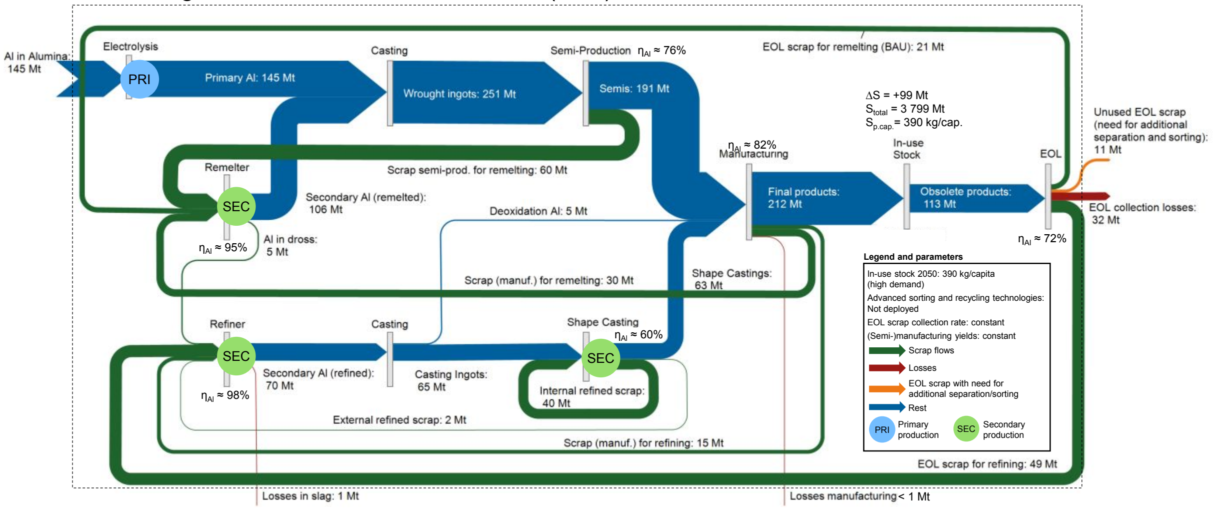

Figure S37: Global aluminium cycle in 2050 (high demand, without advanced sorting and recycling technologies, constant, EOL scrap collection rates and yields) (scenario “S0”)

2050 – High demand & advanced sorting and recycling technologies (“S1”)

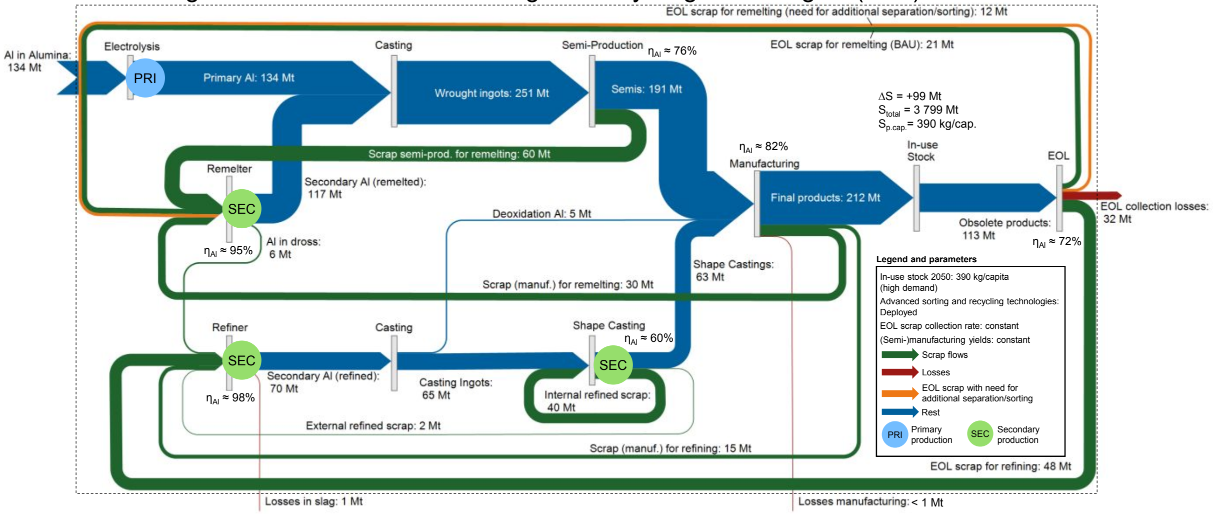

Figure S38: Global aluminium cycle in 2050 (high demand, with advanced sorting and recycling technologies, constant, EOL scrap collection rates and yields) (scenario “S1”)

2050 – Reference demand & advanced sorting and recycling + increasing EOL scrap collection rate & yields (“S3”)

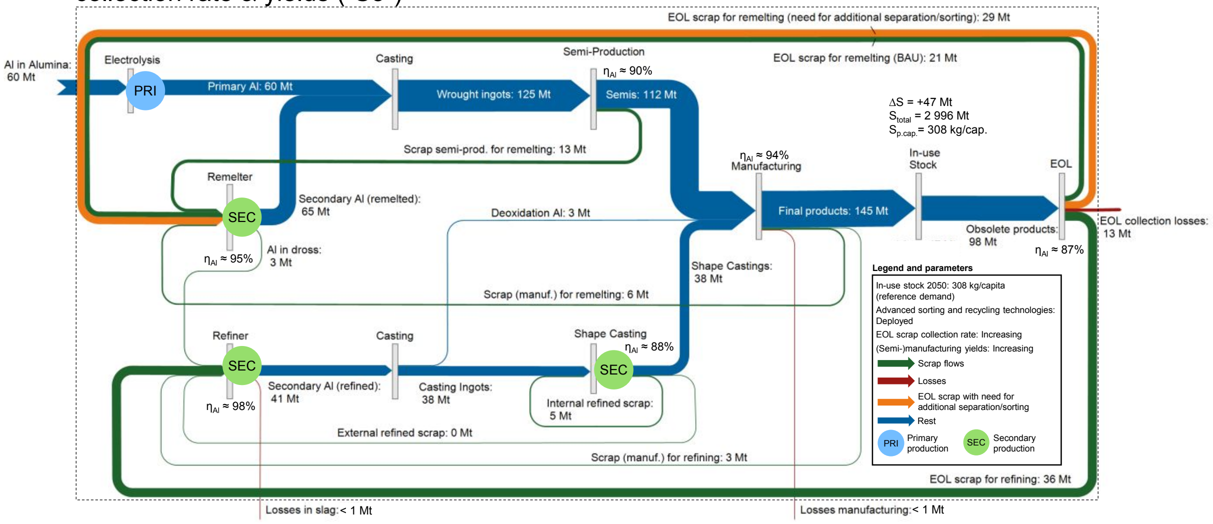

Figure S39: Global aluminium cycle in 2050 (reference demand, with advanced sorting and recycling technologies, increasing EOL scrap collection rates and yields) (scenario “S3”)

2050 – High demand & advanced sorting and recycling + increasing EOL scrap collection rate & yields (“S3”)

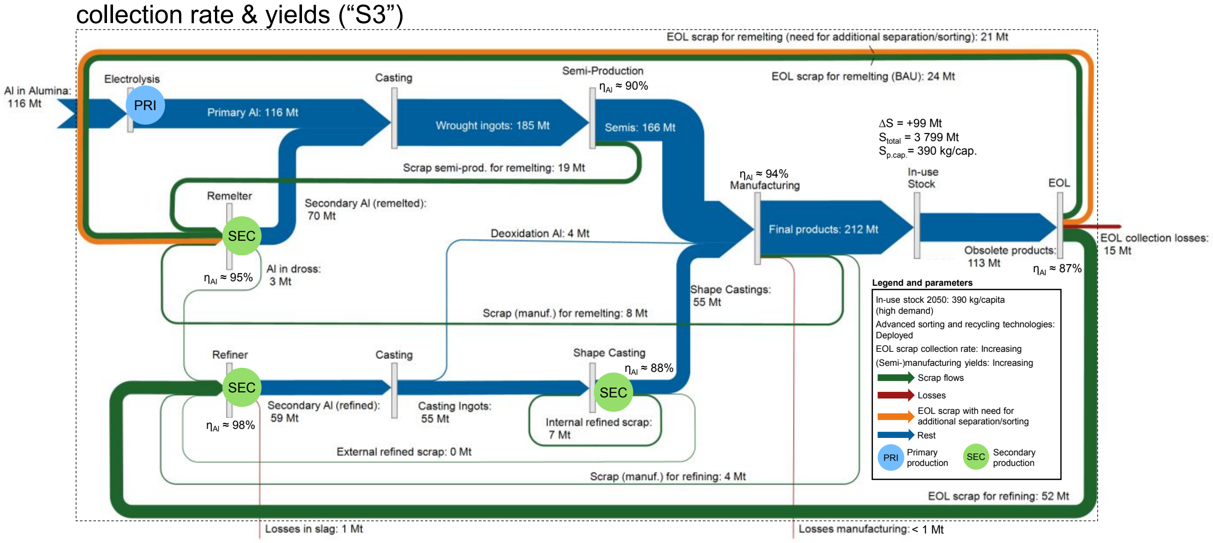

Figure S40: Global aluminium cycle in 2050 (high demand, with advanced sorting and recycling technologies, increasing EOL scrap collection rates and yields) (scenario “S3”)

Table S19: Primary and secondary aluminium production for reference demand and ***no advanced sorting and recycling technologies***

EOL collection rate		Primary Al [Mt/a]				Secondary Al [Mt/a]							
		Constant		Increase		Constant				Increase			
**Yields**		Constant	Increase	Constant	Increase	Constant		Increase		Constant		Increase	
**Source 2nd Al**		-	-	-	-	New	EOL	New	EOL	New	EOL	New	EOL
**Year**	2020	66	66	66	66	59	21	59	21	59	21	59	21
	2030	83	82	81	80	79	34	65	34	79	35	65	36
	2040	92	89	90	88	94	44	46	47	94	46	46	48
	2050	94	90	92	88	102	52	27	56	102	54	27	58

Table S20: Primary and secondary aluminium production for reference demand and ***advanced sorting and recycling technologies***

EOL collection rate		Primary Al [Mt/a]				Secondary Al [Mt/a]							
		Constant		Increase		Constant				Increase			
**Yields**		Constant	Increase	Constant	Increase	Constant		Increase		Constant		Increase	
**Source 2nd Al**		-	-	-	-	New	EOL	New	EOL	New	EOL	New	EOL
**Year**	2020	66	66	66	66	59	21	59	21	59	21	59	21
	2030	83	82	80	80	79	34	65	34	79	36	65	36
	2040	85	84	75	75	94	51	46	51	94	61	46	61
	2050	75	75	61	60	102	71	27	71	102	86	27	86

Table S21: Primary and secondary aluminium production for high demand and ***no advanced sorting and recycling technologies***

EOL collection rate		Primary Al [Mt/a]				Secondary Al [Mt/a]							
		Constant		Increase		Constant				Increase			
**Yields**		Constant	Increase	Constant	Increase	Constant		Increase		Constant		Increase	
**Source 2nd Al**		-	-	-	-	New	EOL	New	EOL	New	EOL	New	EOL
**Year**	2020	69	69	69	69	61	21	61	21	61	21	61	21
	2030	98	97	95	95	89	34	73	34	89	37	73	37
	2040	123	122	121	118	121	54	60	54	121	56	60	59
	2050	145	139	142	136	147	70	39	75	147	73	39	78

Table S22: Primary and secondary aluminium production for high demand and ***advanced sorting and recycling technologies***

**EOL collection rate**		**Primary Al [Mt/a]**				**Secondary Al [Mt/a]**							
		Constant		Increase		Constant				Increase			
**Yields**		Constant	Increase	Constant	Increase	Constant		Increase		Constant		Increase	
**Source 2nd Al**		-	-	-	-	New	EOL	New	EOL	New	EOL	New	EOL
**Year**	2020	69	69	69	69	61	21	61	21	61	21	61	21
	2030	97	97	95	95	89	34	73	34	89	37	73	37
	2040	123	122	112	112	121	54	60	54	121	65	60	65
	2050	134	133	117	116	147	81	39	81	147	98	39	98

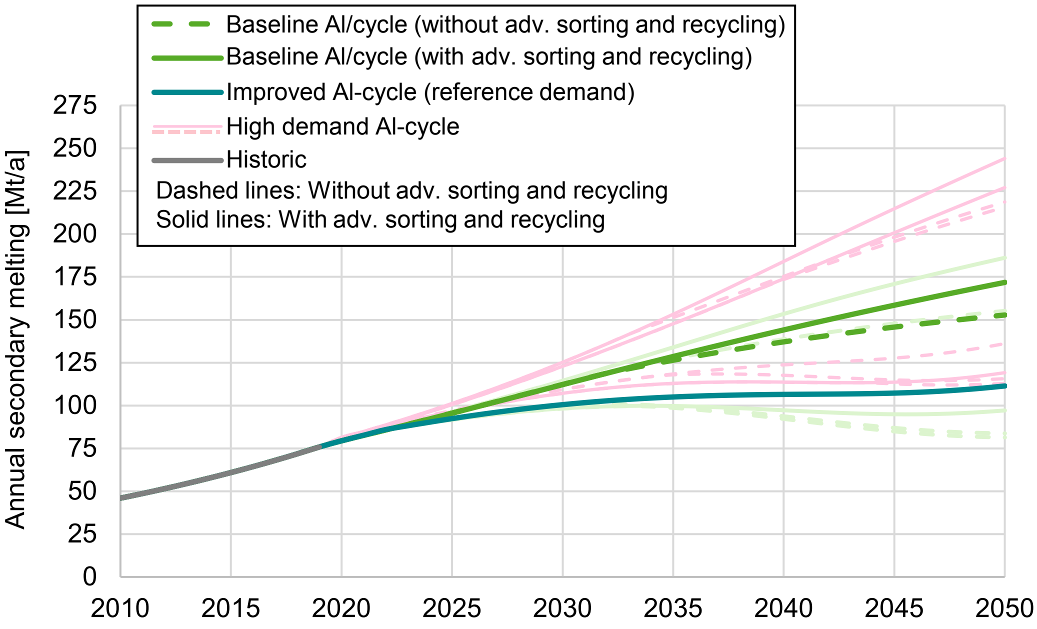

Figure S41: Future secondary aluminium production under different demand and parameter scenarios (new and old scrap together)

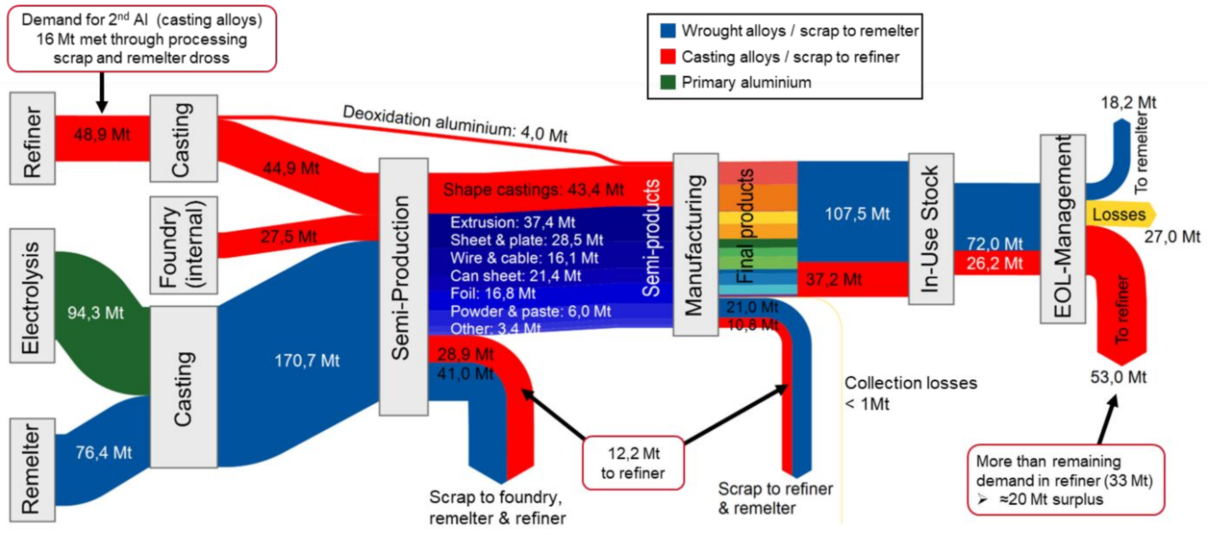

Figure S42: Global aluminium cycle for 2050 (reference demand) with wrought and casting alloy flows.

The wrought alloys and scrap flows which are entering the remelter process are shown in blue, casting alloys and scrap flows entering the refiner in red. The primary aluminium production is shown in green. This figure points out the challenge behind the scrap surplus, as the EOL scrap flow which would be recycled in the refiners to casting alloys (53 Mt) exceeds the demand in the refiner. In year 2050 there would be a demand for casting alloys from refiner of 48,9 Mt, which is not enough to take up all the EOL scrap for refining (as it would be today). Thus, the remaining scrap would be sorted and separated into wrought alloys and is assumed to be recycled in the remelter in this study when we assume that advanced sorting and recycling technologies are developed.

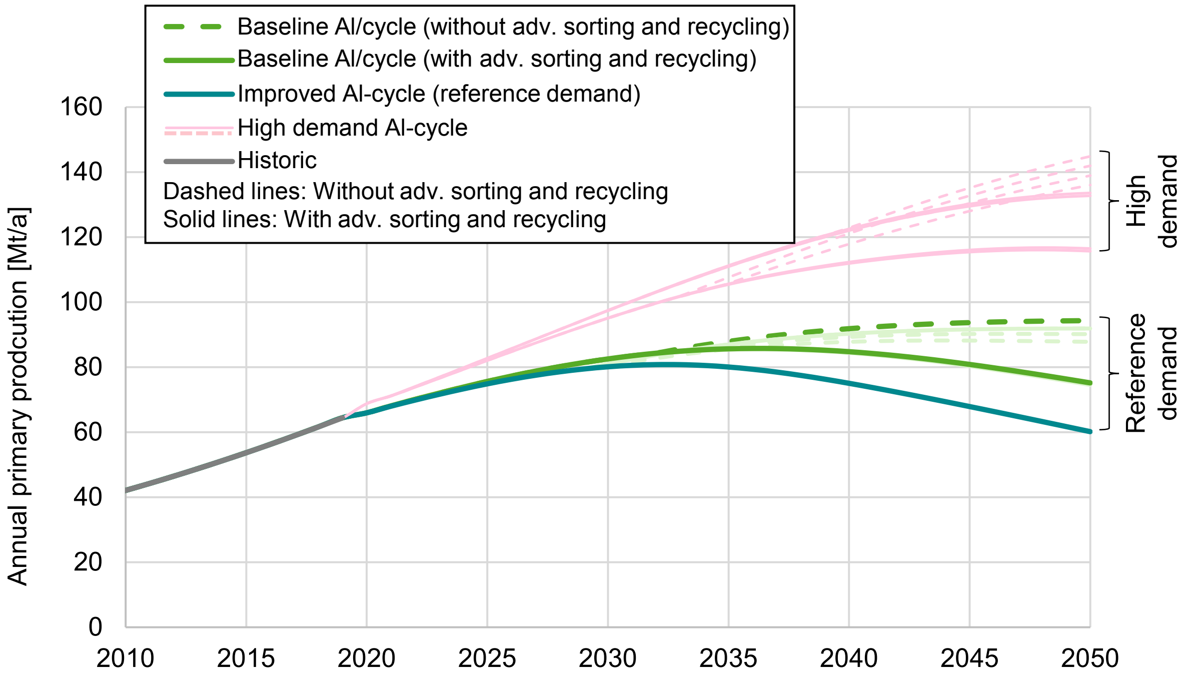

Figure S43: Future primary aluminium production under different demand and parameter scenarios

B.2 – Total stocks of technology (production capacity)

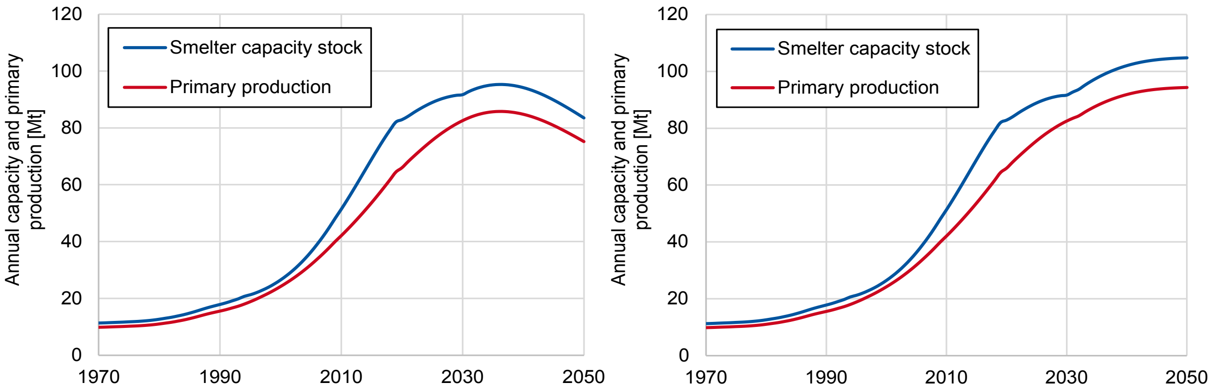

Figure S44: Development of total global aluminium smelter stock (reference demand)

*for "with advanced sorting and recycling technologies" (left) and "without advanced sorting and recycling technologies" (right) for **reference demand.** For assumptions on the utilization rate of smelters, see Figure S13.*

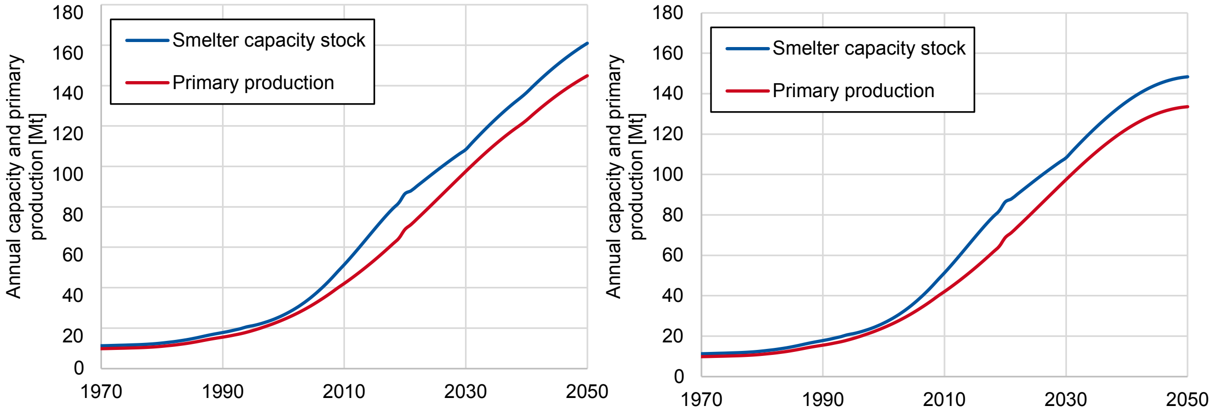

Figure S45: Development of total global aluminium smelter stock (high demand)

*for "with advanced sorting and recycling technologies" (left) and "without advanced sorting and recycling technologies" (right) for **high demand.** For assumptions on the utilization rate of smelters, see Figure S13.*

B.3 – Stock shares for smelters with inert anodes

Table S23: Development of smelter stocks with inert anodes (reference demand, constant EOL scrap collection rates and yields)

		Market penetration	Retrofitting	Smelter using inert anodes in year t			
				**2035**	**2040**	**2045**	**2050**
**Without advanced sorting and recycling**	**L40**	Low MPR (20-80%)	no RF	5%	14%	23%	33%
			1 $Mt_{annual\ capacity}$/yr	12%	25%	39%	53%
			2 $Mt_{annual\ capacity}$/yr	18%	36%	54%	73%
		High MPR (100%)	no RF	15%	27%	38%	50%
			1 $Mt_{annual\ capacity}$/yr	21%	38%	54%	70%
			2 $Mt_{annual\ capacity}$/yr	27%	49%	69%	90%
	**L50**	Low MPR (20-80%)	no RF	4%	11%	17%	24%
			1 $Mt_{annual\ capacity}$/yr	10%	21%	32%	44%
			2 $Mt_{annual\ capacity}$/yr	17%	32%	48%	64%
		High MPR (100%)	no RF	12%	21%	29%	36%
			1 $Mt_{annual\ capacity}$/yr	18%	31%	44%	57%
			2 $Mt_{annual\ capacity}$/yr	24%	42%	59%	77%
**With advanced sorting and recycling**	**L40**	Low MPR (20-80%)	no RF	4%	10%	16%	23%
			1 $Mt_{annual\ capacity}$/yr	11%	22%	34%	48%
			2 $Mt_{annual\ capacity}$/yr	17%	33%	52%	73%
		High MPR (100%)	no RF	12%	21%	29%	38%
			1 $Mt_{annual\ capacity}$/yr	19%	33%	47%	63%
			2 $Mt_{annual\ capacity}$/yr	25%	44%	65%	88%
	**L50**	Low MPR (20-80%)	no RF	3%	6%	9%	11%
			1 $Mt_{annual\ capacity}$/yr	10%	18%	27%	36%
			2 $Mt_{annual\ capacity}$/yr	16%	30%	45%	62%
		High MPR (100%)	no RF	9%	14%	18%	21%
			1 $Mt_{annual\ capacity}$/yr	16%	26%	35%	46%
			2 $Mt_{annual\ capacity}$/yr	22%	38%	53%	71%

*MPR: Market-penetration rate (inflow share); RF: Retrofitting

Table S24: Development of smelter stocks with inert anodes (high demand, constant EOL scrap collection rates and yields)

		Market penetration	Retrofitting	Smelter using inert anodes in year t			
				**2035**	**2040**	**2045**	**2050**
**Without advanced sorting and recycling**	**L40**	Low MPR (20-80%)	no RF	7%	18%	29%	39%
			1 $Mt_{annual\ capacity}$/yr	12%	26%	40%	52%
			2 $Mt_{annual\ capacity}$/yr	17%	34%	50%	65%
		High MPR (100%)	no RF	21%	35%	48%	59%
			1 $Mt_{annual\ capacity}$/yr	25%	43%	58%	72%
			2 $Mt_{annual\ capacity}$/yr	30%	51%	69%	85%
	**L50**	Low MPR (20-80%)	no RF	6%	15%	25%	32%
			1 $Mt_{annual\ capacity}$/yr	11%	23%	35%	45%
			2 $Mt_{annual\ capacity}$/yr	16%	31%	46%	58%
		High MPR (100%)	no RF	18%	30%	41%	50%
			1 $Mt_{annual\ capacity}$/yr	23%	38%	51%	63%
			2 $Mt_{annual\ capacity}$/yr	28%	46%	62%	76%
**With advanced sorting and recycling**	**L40**	Low MPR (20-80%)	no RF	7%	17%	27%	36%
			1 $Mt_{annual\ capacity}$/yr	12%	25%	38%	50%
			2 $Mt_{annual\ capacity}$/yr	17%	34%	49%	64%
		High MPR (100%)	no RF	21%	34%	46%	56%
			1 $Mt_{annual\ capacity}$/yr	25%	43%	57%	70%
			2 $Mt_{annual\ capacity}$/yr	30%	51%	68%	84%
	**L50**	Low MPR (20-80%)	no RF	6%	15%	22%	29%
			1 $Mt_{annual\ capacity}$/yr	11%	23%	34%	43%
			2 $Mt_{annual\ capacity}$/yr	16%	31%	45%	57%
		High MPR (100%)	no RF	18%	30%	38%	46%
			1 $Mt_{annual\ capacity}$/yr	23%	38%	49%	60%
			2 $Mt_{annual\ capacity}$/yr	28%	46%	61%	74%

*MPR: Market-penetration rate (inflow share); RF: Retrofitting

B.4 – Direct and indirect GHG emission from smelters

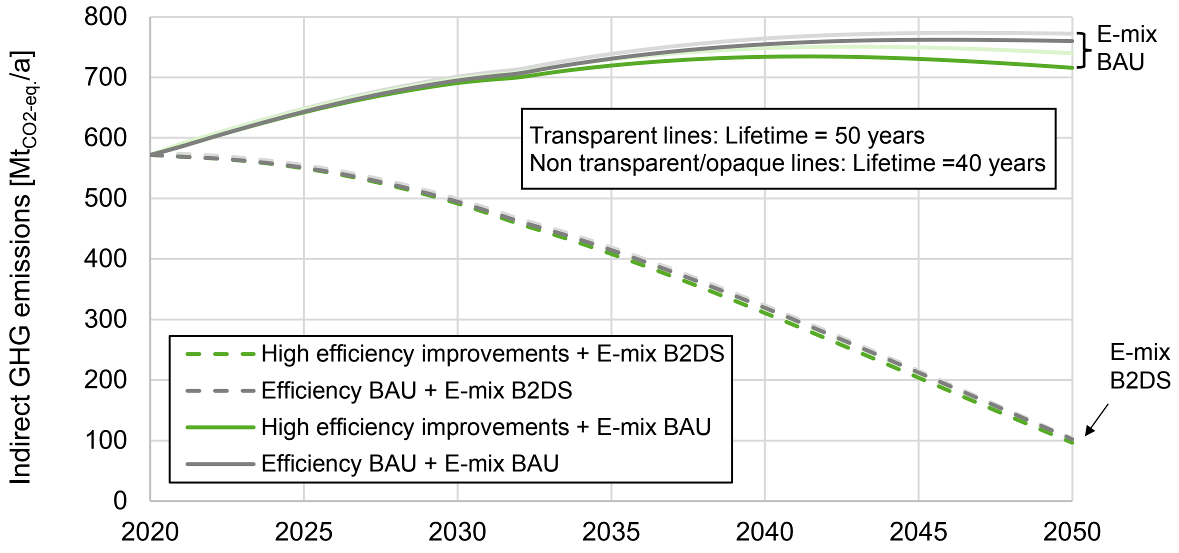

Figure S46: Indirect global GHG emissions of smelters without advanced sorting and recycling technologies (“reference demand”)

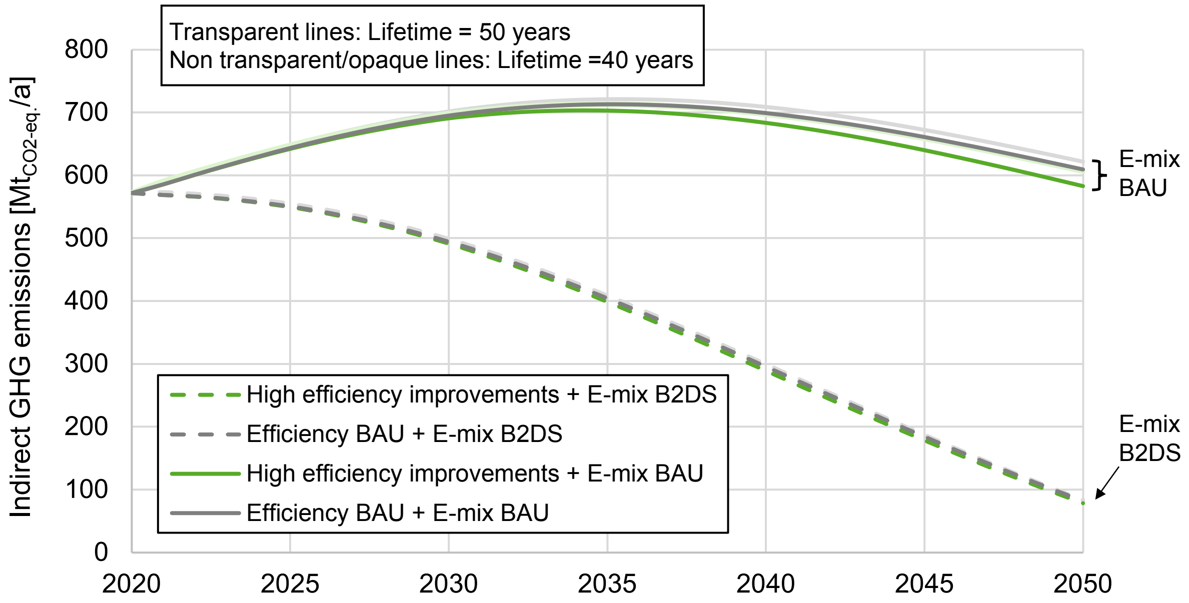

Figure S47: Indirect global GHG emissions of smelters with advanced sorting and recycling technologies (“reference demand”)

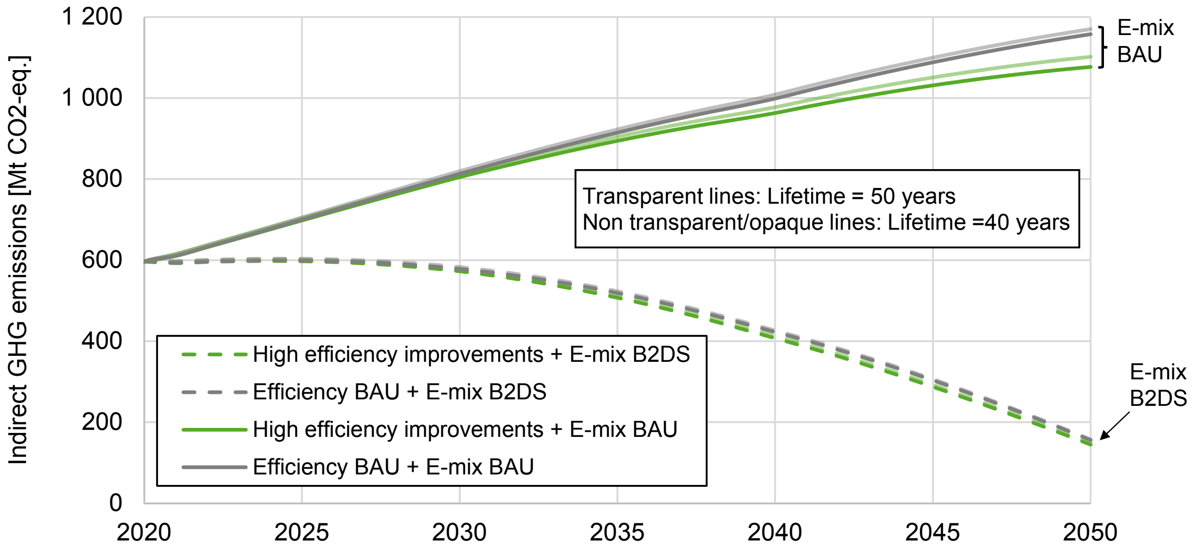

Figure S48: Indirect global GHG emissions of smelters without advanced sorting and recycling technologies ("high demand")

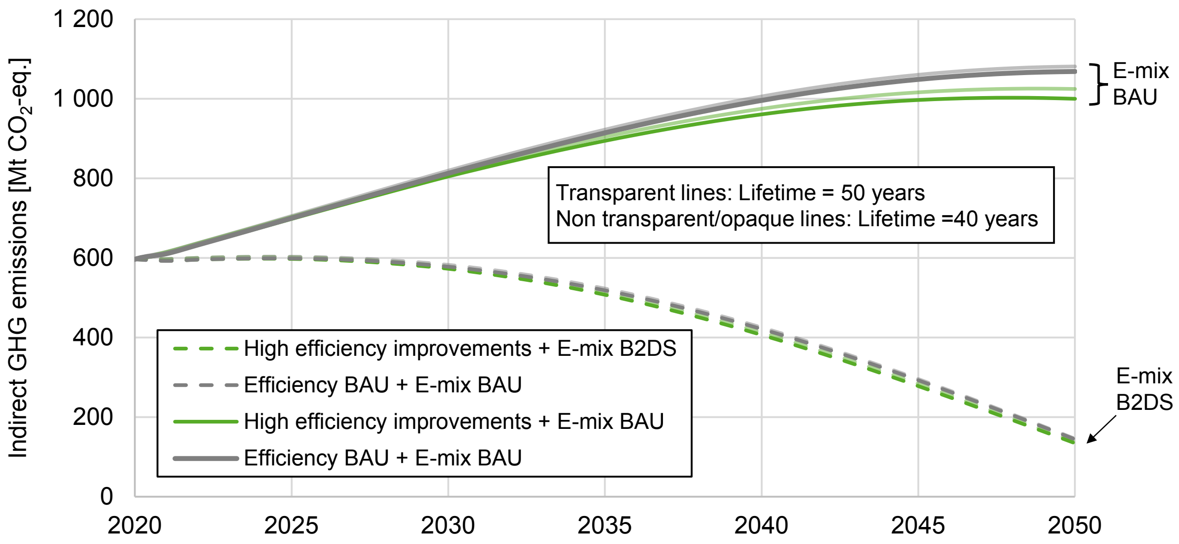

Figure S49: Indirect global GHG emissions of smelters with advanced sorting and recycling technologies ("high demand")

B.5 – Stock shares for melting furnaces in future

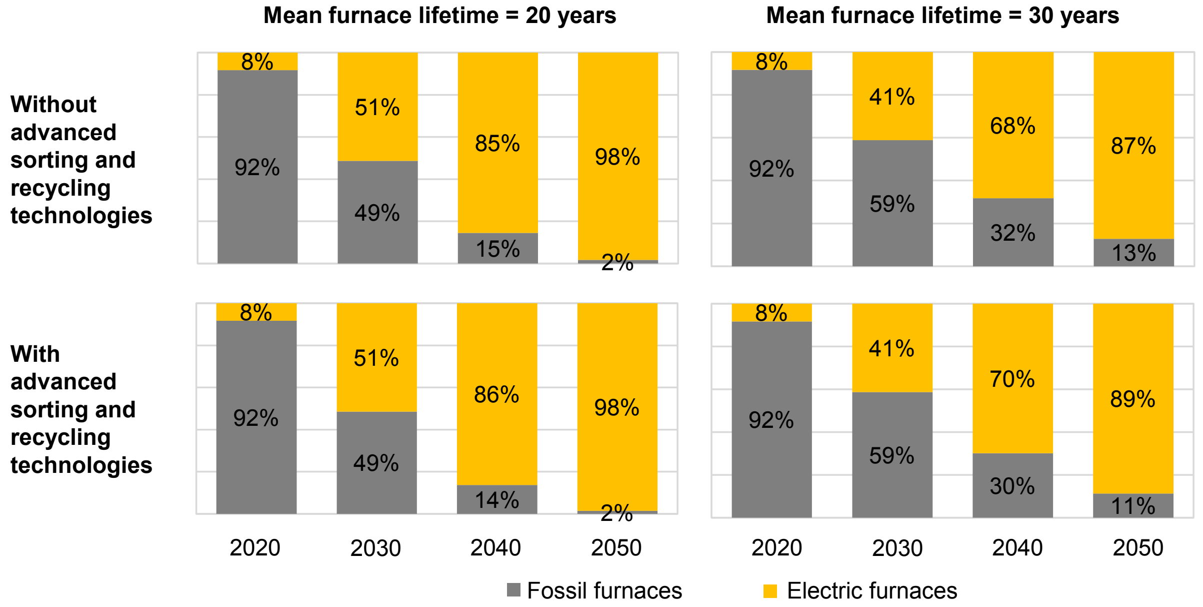

*Figure S50: Development of melting furnace stock with "high electrification" scenario (100% of melting capacity inflows are using electrically heated furnaces from 2023) for **reference demand** scenario and constant EOL scrap collection rates and yields*

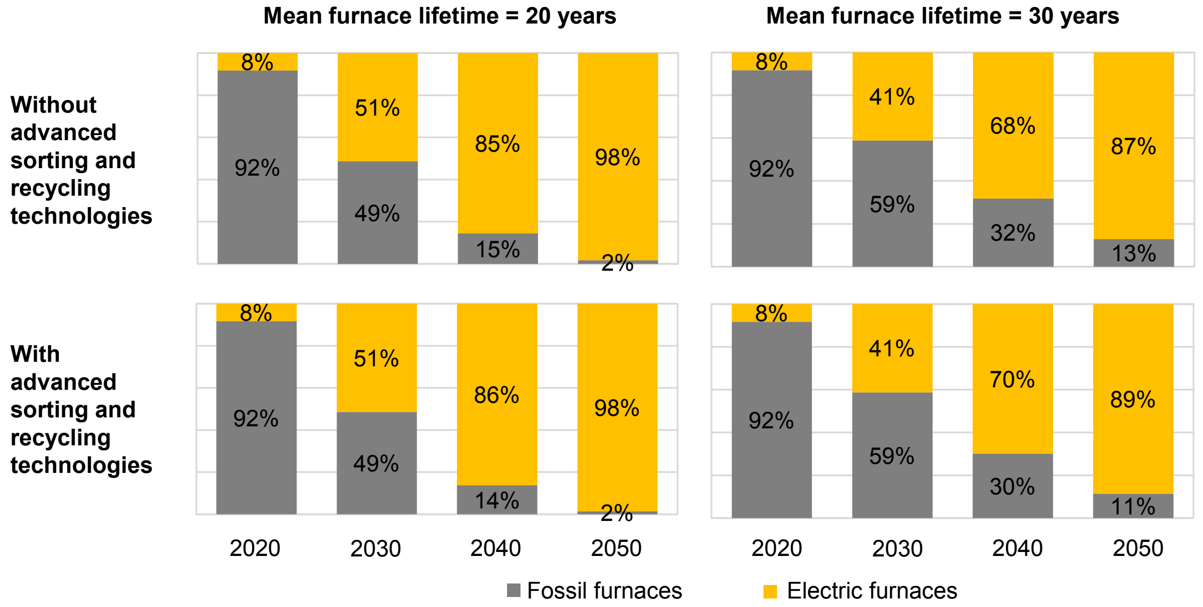

*Figure S51: Development of melting furnace stock with "high electrification" (100% of melting capacity inflows are using electrically heated furnaces from 2023) for **high demand** scenario and constant EOL scrap collection rates and yields*

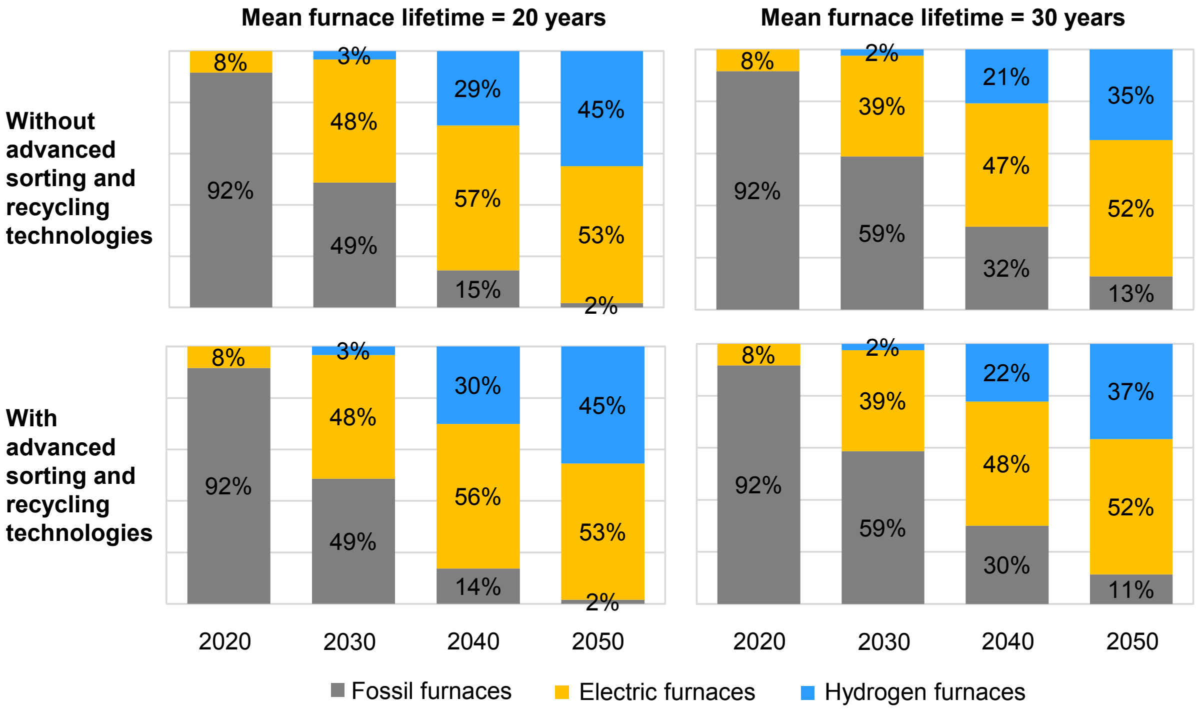

*Figure S52: Development of melting furnace stock with "electrification + hydrogen" (until 2029 same as "high electrification". From 2030 50% hydrogen and 50% electrically heated furnaces are used in capacity inflows) for **reference demand** scenario and constant EOL scrap collection rates and yields*

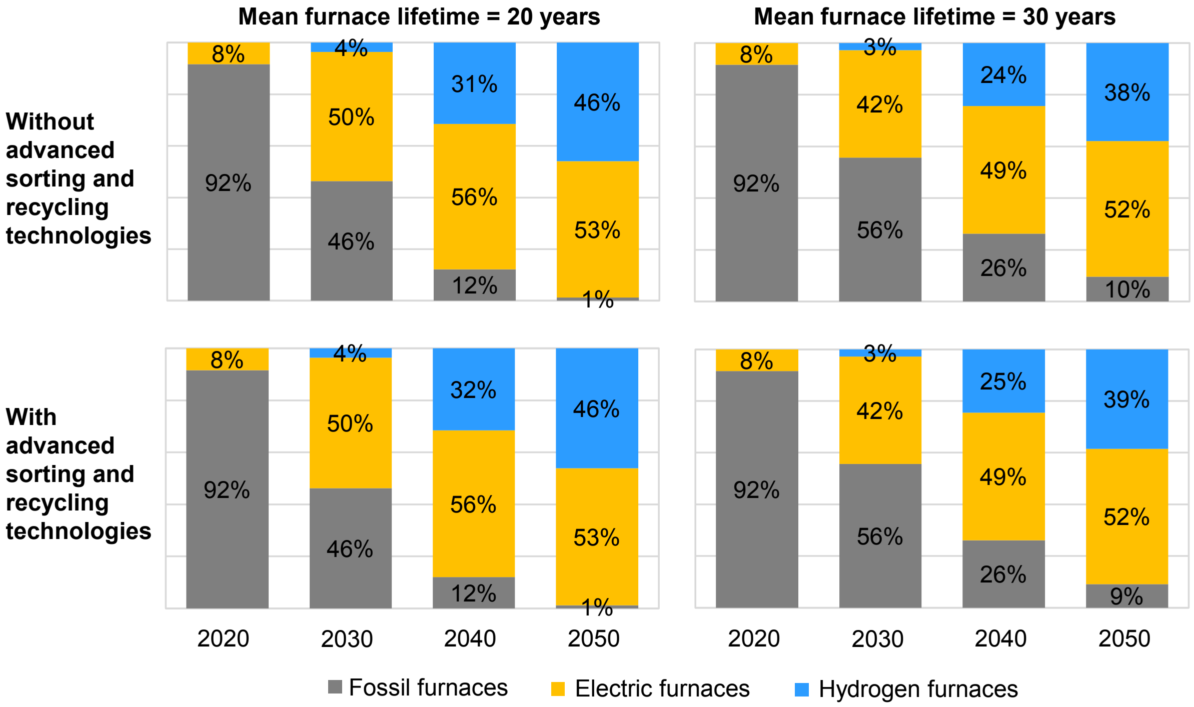

*Figure S53: Development of melting furnace stock with "electrification + hydrogen" (until 2029 same as "high electrification". From 2030 50% hydrogen and 50% electrically heated furnaces are used in capacity inflows) for **high demand** scenario and constant EOL scrap collection rates and yields*

B.6 – GHG emissions from melting

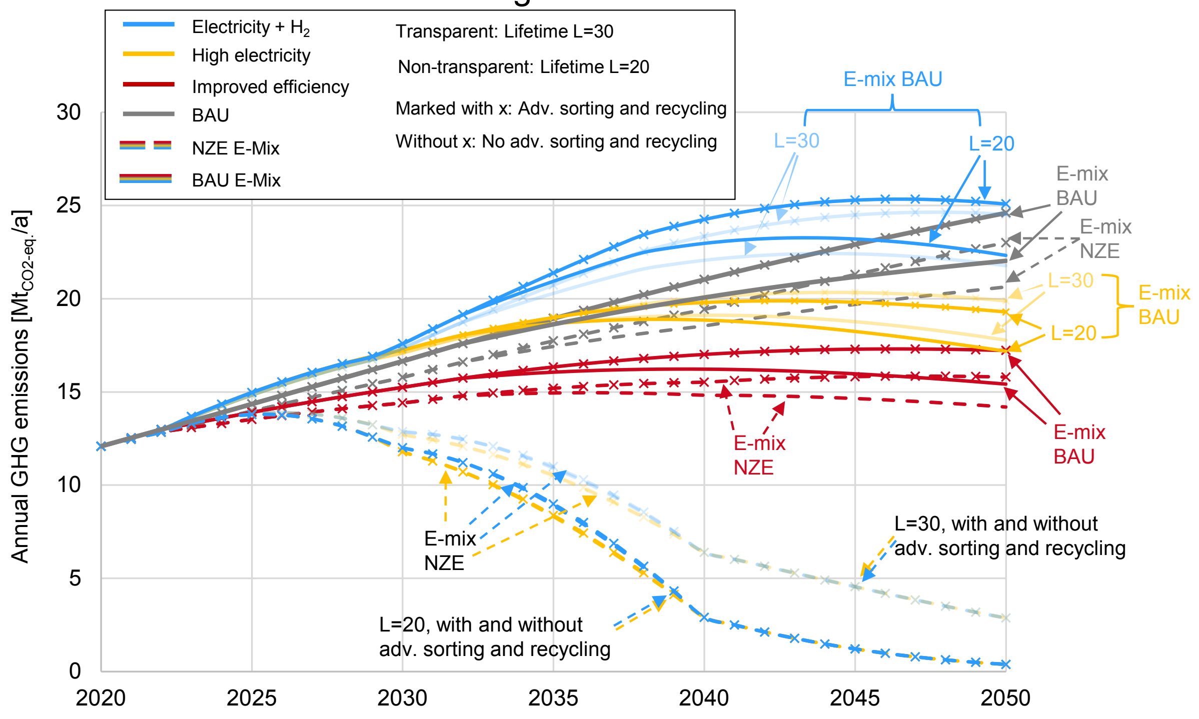

Figure S54: Annual global GHG emissions of secondary aluminium melting for different technology projections and furnace lifetimes (reference demand)

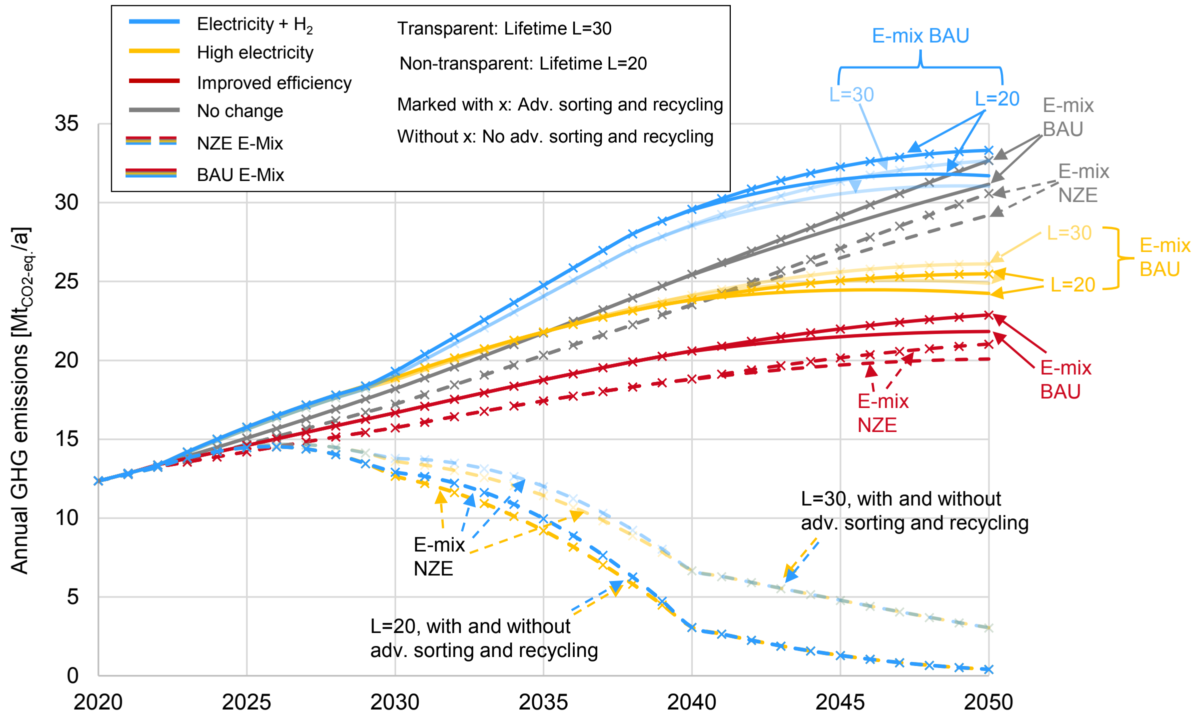

Figure S55: Annual global GHG emissions of secondary aluminium melting for different technology projections and furnace lifetimes (high demand)

B.7 – Contour figure of specific direct GHG emissions of the primary aluminium production

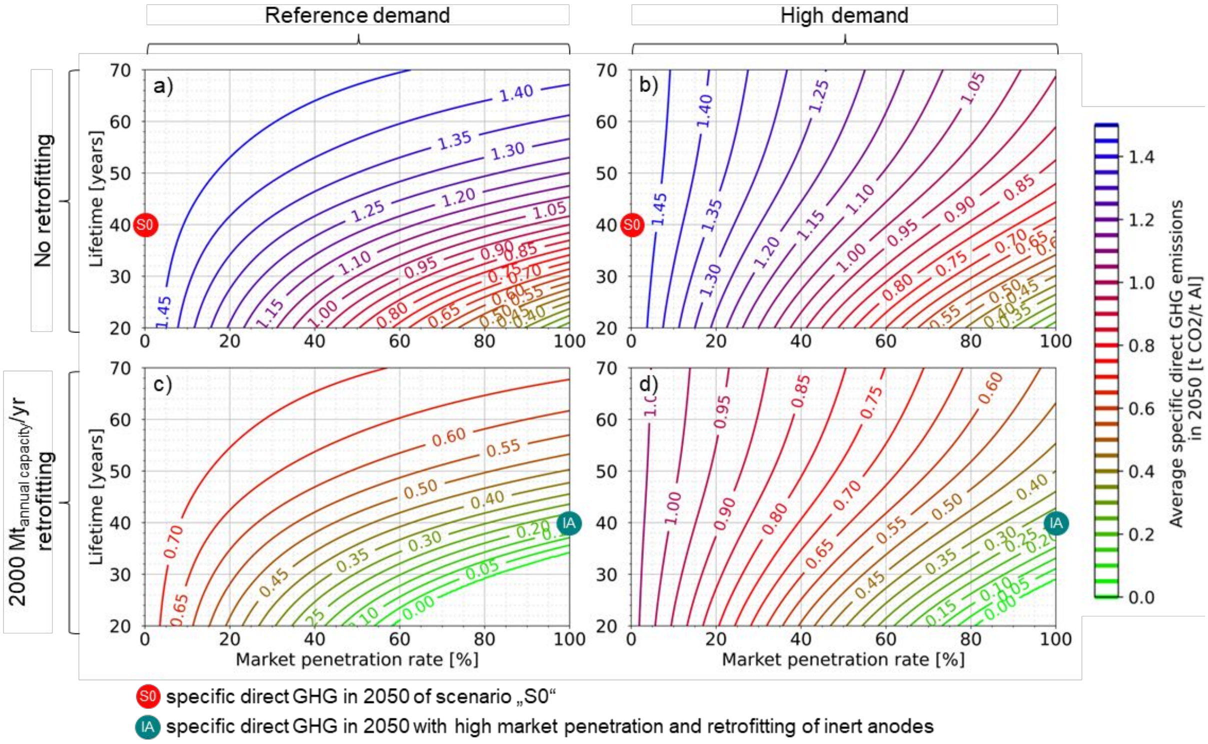

Figure S56: Sensitivity analysis of specific direct emissions of the primary aluminium production in 2050 (with advanced sorting and recycling technologies)

depending on smelter lifetime and market penetration rate (MPR) of inert anodes. Here, we show scenarios based on the primary demand when advanced sorting and recycling technologies are implemented (contrary to figure 5 in the manuscript) to mark the scenarios “S1” and “S2” in the figure. Emissions are shown as contour lines for two demand and retrofitting scenarios, where each line represents one solution for specific direct emissions in 2050. MPR is used as a constant percentage of inert anodes used in new built smelters each year from 2030. Smelter lifetimes are normally distributed and used over the entire model timeframe.

References

(1) European Aluminium. *Environmental profile report:Life-Cycle inventory data for aluminium production and transformation processes in Europe*. https://european-aluminium.eu/blog/environmental-profile-reports/ (accessed 2023-05-19).

(2) Billy, R. G.; Monnier, L.; Nybakke, E.; Isaksen, M.; Müller, D. B. Systemic Approaches for Emission Reduction in Industrial Plants Based on Physical Accounting: Example for an Aluminum Smelter. *Environmental science & technology* **2022**, *56* (3), 1973–1982. DOI: 10.1021/acs.est.1c05681. Published Online: Jan. 19, 2022.

(3) Holywell, G.; Breault, R. An Overview of Useful Methods to Treat, Recover, or Recycle Spent Potlining. *JOM* **2013**, *65* (11), 1441–1451. DOI: 10.1007/s11837-013-0769-y.

(4) Cusano, G.; Delgado Sancho, L.; Farrell, F.; Rodrigo Gonzalo, M.; Roudier, S. *Best available techniques (BAT) reference document for the non-ferrous metals – Industrial Emissions Directive 2010/75/EU (integrated pollution prevention and control) industries. Publications Office of the European Union*. https://data.europa.eu/doi/10.2760/8224 (accessed 2024-05-08).

(5) Cullen, J. M.; Allwood, J. M. Mapping the global flow of aluminum: from liquid aluminum to end-use goods. *Environmental science & technology* **2013**, *47* (7), 3057–3064. DOI: 10.1021/es304256s. Published Online: Mar. 11, 2013.

(6) Liu, G.; Bangs, C. E.; Müller, D. B. Stock dynamics and emission pathways of the global aluminium cycle. *Nature Clim Change* **2013**, *3* (4), 338–342. DOI: 10.1038/nclimate1698.

(7) Boin, U. M. J.; Bertram, M. Melting standardized aluminum scrap: A mass balance model for Europe. *The Journal of The Minerals, Metals & Materials Society* **2005**, *57* (8), 26–33. DOI: 10.1007/s11837-005-0164-4.

(8) International Aluminium Institute. *Opportunities For Aluminium In A Post-Covid Economy*. https://international-aluminium.org/resource/opportunities-for-aluminium-in-a-post-covid-economy/?_thumbnail_id=6715 (accessed 2022-08-24).

(9) International Aluminium Institute. *CM Group: Global Megatrends and Regional and Market Sector Growth Outlook for Aluminium Demand*. https://international-aluminium.org/resource/cm-group-global-megatrends-and-regional-and-market-sector-growth-outlook-for-aluminium-demand/ (accessed 2022-08-24).

(10) United Nations. *Department of Economic and Social Affairs, Population Division: World Population Prospects 2019, Online Edition. Rev. 1.* https://population.un.org/wpp/Download/Standard/Population/.

(11) International Aluminium Institute. *Public Access - International Aluminium Institute*. https://alucycle.international-aluminium.org/public-access/ (accessed 2022-03-16).

(12) Pauliuk, S. *ODYM - Open Dynamic Material Systems Model*; Industrial Ecology, 2018. https://github.com/IndEcol/ODYM.

(13) Pauliuk, S.; Wang, T.; Müller, D. B. Moving toward the circular economy: the role of stocks in the Chinese steel cycle. *Environmental science & technology* **2012**, *46* (1), 148–154. DOI: 10.1021/es201904c. Published Online: Dec. 12, 2011.

(14) Müller, D. B. Stock dynamics for forecasting material flows—Case study for housing in The Netherlands. *Ecological Economics* **2006**, *59* (1), 142–156. DOI: 10.1016/j.ecolecon.2005.09.025.

(15) International Aluminium Institute. *Aluminium Recycling – Sustainability*. https://recycling.world-aluminium.org/review/sustainability/ (accessed 2022-08-31).

(16) European Aluminium. *Environmental profile report:Life-Cycle inventory data for aluminium production and transformation processes in Europe*. https://www.google.de/url?sa=t&rct=j&q=&esrc=s&source=web&cd=&ved=2ahUKEwiz9e2_sL75AhWei_0HHRfTCCMQFnoECA8QAQ&url=https%3A%2F%2Feuropean-

aluminium.eu%2Fmedia%2F3341%2Fenvironmental-profile-report-for-the-european-aluminium-industry.pdf&usg=AOvVaw1xi1Fd57SW2NWWYTFinW39.

(17) Bertram, M. *Internal communication: Secondary scrap flows of IAI's global aluminium cycle*, 2022.

(18) Silva, N. d., N. d'Souza, and M. Binder. *Life Cycle Impact Assessment of Aluminum Beverage Cans. PE Americas. 2010*. https://www.container-recycling.org/assets/pdfs/aluminum/LCA-2010-AluminumAssoc.pdf (accessed 2024-05-08).

(19) Hatayama, H.; Daigo, I.; Matsuno, Y.; Adachi, Y. Outlook of the world steel cycle based on the stock and flow dynamics. *Environmental science & technology* **2010**, *44* (16), 6457–6463. DOI: 10.1021/es100044n.

(20) Pauliuk, S.; Milford, R. L.; Müller, D. B.; Allwood, J. M. The steel scrap age. *Environmental science & technology* **2013**, *47* (7), 3448–3454. DOI: 10.1021/es303149z. Published Online: Mar. 7, 2013.

(21) Igarashi, Y.; Kakiuchi, E.; Daigo, I.; Matsuno, Y.; Adachi, Y. Estimation of Steel Consumption and Obsolete Scrap Generation in Japan and Asian Countries in the Future. *ISIJ Int.* **2008**, *48* (5), 696–704. DOI: 10.2355/isijinternational.48.696.

(22) Paraskevas, D.; Kellens, K.; Dewulf, W.; Duflou, J. R. Environmental modelling of aluminium recycling: a Life Cycle Assessment tool for sustainable metal management. *Journal of Cleaner Production* **2015**, *105*, 357–370. DOI: 10.1016/j.jclepro.2014.09.102.

(23) Hatayama, H.; Daigo, I.; Matsuno, Y.; Adachi, Y. Evolution of aluminum recycling initiated by the introduction of next-generation vehicles and scrap sorting technology. *Resources, Conservation and Recycling* **2012**, *66*, 8–14. DOI: 10.1016/j.resconrec.2012.06.006.

(24) van den Eynde, S.; Bracquené, E.; Diaz-Romero, D.; Zaplana, I.; Engelen, B.; Duflou, J. R.; Peeters, J. R. Forecasting global aluminium flows to demonstrate the need for improved sorting and recycling methods. *Waste management (New York, N.Y.)* **2022**, *137*, 231–240. DOI: 10.1016/j.wasman.2021.11.019. Published Online: Nov. 18, 2021.

(25) Milford, R. L.; Allwood, J. M.; Cullen, J. M. Assessing the potential of yield improvements, through process scrap reduction, for energy and CO2 abatement in the steel and aluminium sectors. *Resources, Conservation and Recycling* **2011**, *55* (12), 1185–1195. DOI: 10.1016/j.resconrec.2011.05.021.

(26) International Aluminium Institute. *Aluminium Sector Greenhouse Gas Pathways to 2050*. https://international-aluminium.org/resource/aluminium-sector-greenhouse-gas-pathways-to-2050-2021/ (accessed 2022-08-06).

(27) European Aluminium. *Circular Aluminium Action Plan:A strategy for Achieving Aluminium's Full Potential for Circular Economy by 2030*. https://european-aluminium.eu/wp-content/uploads/2022/08/european-aluminium-circular-aluminium-action-plan.pdf (accessed 2024-05-08).

(28) Novelis. *Novelis and Volvo Cars Create Automotive Closed-loop Recycling System*. https://investors.novelis.com/news-releases?item=702 (accessed 2022-08-06).

(29) Karidis, A. *Covanta Boosts its Capabilities to Recover Nonferrous Metals*. https://www.waste360.com/metals/covanta-boosts-its-capabilities-recover-nonferrous-metals (accessed 2022-08-06).

(30) Material Economics. *The Circular Economy - a Powerful Force for Climate Mitigation*. https://materialeconomics.com/publications/the-circular-economy-a-powerful-force-for-climate-mitigation-1 (accessed 2024-05-08).

(31) Liu, G.; Müller, D. B. Mapping the global journey of anthropogenic aluminum: a trade-linked multilevel material flow analysis. *Environmental science & technology* **2013**, *47* (20), 11873–11881. DOI: 10.1021/es4024404. Published Online: Sep. 26, 2013.

(32) Oberhausen, G.; Zhu, Y.; Cooper, D. R. Reducing the environmental impacts of aluminum extrusion. *Resources, Conservation and Recycling* **2022**, *179*, 106120. DOI: 10.1016/j.resconrec.2021.106120.

(33) Alcoa. *Annual Report 2020*. https://investors.alcoa.com/financials/annual-reports-and-proxy-statements/default.aspx (accessed 2024-05-08).

(34) U.S. Geological Survey. *Mineral commodity summaries 1994-2022*.

(35) Pawlek, R. *Primary Aluminum Smelters of the World (2012-2022)*. https://store.lightmetalage.com/index.php?_a=product&product_id=1020 (accessed 2022-08-03).

(36) The Aluminium Association; European Aluminium; Aluminium Association of Canada; Japan Aluminium Association. *Aluminium excess capacity: Time to act*.

(37) Barber, M.; Tabereaux, A. T. The end of an era for Søderberg Technology in North and South America. In *Light Metals 2014*; Grandfield, J., Ed.; Springer eBook Collection Chemistry and Materials Science; Springer, 2016; pp 809–814. DOI: 10.1007/978-3-319-48144-9_136.

(38) Gao, B.; Wang, Z.; Shi, Z.; Hu, X., Eds. *History and Recent Developments in Aluminum Smelting in China*, 2017.

(39) Barber, M.; Tabereaux, A. T. The Evolution of Søderberg Aluminum Cell Technology in North and South America. *JOM* **2014**, *66* (2), 223–234. DOI: 10.1007/s11837-013-0855-1.

(40) International Aluminium Institute. *Perfluorocarbon (PFC) Emissions*. https://international-aluminium.org/statistics/perfluorocarbon-pfc-emissions/ (accessed 2022-06-15).

(41) Reverdy, M.; Potocnik, V. History of Inventions and Innovations for Aluminum Production. In *TMS 2020 149th Annual Meeting & Exhibition Supplemental Proceedings*; The Minerals, Metals & Materials Series; Springer International Publishing, 2020; pp 1895–1910. DOI: 10.1007/978-3-030-36296-6_175.

(42) Rusal. *Eco-Soderberg*. https://rusal.ru/en/innovation/technology/ekosoderberg/ (accessed 2022-08-03).

(43) Lauinger, D.; Billy, R. G.; Vásquez, F.; Müller, D. B. A general framework for stock dynamics of populations and built and natural environments. *Journal of Industrial Ecology* **2021**, *25* (5), 1136–1146. DOI: 10.1111/jiec.13117.

(44) International Aluminium Institute. *2019 Anode Effect Survey Report*. https://international-aluminium.org/resource/2019-anode-effect-survey-report/ (accessed 2022-08-19).

(45) Haupin, W. History of Electrical Energy Consumption by Hall-Héroult Cells. In *Hall-Héroult Centennial*; Peterson, W. S., Miller, R. E., Eds.; John Wiley & Sons, Inc, 2007; pp 106–113. DOI: 10.1002/9781118788011.ch8.

(46) International Aluminium Institute. *Primary Aluminium Smelting Power Consumption*. https://international-aluminium.org/statistics/primary-aluminium-smelting-power-consumption/ (accessed 2022-08-19).

(47) Moomaw, W.; Burgherr, P.; Heath, G.; Lenzen, M.; Nyboer, J.; Verbruggen, A. Annex II: Methodology. In *IPCC Special Report on Renewable Energy Sources and Climate Change Mitigation*; O. Edenhofer, R. Pichs-Madruga, Y. Sokona, K. Seyboth, P. Matschoss, S. Kadner, T. Zwickel, P. Eickemeier, G. Hansen, S. Schlömer, C. von Stechow, Ed., 2011.

(48) Classen, M.; Althaus, H.-J.; Blaser, S.; Scharnhorst, W. *Life Cycle Inventories of Metals:ecoinvent v2.1 report No. 10*.

(49) Mission Possible Partnership. *Closing the Gap for Aluminium Emissions:Technologies to Accelerate Deep Decarbonization of Direct Emissions*. https://missionpossiblepartnership.org/resources/ (accessed 2024-05-08).

(50) Climate Technology Centre & Network. *Inert anode technology for aluminium smelters*. https://www.ctc-n.org/technologies/inert-anode-technology-aluminium-smelters (accessed 2022-08-05).

(51) ELYSIS. *Carbon free aluminium smelting a step closer: ELYSIS advances commercial demonstration and operates at industrial scale*. https://www.elysis.com/en/carbon-free-aluminium-smelting-a-step-closer-elysis-advances-commercial-demonstration-and-operates (accessed 2022-08-05).

(52) Rusal. *Inert anode*. https://rusal.ru/en/innovation/technology/inertnyy-anod/ (accessed 2022-08-05).

(53) Luo, Z.; Soria, A. Prospective Study of the World Aluminium Industry. *1018-5593* **2008.** DOI: 10.2791/36024.

(54) Norgate, T. E.; Jahanshahi, S.; Rankin, W. J. Assessing the environmental impact of metal production processes. *Journal of Cleaner Production* **2007**, *15* (8-9), 838–848. DOI: 10.1016/j.jclepro.2006.06.018.
(55) Haraldsson, J.; Johansson, M. T. Review of measures for improved energy efficiency in production-related processes in the aluminium industry – From electrolysis to recycling. *Renewable and Sustainable Energy Reviews* **2018**, *93*, 525–548. DOI: 10.1016/j.rser.2018.05.043.
(56) Brown, C. Next generation vertical electrode cells. *JOM* **2001**, *53* (5), 39–42. DOI: 10.1007/s11837-001-0208-3.
(57) International Aluminium Institute. *Processes – Power Generation*. https://primary.world-aluminium.org/processes/power-generation/ (accessed 2022-08-25).
(58) International Aluminium Institute. *Aluminium Sector Greenhouse Gas Pathways to 2050*. https://international-aluminium.org/resource/aluminium-sector-greenhouse-gas-pathways-to-2050-2021/.
(59) Eggleston, H. S., Ed. *2006 IPCC guidelines for national greenhouse gas inventories*; Institute for Global Environmental Strategies, 2006.
(60) UNFCCC. *Global Warming Potentials (IPCC Second Assessment Report)*. https://unfccc.int/process/transparency-and-reporting/greenhouse-gas-data/greenhouse-gas-data-unfccc/global-warming-potentials (accessed 2022-08-20).
(61) World Resources Institute; World Business Council for Sustainable Development. *Greenhouse Gas Protocol, Corporate Value Chain (Scope 3) Accounting and Reporting Standard*.
(62) Trauzeddel, D.; Schmitz, W. Induktionsöfen für Kupfer, Aluminium, Zink, Magnesium, Silizium. In *Praxishandbuch Thermoprozesstechnik Band 2: Anlagen, Komponenten, Sicherheit,* 2nd ed.; Pfeifer, H., Nacke, B., Beneke, F., Eds.; Vulkan-Verl., 2011; pp 97–146.
(63) Schmitz, C., Ed. *Handbook of aluminium recycling*:*Fundamentals, mechanical preparation, metallurgical processing, plant design*, 1.th ed.; Vulkan-Verlag, 2006.
(64) Otto Junker. *Mittelfrequenz-Induktionstiegelöfen*. https://www.otto-junker.com/de/produkte-technologien/anlagen-fuer-aluminium-und-aluminiumlegierungen/giesserei_und_casthouse/mittelfrequenz-induktions-tiegeloefen/.
(65) European Commission. *Reference Document on Best Available Techniques in the Smitheries and Foundries Industry*. Integrated Pollution Prevention and Control (accessed 2020-10-12).
(66) Stephan, W.; Zitzmann, K.; Pröbstle, G.; Kapischke, J. *Effiziente Energieverwendung in der Industrie - Teilprojekt "Metallschmelzbetriebe"*:*Effiziente Energienutzung in Nicht-Eisen-Metall-Schmelzbetrieben*.
(67) Valder, G. Brennstoffbeheizte Öfen: Ein- und Zweikammerherdöfen. In *Praxishandbuch Thermoprozesstechnik Band 2: Anlagen, Komponenten, Sicherheit,* 2nd ed.; Pfeifer, H., Nacke, B., Beneke, F., Eds.; Vulkan-Verl., 2011; pp 146–156.
(68) Hillen, R. Brennstoffbeheizte Öfen: Aluminium Schachtschmelzöfen. In *Praxishandbuch Thermoprozesstechnik Band 2: Anlagen, Komponenten, Sicherheit,* 2nd ed.; Pfeifer, H., Nacke, B., Beneke, F., Eds.; Vulkan-Verl., 2011; pp 156–161.
(69) Hentschel, F. G.; Feldmann, F. Application of The Channel Induction Furnace For Melting Aluminum. *JOM* **1982**, *34* (7), 59–63. DOI: 10.1007/BF03338053.
(70) Our World in Data. *Carbon intensity of electricity*. https://ourworldindata.org/grapher/carbon-intensity-electricity?tab=chart (accessed 2022-08-20).
(71) International Energy Agency. *The Future of Hydrogen – Analysis*:*Seizing today's opportunities*. https://www.iea.org/reports/the-future-of-hydrogen (accessed 2022-08-05).
(72) Ewing, M.; Israel, B.; Jutt, T.; Hoda, T.; Stepanik, L. *Hydrogen on the path to net-zero emissions*:*Costs and climate benefits*. https://www.pembina.org/pub/hydrogen-primer.

(73) StrikoWestofen. *Der StrikoMelter Plus+ Energiesparofen*. https://www.strikowestofen.com/de-de/strikomelter-plus-energiesparofen (accessed 2022-08-24).

(74) Bacchetti, A.; Bonetti, S.; Perona, M.; Saccani, N. Investment and Management Decisions in Aluminium Melting: A Total Cost of Ownership Model and Practical Applications. *Sustainability* **2018**, *10* (9), 3342. DOI: 10.3390/su10093342.

(75) Hydrogen Council. *Hydrogen Insights:A perspective on hydrogen investment, market development and cost competitiveness*.

(76) International Energy Agency. *World Energy Outlook 2022*. https://www.iea.org/reports/world-energy-outlook-2022 (accessed 2024-05-08).

(77) Rogelj, J., D. Shindell, K. Jiang, S. Fifita, P. Forster, V. Ginzburg, C. Handa, H. Kheshgi, S. Kobayashi, E. Kriegler, L. Mundaca. Mitigation Pathways Compatible with 1.5°C in the Context of Sustainable Development. In *Global Warming of 1.5°C. An IPCC Special Report on the impacts of global warming of 1.5°C above pre-industrial levels and related global greenhouse gas emission pathways, in the context of strengthening the global response to the threat of climate change, sustainable development, and efforts to eradicate poverty*; IPCC, Ed.; Cambridge University Press, 2022; pp 93–174. DOI: 10.1017/9781009157940.004.

(78) Teske, S.; Niklas, S.; Talwar, S.; Atherton, A. 1.5 °C pathways for the Global Industry Classification (GICS) sectors chemicals, aluminium, and steel. *SN Appl. Sci.* **2022**, *4* (4), 125. DOI: 10.1007/s42452-022-05004-0. Published Online: Apr. 1, 2022.

(79) International Aluminium Institute. *Greenhouse Gas Emissions - Aluminium Sector 2018*. https://international-aluminium.org/statistics/greenhouse-gas-emissions-aluminium-sector/ (accessed 2022-07-08).

(80) Ritchie, H.; Roser, M.; Rosado, P. *Greenhouse Gas Emissions*. https://ourworldindata.org/greenhouse-gas-emissions (accessed 2022-08-03).

(81) Watari, T.; Nansai, K.; Giurco, D.; Nakajima, K.; McLellan, B.; Helbig, C. Global Metal Use Targets in Line with Climate Goals. *Environmental science & technology* **2020**, *54* (19), 12476–12483. DOI: 10.1021/acs.est.0c02471. Published Online: Sep. 11, 2020.

(82) International Aluminium Institute. *1.5 Degrees Scenario: A Model To Drive Emissions Reduction - International Aluminium Institute*. https://international-aluminium.org/resource/1-5-degrees-scenario-a-model-to-drive-emissions-reduction/ (accessed 2023-07-27).
